# Eating Style and the Frequency, Size and Timing of Eating Occasions: A cross-sectional analysis using 7-day weighed dietary records

**DOI:** 10.1038/s41598-019-51534-w

**Published:** 2019-10-22

**Authors:** Emmanouil Magklis, Laura Diane Howe, Laura Johnson

**Affiliations:** 1 MRC Integrative Epidemiology Unit at the University of Bristol, Oakfield House, Oakfield Grove, Bristol, BS82BN United Kingdom; 2 Centre for Exercise, Nutrition and Health Sciences, School for Policy Studies, University of Bristol, 8 Priory Rd, Bristol, BS81TZ United Kingdom; 3 Population Health Sciences, Bristol Medical School, Oakfield House, Oakfield Grove, Bristol, BS82BN United Kingdom

**Keywords:** Nutrition, Risk factors, Obesity, Epidemiology, Weight management

## Abstract

The tendencies to overeat in response to negative emotions (emotional eating) and environmental cues (external eating) have both been associated with BMI. However, it is unclear how they are expressed at the eating architecture level, for example, respecting frequency, timing and size of eating occasions, which could comprise ‘downstream’ specific behavioural intervention targets. In our analyses of the UK National Diet and Nutrition Survey 2000–2001, a 1-unit higher emotional eating score was associated with meals containing approximately 15 (3, 26) fewer kcals per occasion, consuming 1.4 (0.5, 2.3) more snacks per week and snacking over a 35- (16, 53) minute longer period a day. A 1-unit higher external eating score was associated with snacking over a 24- (1, 46) minute shorter period a day. Associations were independent of BMI and other potential confounders. The distinct pattern of eating architecture associated with emotional eating, suggests specific approaches to intervention, such as the number, timing and caloric content of snacks, could be considered further in experimental studies for their potential to prevent weight gain in people with a higher emotional eating tendency. Longitudinal studies and better measurement are also needed to strengthen causal inference in terms of the downstream effects of eating styles.

## Introduction

Obesity is a complex disease with an uncertain aetiology. Eating behaviour is central in determining body weight and obesity risk^1–3^. Two eating styles relevant to obesity research are eating in response to negative emotions (e.g. anxiety or fear) and eating in response to ‘external cues’ (e.g. highly palatable food or the presence of others eating)^4–6^.

Emotional eating may be a risk factor for weight gain. All prospective studies that have psychometrically assessed emotional eating have observed direct associations with BMI, even after adjustment for external eating^7–10^. The role of external eating in weight gain is less clear. Some studies report direct associations with weight gain which are attenuated after adjustment for emotional eating^7,8^. The independent association of emotional but not external eating with BMI after adjustment for each other in the same model suggests they may be overlapping traits or that emotional eating represents a higher risk style of eating for increasing BMI. To fully understand links between emotional or external eating and weight, reported food intake could reveal the behavioural expression of these eating styles. For example, a person with a high external eating score may report a tendency to snack when exposed to external stimuli but in practice how frequent are those stimuli and do they lead to measurable differences in snack frequency?

Eating architecture refers to how, rather than what, people eat^11^. It encompasses aspects such as frequency, size, timing or regularity of eating occasions. It may represent risk factors for obesity that are independent of the intake of specific foods or nutrients^12,13^. Meta-analyses of randomized controlled trials on the effects of manipulating eating frequency (EF) to promote weight loss have not suggested a protective effect of increased frequency^14,15^. However, it is not clear whether the eating frequency of specific types of eating occasions (e.g. snacks) is more important than that of others (e.g. main meals). Also, the frequency and size of eating occasions are closely related, hence interpreting evidence for size or frequency, in isolation, is challenging^16^. Finally, factors promoting weight loss may differ to those that prevent weight gain. Caloric intake per eating occasion i.e. size, has also been hypothesized as potentially contributing to the rising obesity trends^17^. It has been associated with obesity^18^ and weight gain^19,20^. Timing of intake is an emerging risk factor for body weight regulation^13^. Aspects of eating architecture associated with an eating style could be the way an eating style affects weight. Hence, they could comprise targets of behaviour interventions aimed at minimising the impact of an eating style on weight gain.

Evidence on the associations between emotional or external eating and eating architecture is scarce. Van Strien *et al*.^8^ found that among participants who reported overconsuming more than once a month, emotional eaters were consuming more frequent high caloric between-meals snacks than low emotional eaters. However, meal patterns were not assessed using detailed dietary assessment like food diaries. Nolan and Geliebter found a positive association between emotional and external eating and Night Eating Syndrome (NES) symptoms^21^, however NES is an eating disorder with features beyond eating later in the night^22^. Analysis of similar eating styles (satiety responsiveness and food responsiveness) and food intake in children aged 16–21 months showed discrete associations for different styles. Higher food responsiveness was associated with increased eating frequency whereas higher satiety responsiveness was associated with increased energy intake per eating occasion^16^. Jalkanen *et al*. found that enjoyment of food was positively associated with the number of main meals eaten by children 6–8 years. and food fussiness were, respectively, positively and negatively associated with the number of main meals eaten by children aged 6–8 years, although evidence from children is not always translatable to adults^23^. Larger studies in representative samples of adults using detailed dietary assessment are needed, analysing together the different aspects of eating architecture.

Our aim was to investigate, for the first time, the associations of emotional and external eating styles as measured by the Dutch Eating Behaviour Questionnaire (DEBQ), with the size, frequency and timing of eating, separately for meals, snacks and drinks. We used openly accessible data from the UK National Diet and Nutrition Survey (NDNS) 2000–2001, a cross-sectional survey using 7 day weighed diet diaries and DEBQ in n = 1459 UK adults aged 19–64. We hypothesized that emotional and external eating would be associated with a higher eating frequency and that emotional eating would be associated with eating later at night, since boredom, one of the emotions assessed by the DEBQ, may increase in the evening.

## Results

### Analysis sample

**Figure 1:**
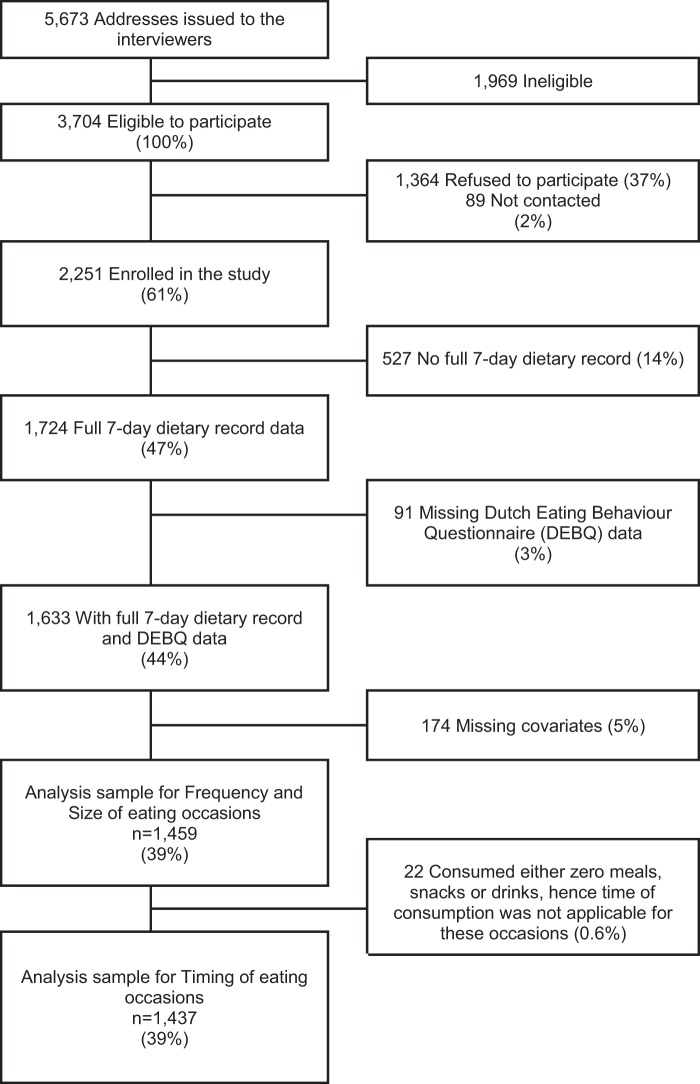
Flowchart of study participation in the National Diet and Nutrition Survey (2000–2001). Percentages are percent of eligible sample.

From the 1724 participants who completed the dietary record, 1459 had complete data on the DEBQ, size, frequency of eating occasions, and covariates. Twenty-two adults reported their eating but did not consume any meals, snacks or drinks. Hence, while having a valid value of 0 for average frequency and size of these specific types of eating occasions, they could not have valid values for the corresponding timing variables, because it’s impossible to measure the time of an event that didn’t happen, and therefore the sample for complete case analyses of the timing of specific types of eating occasion (meals, snacks or drinks) was smaller (n = 1437). Figure 1 is a flowchart of study participation. We compared our analysis sample with those excluded due to missing data in any of the variables in Supplementary Tables s2 and s3.

As seen in Supplementary Table s2, analysis sample participants (95% white, 55% women) had a median age of 41 years ((Interquantile range 25^th^–75^th^ percentile) IQR 33–53). Almost half of them had a higher education qualification and 60% had a non-manual occupation. Median BMI was 26 kg/m^2^ (IQR 23.29–29.45) and average energy intake throughout the recording week was 1774 kcal/day. Median emotional and external eating scores were 1.77 (IQR 1.23–2.39) and 2.70 (IQR 2.20–3.00) respectively, whereas median restrained eating score was 2.33 (IQR 1.44–3.11). Participants consumed any foods and drinks a median of 9 times a day (IQR 7–11)), comprising a median of 2.5 (IQR 2–3) meals, 2 (IQR 1–3) snacks and 4 (IQR 3–6) drinks on an average day. The median eating occasion contained 220 kcal (IQR 170–278), with meals containing 525 (IQR 434–631) kcal, snacks 169 (IQR 121–234) kcal and drinks 50 IQR (29–81) kcal. All eating occasions took place over a median of 825 (IQR 750–895) minutes -the equivalent of 13.8 hours- with meals and drinks spread over a longer period (of 517 and 574 minutes) when compared to snacks (155 minutes). On average participants started eating at 07:40 (IQR 06:54–08:26) and finished at 21:26 (IQR 20:38–22:15). The first meal and drink were consumed closely together at 09:39 and 09:51 respectively, with the first snack taking place on average at 13:38 (IQR 12:10–15:35). Last meals were consumed on average at 18:31 (IQR 17:39–19:29), last snacks at 16:49 (IQR 14:42–18:44) and last drinks at 20:01 (IQR 18:03–21:21). Restrained eating was weakly correlated with emotional (r = 0.33) and external eating (r = 0.19), whereas emotional and external eating were moderately correlated (r = 0.50) (Supplementary Table s10).

Participants excluded from the analyses were no different in terms of emotional or external eating, age, sex, BMI, misreporting, sleep, physical activity, restrained eating, and the size of snacks or drinks (Supplementary Tables s2 and s3). Excluded participants were more likely to have no qualifications (26 vs. 20%), to be in manual occupations (44 vs 38%) as well as to be from a non-white ethnic group (9 vs. 5%). They also ate approximately 100 fewer kcal/d, less frequently (10 fewer occasions per week made up of 1 meal, 3 snacks and 6 drinks), over a 40-min shorter period and in smaller (kcal) meal occasions by approximately 25 kcal.

### Emotional eating

Few aspects of eating architecture were associated with either emotional or external eating after adjustment for covariates (Table 1). There was evidence of association for higher emotional eating scores with smaller meals, more frequent snacking and snacking over a longer period due to both an earlier start and a later finish. In the most adjusted model, a one unit higher emotional eating was associated with meals containing 15 (95% Confidence interval (CI): 3, 26) fewer kcals, consuming 1.4 (95% CI: 0.5, 2.3) more snacks per week (driving the 1.7 (95% CI: 0, 3.4) more eating occasions a week) and eating for 35 (16, 53) more minutes per day with a, by 18 (3, 34) minutes, earlier start and a, by 16 (95% CI: 0, 33) minutes, later finish.

**Table 1:** Associations between eating style and eating architecture in the NDNS*, 2000–2001.

Eating architecture aspect	Emotional eating		External eating	
	Β^†^	(95% CI*)	B^‡^	(95% CI)
**Frequency (eating occasions/week)**				
Eating occasions	1.7	(0, 3.4)	−0.1	(−2.2, 2)
Meals	0.1	(−0.3, 0.4)	0.1	(−0.3, 0.5)
Snacks	1.4	(0.5, 2.3)	−0.3	(−1.4, 0.7)
Drinks	0.2	(−1.2, 1.6)	0	(−1.7, 1.7)
**Size (kcal/eating occasion)**				
Eating occasions	−6	(−12, 1)	2	(−7, 10)
Meals	−15	(−26, −3)	0	(−14, 14)
Snacks	−2	(−14, 9)	3	(−11, 17)
Drinks	−1	(−5, 2)	0	(−4, 4)
**Eating period (minutes)**				
Eating occasions	10	(0, 20)	6	(−7, 18)
Meals	5	(−9, 18)	3	(−14, 20)
Snacks	35	(16, 53)	−24	(−46, −1)
Drinks	5	(−18, 27)	10	(−18, 38)
**Time of first eating occasion (minutes)**				
Eating occasions	−5	(−13, 2)	−2	(−12, 7)
Meals	−4	(−17, 8)	1	(−14, 17)
Snacks	−18	(−34, −3)	12	(−7, 31)
Drinks	−4	(−22, 14)	0	(−22, 22)
**Time of last eating occasion (minutes)**				
Eating occasions	4	(−2, 11)	3	(−4, 11)
Meals	0	(−7, 8)	4	(−5, 14)
Snacks	16	(0, 33)	−11	(−32, 9)
Drinks	0	(−14, 15)	10	(−8, 28)

^*^NDNS, National Diet and Nutrition Survey; CI, Confidence Interval.

^†^B is the unstandardized coefficient of Emotional eating from the multiple linear regression of the respective eating architecture aspect on Emotional eating score, External eating score, Restrained eater (Yes, No), Age, Sex (Male or Female), Ethnicity (White, non-white), Occupational social class (manual, non-manual), Educational attainment (Highest educational qualification: Degree or equivalent, Higher education or GCE A level equivalent, GCSE grades A-E or equivalent, No qualifications or other qualifications), Sleep duration (short, average, long), Smoking (Currently a smoker, Past smoker, Never a smoker), Energy intake (Average over 7 days of energy intake, excluding supplements and alcohol (kcal)), Physical activity (Average minutes spent daily on at least moderate activity), Currently on a diet to lose weight (Yes, No), Eating affected by being unwell (Yes, No), BMI, Misreporting category (underreporting, normal reporting, overreporting).

^‡^B is the unstandardized coefficient of External eating from the multiple linear regression of the respective eating architecture aspect on External eating score, Emotional eating score, and the remaining covariates as in^†^.

The evidence of association of emotional eating with snack frequency was consistent across all models (See Supplementary Table s8), in contrast the crude association with smaller meal size was attenuated by adjustment for covariates in the most adjusted model from 37 to 15. Evidence for the association with snacking period was weaker in the crude models, with the effect estimate increasing after the adjustment for age and sex from 18 (95% CI: 4,33) to 35 (95% CI: 16,53) minutes a day. In our sensitivity analyses, associations between emotional eating and eating architecture were robust. Removing BMI, misreporting category or external eating from the fully adjusted model did not seem to considerably alter the associations observed (Supplementary Tables s4 and s5).

### External eating

In contrast, external eating showed no robust evidence of association with size or frequency of eating and was only associated with the timing of snacks. A one unit increase in external eating was associated with a 24-minute (95% CI: −46, −1) shorter snacking period, with a 12-minute (95% CI: −7, 31) later start and a 11-minute earlier (95% CI: −32,9) finish time (Table 1).

While the crude or age and sex adjusted models showed some evidence that increased external eating is associated with an increased frequency of eating, estimates were attenuated in size and statistical significance after the inclusion of further covariates (Supplementary Table s9). In the age and sex adjusted model a 1-unit higher external eating was associated with 4.9 more eating occasions per week, comprising 1 meal, 1.6 snacks and 1.9 drinks but these estimates were reduced to 0 after further adjustment. While the crude model suggested a one-unit change in external eating was associated with larger meals and snacks by 17 and 14 kcal respectively, adjustment for age and sex attenuated the associations, primarily because higher external eating was associated with a younger age which, in turn, was associated with a smaller meal and snack size.

### Interactions

There was no evidence that restrained eating modified the associations of either emotional or external eating with eating architecture (Supplementary Table S6). Investigation of effect measure modification by sex, provided very weak evidence that the associations between emotional eating, snacking frequency and timing observed in the full sample were driven by women. However, p-values for interaction were considerably large and thus we cannot rule this out as a chance finding (Supplementary Table S7).

## Discussion

In the present study, we evaluated cross-sectional associations of emotional and external eating with aspects of eating architecture including the timing, size and frequency of eating among 1469 British adults participating in the UK National Diet and Nutrition Survey 2000–2001. After adjustment for a wide range of covariates, a greater tendency towards emotional eating was associated with smaller meals and more frequent snacking over a longer period every day -with an earlier start and a later finish time. External eating was associated with a shorter eating period. There was very weak evidence that sex modified the associations between emotional eating and snacking with associations of emotional eating with snacking frequency being stronger among women.

Previous studies of eating styles and food intake are generally limited to the type of food or nutrient consumed whereas how people eat (eating architecture) may be a more distinct indicator of behavioural traits. To our knowledge, only one study has previously explored the association between emotional eating and snacking, showing more frequent consumption of high caloric between-meals snacks among emotional eaters^8^. Our findings are in line with that finding, observing 1.3 more snacks per week with a one-unit higher emotional eating. If not compensated for by reductions in energy intake elsewhere it could mediate the association of emotional eating with weight gain. However, we also observed an association between emotional eating and smaller meal sizes, suggesting that adjustment is made to some extent for the extra energy intake from more frequent snacks. As participants ate on average 18 meals a week, a 13-kcal reduction in meal size would be equal to 234 fewer kcal over a week. Snacks contained on average 169 kcal per occasion so an increase of 1.3 occasions per week would be equal to 219 more kcal over a week. Thus, total energy intake may not be excessive among emotional eaters owing to the increased snacking being cancelled out by the smaller meal sizes.

We found no evidence that our data were compatible with an association between external eating and most aspects of eating architecture. This is broadly in line with the literature suggesting that external eating is not be an obesogenic eating style^7,8^. The smaller period over which snacks seemed to be consumed over a day was not accompanied by a difference in the number or caloric content of snacks. In that regard, while this may be a true finding, it is unlikely to bear an effect on weight. The period alone over which a given number of snacks are spread, should not be relevant to weight gain.

Our findings, including the null, should be interpreted with caution. On one hand, they are compatible with a previous report by Strien *et al*.^8^ and make intuitive sense: individuals more likely to eat when experiencing a variety of emotions can be expected to eat more frequently -especially smaller ‘ad-hoc’ eating occasions, which would be categorised as snacks. However, several of our variables are measured with error. For example, snacks have been found to be considerably underreported^24–27^. If that underreporting was higher with either higher snack intakes, BMI or emotional/external eating (e.g. because high emotional/external eaters may be less likely to report their full snack intake than low emotional/external eaters), the association between eating style and snack intake would be underestimated. Not enough evidence is available on what determines eating architecture misreporting. Reassuringly, Poppitt *et al*., found that snacks were similarly underreported by participants with and without obesity^25^. We also found that TEI/EER was not associated to emotional eating (r = −0.024 (p = 0.36)). Hence snack misreporting may not be systematic according to emotional eating (to the extent that snack misreporting is proportional to general misreporting). Similarly, quantitative information on the errors involved in assessing eating style with questionnaires such as the DEBQ is not available, mainly due to the absence of a reference method. Increasing underreporting of emotional eating with higher emotional eating (e.g. less willingness to report true emotional eating the higher it is due to social desirability bias) or snack intake would lead to an overestimation of the association. Also, if emotional eating and snack intake underreporting were dependent, for example if participants underreporting the one, tend to do so for the other, either because of memory, social desirability or other personal characteristics, the true association would also be overestimated^28^. Finally, overestimation is also possible when there are several error-prone variables in a multivariable model^29,30^. The direction of the net bias due to measurement error in emotional eating, eating architecture and some of our most error-prone covariates such as the other eating styles, physical activity and total energy intake is difficult to predict. However, we took several steps to control for its effects. First, we estimated misreporting of energy intake in our data and included it as a covariate in our analyses. We also adjusted for total energy intake which, although not generally considered as a method to address measurement error^30^, has been shown to remove some of the error in absolute estimates of nutrient intakes^31^ and therefore could have removed some of the error in the eating architecture variables. In addition, most of the covariates we adjusted for are also related to reporting errors, and that helps to reduce differential error related due to these factors, although possibly at the expense of increasing nondifferential error^28^. Finally, averaging over 7 days of intake reduces the random variation of the eating architecture variables.

Residual confounding due to unmeasured confounders or poorly measured ones cannot be ruled out. However, it is a strength of our study that several potential confounders were measured and adjusted for. There is contention in the literature on the causal relationships between emotional eating, external eating and other traits. For example, it has been suggested that both emotional and external eating are caused by or manifestations of a “common” trait like a lack of control over food (uncontrolled eating^32^) or a general tendency to eat in response to emotional, external and other cues (cue responsiveness^33^). We confirmed the previously reported high correlation between emotional and external eating (Supplementary Table s10) but our analyses cannot shed additional light to this contention because there were no measures of such “common” traits in NDNS. Not adjusting for external and emotional eating, respectively, did not materially alter our conclusions suggesting that they may not be important confounders of one another when the outcome is eating architecture (Supplementary Tables s4 and s5).

Participants excluded from our most adjusted model consumed somewhat fewer snacks and spent a smaller part of the day eating. Selection of individuals into an analysis according to their outcome (in this case eating architecture) will generally bias the complete case analysis, so a degree of selection bias is possible. Finally, the directionality of associations between eating style and eating architecture is unknown and cannot be probed with a cross-sectional dataset. Choosing eating architecture as the dependent variable in our analyses was theoretical, indicating our interest to explore how eating style could be expressed. Future research could explore the direction of associations by using randomised trial or prospective observational designs.

The use of weighed dietary records is among the strengths of our study. They are completed in real-time and don’t rely on memory as do 24-hour dietary recalls (24-HDRs) and food frequency questionnaires (FFQs). They have been shown to greatly outperform FFQs and capture on average 80% of true energy intake compared with direct observation or doubly labelled water estimates of energy expenditure^25,34–36^. Other strengths of our study include the large nationally representative sample of British adults, the systematic way of defining eating occasions, meals, drinks and snacks (versus participants self-defining) and the 7 days of intake recorded. Finally, separating meals from snacks and drinks is a strength as they are different types of eating occasions with diverse behavioural correlates^37–39^, which was supported by the divergent associations we observed.

Future research should build on our analyses to confirm our findings and enable uncertainty around the causal nature and mechanisms linking emotional eating and weight gain to be elucidated. To that direction, observational studies will benefit from longitudinal data collection and more accurate measures of food intake (such as passive dietary assessment methods, perhaps including continuous on-body cameras (sense-cam)^40^). Already conducted validation studies that have used direct observation or duplicate portions, if analysed accordingly, may also provide valuable information on the measurement error properties of variables such as size, frequency and timing of eating occasions^25,41^. Assessment of constructs related to emotional and external eating (e.g. uncontrolled eating or personality traits) may help clarify what do emotional eating scales represent^33^. Naturalistic study designs using ecological momentary assessment^42^ are a promising development as they may come with fewer or independent errors of assessment. However, they add considerable burden to the participants, on top of that of a detailed dietary assessment. Bias analysis may also benefit future studies with several error-prone variables^28,30^.

In view of better evidence of links between styles of eating and aspects of eating architecture, these aspects could be the target of interventions aimed at individuals or groups of individuals at increased risk due to their eating style. Interventions aimed at both emotional eating and eating architecture could be the most effective. For example, if emotional eating is robustly associated with snacking (but not as much with meal size), an intervention targeting snacking, combined with an intervention targeting emotional eating (such as emotional regulation^43^), but without as much focus on meal size, may be the most effective one. This may be seen as providing an additional degree of tailoring or ‘matching’ the treatment of obesity to groups of individuals according to their eating styles and eating architecture^43^. Preliminary testing of an intervention tailored according to the appetitive traits of participants was recently conducted^44^.

Our study was the first to contribute to the systematic exploration of the associations between eating style and eating architecture. Emotional eating was associated with additional snacking, starting earlier and finishing later within a day and a smaller meal size. External eating was only associated with a shorter daily period of snacking. Limitations related to measurement and confounding do not allow causal inference regarding the expression of eating style at the level of eating architecture. The patterns of overeating associated with different eating styles should be explored further as a potential intervention target to prevent weight gain in people with these eating styles.

## Methods

### Study design and participants

The UK National Diet and Nutrition Survey (NDNS) of adults aged 19–64 was a cross-sectional survey aimed at assessing the dietary habits and nutritional status of a nationally representative sample of adults living in private households in the UK, derived using a multistage random probability design. The study methodology has been described in detail elsewhere^45^. In short, fieldwork took place in 4 waves between July 2000 and June 2001 and included a face-to-face interview to assess socio-demographic and lifestyle characteristics, anthropometric and blood pressure measurements, a venous blood sample, a 24 h urine collection, a 7-day record of physical activity and a 7-day weighed food intake record. The NDNS sample size determination has been described elsewhere. This is a secondary data analysis study of an already available (fixed) data set. Our analysis sample was comprised of 1459 individuals for eating frequency and eating occasion size (64% of those enrolled) (Fig. 1).

The Medical Research Council Human Nutrition Research, Cambridge obtained full approval from the South Thames Multi-centre Research Ethics Committee (MREC) in August 1999. Having achieved MREC approval, applications were then made to the ten Local Research Ethics Committees (LRECs) that covered the geographical areas selected for the fieldwork. Full ethics approval from the MREC and all LRECs was achieved before fieldwork started. Data collection was conducted in line with the regulations of the MREC and LRECs. Participation was voluntary and informed consent was obtained in stages for each component of the study and participants were free to withdraw from data collection at any stage. Anonymised archived data on the UK data service was accessed after agreement to the end user license and was used according to its terms and conditions (ukdataservice.ac.uk/get-data/how-to-access/conditions).

### Emotional, external and restrained eating

The Dutch Eating Behaviour Questionnaire (DEBQ)^6^ assesses the emotional (DEBQ-Em, 13 items), external (DEBQ-Ext, 10 items) and restrained eating styles (DEBQ-R, 10 items) using a 5-point Likert scale for each item (1 = never, 2 = seldom, 3 = sometimes, 4 = often, 5 = very often). Adherence to each eating style was calculated as the mean score of its items. The DEBQ-Em and DEBQ-Ext, are the exposures in our analyses and were used as continuous variables. To examine effect measure modification by restrained eating, DEBQ-R was dichotomised as high (>3) and low (≤3)^46^.

### Dietary assessment and eating architecture

The dietary assessment procedure is described in detail elsewhere^45^. In short, participants were provided with a diary for recording all food and drink consumed over 7 days weighed using a set of calibrated digital scales. Intake of dietary supplements was recorded both in the diaries and the initial interview. Weight information was also obtained from the packaging, by buying duplicate items, or using standard portion sizes for occasions where the scales could not be used. Time of consumption was recorded for all eating occasions. Participants were also asked whether they were currently dieting to lose weight or whether their eating had been affected by being unwell. All foods/drinks consumed at a unique time were considered an eating occasion. Each eating occasion was also classified as a meal, a snack or a drink, using a food-based definition, described in detail elsewhere^47^.

Aspects of eating architecture examined were a) total eating frequency (number of eating occasions per week), b) mean size of eating occasions (kcal/occasion), c) mean daily eating period (the duration between the first and last eating occasions within a day (minutes)), d) mean daily timing of first eating occasion (minutes from midnight) and e) mean daily timing of final eating occasion (minutes from midnight). All aspects were calculated separately for meals, snacks, drinks as well as for ‘eating occasions’, regardless of them being a meal, a snack or a drink, adding up to 20 dependent variables. The number of eating occasions, meals, snacks and drinks was counted each day and the mean daily frequency was computed over a week. Eating occasion, meal, snack and drink size (kcal) was calculated first as the mean of eating occasions within a day and then the mean daily size of eating occasions was computed across 7 days. Mean total daily energy intake over 7 days of intake, excluding supplements and alcohol was also calculated. The time of the first and last eating occasions as reported for each day were converted to continuous variables minutes from midnight and then the mean over the 7 days was used in the regression analyses and then back transformed to clock time to facilitate interpretation. The duration of the eating period (minutes) was obtained by subtracting the first from the last time of eating (see Supplementary Information, Eating architecture syntax).

### Covariates

Body weight was measured to the nearest 0.1 kg and height to the nearest 0.1 cm and body mass index (BMI) was calculated (kg/m^2^). From 7-day physical activity diaries the time spent on moderate to vigorous activities was computed and averaged across the 7 days, as well as overnight sleep duration, which was then grouped as sleeping for less than 7 hours, 7–8 hours or more than 8 hours, as often categorised in sleep research^48^. Ethnicity was self-identified as belonging to one of 9 different ethnic groups and collapsed into white or non-white similar to existing literature to reduce the number of strata with very few observations. Occupational social class was defined using the Registrar General’s Standard Occupational Classification, Volume 4, TSO (2001) and was modelled as manual vs. non-manual to create strata with sufficient observations. Educational attainment was based on the participant’s self-reported highest qualification and collapsed into 4 groups to create strata of a similar education background with more observations each (Degree or equivalent; GCE A level equivalent; GCSE grades A-E or equivalent; or no/other qualifications). Participants were categorised according to their smoking habits as current, former or non-smokers. Reported total energy intake (TEI) was divided by estimated energy requirements (EER) (TEI/EER) to assess misreporting. Estimated energy requirements were calculated using the Institute of Medicine equations^49^. Values below 0.71 were taken as indicative of under-reporting and values above 1.29 as indicative of over-reporting^46^.

### Statistical analysis

Eating architecture variables were generated using syntax written and run in IBM® SPSS® Statistics for Windows, Version 22.0 (IBM Corp., Armonk, NY, USA) (See Supplementary Information, Eating Architecture syntax).

Continuous variables were reported as median (Interquantile range (IQR) 25th, 75th percentile) due to several variables being positively skewed and categorical variables were reported as counts and percentages (%). Participants with missing data in any of the variables examined were excluded from the analyses. For each of the variables, we compared those included and those excluded and reported p-values for the two-sided Pearson’s chi-square test for categorical variables and two-sided Mann-Whitney’s U test for continuous variables. Correlations between the emotional, external and restrained eating scales were estimated using Pearson’s correlation coefficient.

We examined the associations between emotional eating and each of the 20 aspects of eating architecture using multiple regression models while adjusting for (a) no other variables, (b) only sex, (c) only age and sex, (d) age, sex, ethnicity, occupational social class, educational attainment, sleep duration, smoking, DEBQ-R (>3 or ≤3), DEBQ-external eating score, mean total daily energy intake, physical activity, current dieting to lose weight, self-report of eating being affected by being unwell, BMI, and energy intake misreporting, (e) the variables in (d) excluding BMI, (f) the variables in (d) excluding misreporting and (g) the variables in (d) excluding external eating. Each architecture variable was investigated independently of the others, i.e. there was never more than one eating architecture variable in a model at a time.

We considered the variables in set (d) as potential confounders. There are few studies on possible determinants of the DEBQ constructs. Hence, to arrive at our list of potential confounders, we examined the list of variables available in the NDNS and identified those that could be causes of both eating style and eating architecture based on the published literature and the authors’ expertise.

Adjustment for covariates sets (e), (f) and (g) represent sensitivity analyses over (d), since BMI, misreporting and external eating, could be mediating rather than confounding the emotional eating – eating architecture associations or act as colliders (i.e. caused by both eating style and eating architecture)^50^. The associations of external eating with eating architecture were examined in the same way, with emotional eating instead of external eating in covariate sets (d), (e), (f) and (g).

Sex and restrained eating were explored as potential effect measure modifiers of the eating style-eating architecture associations by adding the respective product term (i.e. emotional eating × sex, external eating × sex, restrained eating × emotional eating, restrained eating × external eating) to the most-adjusted models (covariate set (d)) and performing a likelihood ratio test at the α = 0.05 level comparing the models with and without the interaction term.

All regression coefficients (B) reported are unstandardized with a 95% confidence interval. To ensure that the assumption of normality did not affect the performance of the models, we inspected Q-Q plots of the residuals of each model. All analyses, other than the generation of the eating architecture variables, were performed using StataCorp. 2015. Stata Statistical Software: Release 14. College Station, TX: StataCorp LP. Reporting of research followed the STROBE-nut guidelines (See Supplementary Table S1).

## Supplementary information

Supplementary information

## Data Availability

The NDNS data that support the findings of this study are publicly available from the UK Data Archive^51^ which was accessed using its catalogue, Discover, hosted at the UK Data Service^52^ on December 2016.
