# Supplementary material for: Eating Style and the Frequency, Size and Timing of Eating Occasions: A cross-sectional analysis using 7-day weighed dietary records
Source: Sci Rep. 2019 Oct 22;9:15133. doi: 10.1038/s41598-019-51534-w (PMC6805948; doi:10.1038/s41598-019-51534-w)
Supplement: Supplementary file 1 — Supplementary information [file 41598_2019_51534_MOESM1_ESM.docx]

**Eating Style and the Frequency, Size and Timing of Eating Occasions: A cross-sectional analysis using 7-day weighed dietary records**

**Emmanouil Magklis**^1,2,*^ **, Laura Diane Howe**^1,3^ **, and Laura Johnson**^1,2^

^1^MRC Integrative Epidemiology Unit at the University of Bristol, Oakfield House, Oakfield Grove, Bristol, BS82BN, United Kingdom

^2^Centre for Exercise, Nutrition and Health Sciences, School for Policy Studies, University of Bristol,
8 Priory Rd, Bristol, BS81TZ, United Kingdom

^3^Population Health Sciences, Bristol Medical School, Oakfield House, Oakfield Grove, Bristol, BS82BN, United Kingdom

*[manos.magklis@bristol.ac.uk](mailto:manos.magklis@bristol.ac.uk)

**Supplementary Information**

# Table s1

**Table s1.** STROBE-nut: An extension of the STROBE statement for nutritional epidemiology

Lachat C et al. (2016) STrengthening the Reporting of OBservational studies in Epidemiology – Nutritional Epidemiology (STROBE-nut): an extension of the STROBE statement. Plos Medicine 13(6) <http://dx.doi.org/10.1371/journal.pmed.1002036> [pdf](http://journals.plos.org/plosmedicine/article/asset?id=10.1371%2Fjournal.pmed.1002036.PDF) or [online](http://journals.plos.org/plosmedicine/article?id=10.1371/journal.pmed.1002036) version.

| **Item** | **Item nr** | **STROBE recommendations** | **Extension for Nutritional Epidemiology studies (STROBE-nut)** | **Reported on page #** |
| --- | --- | --- | --- | --- |
| **Title and**  **abstract** | 1 | (a) Indicate the study’s design with a commonly used term in the title or the abstract.  (b) Provide in the abstract an informative and balanced summary of what was done and what was found. | **nut-1** State the dietary/nutritional assessment method(s) used in the title, abstract, or keywords. | 1-3 |
| **Introduction** |  |  |  |  |
| Background rationale | 2 | Explain the scientific background and rationale for the investigation being reported. |  | 3-5 |
| Objectives | 3 | State specific objectives, including any pre-specified hypotheses. |  | 5 |
| **Methods** |  |  |  |  |
| Study design | 4 | Present key elements of study design early in the paper. |  | 5 |
| Settings | 5 | Describe the setting, locations, and relevant dates, including periods of recruitment, exposure, follow-up, and data collection. | **nut-5** Describe any characteristics of the study settings that might affect the dietary intake or nutritional status of the participants, if applicable. | 5-7 |
| Participants | 6 | a) Cohort study—Give the eligibility criteria, and the sources and methods of selection of participants. Describe methods of follow-up.  Case-control study—Give the eligibility criteria, and the sources and methods of case ascertainment and control selection. Give the rationale for the choice of cases and controls.  Cross-sectional study—Give the eligibility criteria, and the sources and methods of selection of participants.  (b) Cohort study—For matched studies, give matching criteria and number of exposed and unexposed.  Case-control study—For matched studies, give matching criteria and the number of controls per case. | **nut-6** Report particular dietary, physiological or nutritional characteristics that were considered when selecting the target population. | 5,9 |
| Variables | 7 | Clearly define all outcomes, exposures, predictors, potential confounders, and effect modifiers. Give diagnostic criteria, if applicable. | **nut-7.1** Clearly define foods, food groups, nutrients, or other food components.  **nut-7.2** When using dietary patterns or indices, describe the methods to obtain them and their nutritional properties. | 6-9 |

| Data sources - measurements | 8 | For each variable of interest, give sources of data and details of methods of assessment (measurement). Describe comparability of assessment methods if there is more than one group. | **nut-8.1** Describe the dietary assessment method(s), e.g., portion size estimation, number of days and items recorded, how it was developed and administered, and how quality was assured. Report if and how supplement intake was assessed.  **nut-8.2** Describe and justify food composition data used. Explain the procedure to match food composition with consumption data. Describe the use of conversion factors, if applicable.  **nut-8.3** Describe the nutrient requirements, recommendations, or dietary guidelines and the evaluation approach used to compare intake with the dietary reference values, if applicable.  **nut-8.4** When using nutritional biomarkers, additionally use the STROBE Extension for Molecular Epidemiology (STROBE-ME). Report the type of biomarkers used and their usefulness as dietary exposure markers.  **nut-8.5** Describe the assessment of nondietary data (e.g., nutritional status and influencing factors) and timing of the assessment of these variables in relation to dietary assessment.  **nut-8.6** Report on the validity of the dietary or nutritional assessment methods and any internal or external validation used in the study, if applicable. | 6-9 |
| --- | --- | --- | --- | --- |

| Bias | 9 | Describe any efforts to address potential sources of bias. | **nut-9** Report how bias in dietary or nutritional assessment was addressed, e.g., misreporting, changes in habits as a result of being measured, or data imputation from other sources. | 8, 14, 15 |
| --- | --- | --- | --- | --- |
| Study Size | 10 | Explain how the study size was arrived at. |  | 5 |
| Quantitative variables | 11 | Explain how quantitative variables were handled in the analyses. If applicable, describe which groupings were chosen and why. | **nut-11** Explain categorization of dietary/nutritional data (e.g., use of N-tiles and handling of nonconsumers) and the choice of reference category, if applicable. | 6-9 |
| Statistical  Methods | 12 | (a) Describe all statistical methods, including those used to control for confounding  (b) Describe any methods used to examine subgroups and interactions.  (c) Explain how missing data were addressed.  (d) Cohort study—If applicable, explain how loss to follow-up was addressed.  Case-control study—If applicable, explain how matching of cases and controls was addressed.  Cross-sectional study—If applicable, describe analytical methods taking account of sampling strategy.  (e) Describe any sensitivity analyses. | **nut-12.1** Describe any statistical method used to combine dietary or nutritional data, if applicable.  **nut-12.2** Describe and justify the method for energy adjustments, intake modeling, and use of weighting factors, if applicable.  **nut-12.3** Report any adjustments for measurement error, i.e,. from a validity or calibration study. | 6-9 |
| **Results** |  |  |  |  |
| Participants | 13 | (a) Report the numbers of individuals at each stage of the study—e.g., numbers potentially eligible, examined for eligibility, confirmed eligible, included in the study, completing follow-up, and analyzed.  (b) Give reasons for non-participation at each stage.  (c) Consider use of a flow diagram. | **nut-13** Report the number of individuals excluded based on missing, incomplete or implausible dietary/nutritional data | 9 and Figure 1 |
| Descriptive data | 14 | (a) Give characteristics of study participants (e.g., demographic, clinical, social) and information on exposures and potential confounders  (b) Indicate the number of participants with missing data for each variable of interest  (c) Cohort study—Summarize follow-up time (e.g., average and total amount) | **nut-14** Give the distribution of participant characteristics across the exposure variables if applicable. Specify if food consumption of total population or consumers only were used to obtain results. | 1, 2 of Additional File 3 |
| Outcome data | 15 | Cohort study—Report numbers of outcome events or summary measures over time.  Case-control study—Report numbers in each exposure category, or summary measures of exposure.  Cross-sectional study—Report numbers of outcome events or summary measures. |  | 1, 2 of Additional File 3 |
| Main results | 16 | (a) Give unadjusted estimates and, if applicable, confounder-adjusted estimates and their precision (e.g., 95% confidence interval).  Make clear which confounders were adjusted for and why they were included  (b) Report category boundaries when continuous variables were categorized.  (c) If relevant, consider translating estimates of relative risk into absolute risk for a meaningful time period. | **nut-16** Specify if nutrient intakes are reported with or without inclusion of dietary supplement intake, if applicable. | 9-13 of main manuscript and Additional File 3 |
| Other analyses | 17 | Report other analyses done—e.g., analyses of subgroups and interactions and sensitivity analyses. | **nut-17** Report any sensitivity analysis (e.g., exclusion of misreporters or outliers) and data imputation, if applicable. | 9-13 of main manuscript and Additional File 3 |
| **Discussion** |  |  |  |  |
| Key results | 18 | Summarize key results with reference to study objectives. |  | 13 |
| Limitation | 19 | Discuss limitations of the study, taking into account sources of potential bias or imprecision. Discuss both direction and magnitude of any potential bias. | **nut-19** Describe the main limitations of the data sources and assessment methods used and implications for the interpretation of the findings. | 13-15 |
| Interpretation | 20 | Give a cautious overall interpretation of results considering objectives, limitations, multiplicity of analyses, results from similar studies, and other relevant evidence. | **nut-20** Report the nutritional relevance of the findings, given the complexity of diet or nutrition as an exposure. | 13-16 |
| Generalizability | 21 | Discuss the generalizability (external validity) of the study results. |  |  |
| **Other information** |  |  |  |  |
| Funding | 22 | Give the source of funding and the role of the funders for the present study and, if applicable, for the original study on which the present article is based. |  | 18 |
| *Ethics* |  |  | **nut-22.1** Describe the procedure for consent and study approval from ethics committee(s). | 17 |
| *Supplementary material* |  |  | **nut-22.2** Provide data collection tools and data as online material or explain how they can be accessed. | 9 and Additional File 1 |

# Tables s2-s10

| **Table s2.** Eating style and eating architecture in the NDNS (2000-2001), according to inclusion in analysis. | | | | | | |
| --- | --- | --- | --- | --- | --- | --- |
|  | **Excluded*** | |  | **Included** | | **p**** |
| **Characteristic** | **n** | **Median  (25^th^-75^th^ percentile)** |  | **n** | **Median  (25^th^-75^th^ percentile)** |  |
| **Eating style** |  |  |  |  |  |  |
| Emotional eating score | 360 | 1.8 (1.2-2.3) |  | 1459 | 1.8 (1.2-2.4) | 0.88 |
| External eating score | 360 | 2.6 (2.2-3) |  | 1459 | 2.7 (2.2-3) | 0.20 |
| **Eating architecture aspect** |  |  |  |  |  |  |
| **Frequency** |  |  |  |  |  |  |
| Number of  eating occasions/week | 265 | 51 (39 - 66) |  | 1459 | 62 (48 - 76) | <0.001 |
| Number of meals/week | 265 | 17 (14 - 20) |  | 1459 | 18 (14 - 21) | <0.001 |
| Number of snacks/week | 265 | 13 (7 - 18) |  | 1459 | 16 (12 - 23) | <0.001 |
| Number of drinks/week | 265 | 22 (15 - 34) |  | 1459 | 28 (19 - 40) | <0.001 |
| **Size** |  |  |  |  |  |  |
| Kcal per eating occasion | 265 | 237 (179 - 290) |  | 1459 | 217 (170 - 278) | 0.03 |
| Kcal per meal | 265 | 499 (421 - 610) |  | 1459 | 525 (434 - 631) | 0.02 |
| Kcal per snack | 265 | 173 (111 - 239) |  | 1459 | 169 (121 - 234) | 0.86 |
| Kcal per drink | 265 | 53 (27 - 84) |  | 1459 | 50 (29 - 81) | 0.43 |
| **Eating period (min)** |  |  |  |  |  |  |
| Eating occasions | 287 | 785 (701 - 853) |  | 1437 | 825 (750 - 895) | <0.001 |
| Meals | 287 | 460 (313 - 588) |  | 1437 | 517 (365 - 619) | <0.001 |
| Snacks | 287 | 93 (8 - 218) |  | 1437 | 155 (31 - 305) | 0.001 |
| Drinks | 287 | 466 (273 - 688) |  | 1437 | 574 (353 - 756) | <0.001 |
| **Time of first eating occasion (clock time)** |  |  |  |  |  |  |
| Eating occasions | 287 | 07:49 (07:10 - 08:27) |  | 1437 | 07:40 (06:54 - 08:26) | 0.004 |
| Meals | 287 | 10:07 (08:41 - 12:30) |  | 1437 | 09:51 (08:22 -12:11) | 0.022 |
| Snacks | 287 | 13:38 (12:10 - 15:35) |  | 1437 | 13:32 (11:41 - 15:22) | 0.308 |
| Drinks | 287 | 10:03 (08:07 - 13:08) |  | 1437 | 09:39 (07:59 - 12:12) | 0.025 |
| **Time of last eating occasion (clock time)** |  |  |  |  |  |  |
| Eating occasions | 287 | 21:00 (19:57 - 21:51) |  | 1437 | 21:26 (20:38 - 22:15) | <0.001 |
| Meals | 287 | 18:12 (17:18 - 19:04) |  | 1437 | 18:31 (17:39 - 19:29) | 0.001 |
| Snacks | 287 | 16:04 (13:50 - 18:10) |  | 1437 | 16:49 (14:42 - 18:44) | 0.015 |
| Drinks | 287 | 19:16 (17:13 - 20:58) |  | 1437 | 20:01 (18:03 - 21:21) | <0.001 |
| * Participants with available data in the respective variable but excluded from our analysis because of missing data in other covariates of the most adjusted models.  ** Mann-Whitney p-value. | | | | | | |

| **Table s3.** Other participant characteristics in the NDNS (2000-2001), according to inclusion in analysis. | | | | | | | | |
| --- | --- | --- | --- | --- | --- | --- | --- | --- |
| **Characteristic** | **Excluded*** | |  | **Included** | |  | **p**** |  |
|  | **n** | **Median  (25^th^-75^th^ percentile) or percentage** |  | **n** | **Median  (25th-75th percentile)  or percentage** |  |  |  |
| Sex | 792 |  |  | 1459 |  |  | 0.75 |  |
| Men | 351 | 44% |  | 657 | 45% |  |  |  |
| Women | 441 | 56% |  | 802 | 55% |  |  |  |
| Age | 792 | 40  (31 - 52) |  | 1459 | 41  (33 - 53) |  | 0.07 |  |
| BMI | 329 | 26.3  (23.3 - 29.7) |  | 1459 | 26.2  (23.3 - 29.5) |  | 0.82 |  |
| Dieting | 792 |  |  | 1459 |  |  | 0.82 |  |
| Yes | 144 | 18% |  | 271 | 19% |  |  |  |
| No | 648 | 82% |  | 1188 | 81% |  |  |  |
| Eating affected by being unwell | 245 |  |  | 1459 |  |  | 0.01 |  |
| No | 204 | 83% |  | 1305 | 89% |  |  |  |
| Yes | 41 | 17% |  | 154 | 11% |  |  |  |
| TEI:EER ratio | 92 |  |  | 1459 |  |  | 0.32 |  |
| ≤0.70 | 42 | 46% |  | 762 | 52% |  |  |  |
| 0.71-1.29 | 50 | 54% |  | 687 | 47% |  |  |  |
| ≥1.30 | 0 | 0% |  | 10 | 1% |  |  |  |
| Restrained eating score | 360 | 2.3  (1.6 - 3.1) |  | 1459 | 2.3  (1.4 - 3.1) |  | 0.80 |  |
| Sleep duration | 199 |  |  | 1459 |  |  | 0.28 |  |
| Short (<7 hrs/day) | 20 | 10% |  | 148 | 10% |  |  |  |
| Average (7-8 hrs/day) | 56 | 28% |  | 490 | 34% |  |  |  |
| Long (>8 hrs/day) | 123 | 62% |  | 821 | 56% |  |  |  |
| Smoking status | 792 |  |  | 1459 |  |  | 0.001 |  |
| Never a smoker | 246 | 33% |  | 453 | 30% |  |  |  |
| Past smoker who quit | 243 | 31% |  | 524 | 39% |  |  |  |
| Current smoker | 287 | 36% |  | 482 | 31% |  |  |  |
| Energy intake/day*** (kcal) | 265 | 1567  (1215 – 1890) |  | 1459 | 1774  (1434 – 2160) |  | <0.001 |  |
| Physical activity (min of MVPA/day) | 199 | 26 (4 - 121) |  | 1459 | 34 (9 - 99) |  | 0.42 |  |
| Occupational social class | 739 |  |  | 1459 |  |  | 0.01 |  |
| Manual | 328 | 44% |  | 561 | 38% |  |  |  |
| Non-manual | 411 | 56% |  | 898 | 62% |  |  |  |
| Education | 789 |  |  | 1459 |  |  | <0.001 |  |
| Degree | 113 | 14% |  | 279 | 19% |  |  |  |
| Higher education to GCE A level or equivalent | 186 | 24% |  | 360 | 25% |  |  |  |
| GCSE Grades A-E or equivalent | 282 | 36% |  | 528 | 36% |  |  |  |
| No qualifications or other qualifications | 176 | 26% |  | 292 | 20% |  |  |  |
| Ethnicity | 789 |  |  | 1459 |  |  | <0.001 |  |
| Non-white | 70 | 9% |  | 70 | 5% |  |  |  |
| White | 719 | 91% |  | 1389 | 95% |  |  |  |
| * Participants with available data in the respective variable but excluded from our analysis because of missing data in other covariates.  ** Pearson chi-square (for categorical variables) and Mann-Whitney (for continuous variables) p-values.  *** Excluding supplements and alcohol  Abbreviations: BMI, Body Mass Index; TEI, Total Energy Intake; EER, Estimated Energy Requirements; MVPA, Mild to Vigorous Physical Activity; GCE, General Certificate of Education; GCSE, General Certificate of Secondary Education | | | | | | | | |

| **Table s4.** Sensitivity analyses for Emotional eating and frequency (n=1459), size (n=1459) and timing (n=1437). | | | | | | | | | | | |  |
| --- | --- | --- | --- | --- | --- | --- | --- | --- | --- | --- | --- | --- |
|  | **Fully adjusted*** | |  | **Fully adjusted* without BMI** | |  | **Fully adjusted* without Misreporting category** | |  | **Fully adjusted* without External eating** | | |
| **Aspect of eating architecture** | **Β** | **(95% CI)** |  | **Β** | **(95% CI)** |  | **B** | **(95% CI)** |  | **B** | **(95% CI)** | |
| Frequency of eating |  |  |  |  |  |  |  |  |  |  |  | |
| Number of  eating occasions/week | 1.7 | (0, 3.4) |  | 1.2 | (-0.5, 2.9) |  | 1.5 | (-0.2, 3.2) |  | 1.6 | (0.1, 3.2) | |
| Number of meals/week | 0.1 | (-0.3, 0.4) |  | 0 | (-0.4, 0.3) |  | 0.0 | (-0.3, 0.4) |  | 0.1 | (-0.2, 0.4) | |
| Number of snacks/week | 1.4 | (0.5, 2.3) |  | 1.2 | (0.4, 2.1) |  | 1.4 | (0.5, 2.2) |  | 1.3 | (0.5, 2) | |
| Number of drinks/week | 0.2 | (-1.2, 1.6) |  | 0 | (-1.4, 1.4) |  | 0.0 | (-1.4, 1.4) |  | 0.2 | (-1.1, 1.4) | |
| Size of eating occasions |  |  |  |  |  |  |  |  |  |  |  | |
| Kcal per  eating occasion | -6 | (-12, 1) |  | -3 | (-10, 3) |  | -5 | (-12, 1) |  | -5 | (-11, 1) | |
| Kcal per meal | -15 | (-26, -3) |  | -10 | (-21, 1) |  | -14 | (-26, -3) |  | -15 | (-25, -5) | |
| Kcal per snack | -2 | (-14, 9) |  | -2 | (-13, 9) |  | -2 | (-13, 9) |  | -1 | (-11, 9) | |
| Kcal per drink | -1 | (-5, 2) |  | 0 | (-4, 3) |  | -2 | (-5, 2) |  | -1 | (-4, 2) | |
| Eating period (min) |  |  |  |  |  |  |  |  |  |  |  | |
| Eating occasions | 10 | (0, 20) |  | 7 | (-3, 17) |  | 9 | (-1, 19) |  | 12 | (3, 21) | |
| Meals | 5 | (-9, 18) |  | 3 | (-10, 16) |  | 4 | (-9, 18) |  | 6 | (-6, 18) | |
| Snacks | 35 | (16, 53) |  | 32 | (14, 50) |  | 34 | (16, 53) |  | 26 | (10, 42) | |
| Drinks | 5 | (-18, 27) |  | -3 | (-26, 19) |  | 3 | (-19, 26) |  | 8 | (-12, 29) | |
| Time of first eating occasion (clock time) |  |  |  |  |  |  |  |  |  |  |  | |
| Eating occasions | -5 | (-13, 2) |  | -4 | (-11, 4) |  | -5 | (-13, 2) |  | -6 | (-13, 0) | |
| Meals | -4 | (-17, 8) |  | -2 | (-15, 10) |  | -4 | (-17, 8) |  | -4 | (-15, 7) | |
| Snacks | -18 | (-34, -3) |  | -18 | (-33, -2) |  | -18 | (-34, -2) |  | -14 | (-27, 0) | |
| Drinks | -4 | (-22, 14) |  | 2 | (-16, 19) |  | -4 | (-22, 14) |  | -4 | (-20, 12) | |
| Time of last eating occasion (clock time) |  |  |  |  |  |  |  |  |  |  |  | |
| Eating occasions | 4 | (-2, 11) |  | 4 | (-2, 10) |  | 4 | (-2, 10) |  | 6 | (0, 11) | |
| Meals | 0 | (-7, 8) |  | 1 | (-7, 8) |  | 0 | (-7, 8) |  | 2 | (-5, 9) | |
| Snacks | 16 | (0, 33) |  | 14 | (-2, 31) |  | 16 | (0, 33) |  | 12 | (-2, 27) | |
| Drinks | 0 | (-14, 15) |  | -2 | (-16, 13) |  | -1 | (-15, 13) |  | 4 | (-8, 17) | |
| *Most adjusted includes: Age, Sex, Ethnicity (White, non-white), Occupational social class (manual, non-manual), Educational attainment (Highest educational qualification: Degree or equivalent, Higher education or GCE A level equivalent, GCSE grades A-E or equivalent, No qualifications or other qualifications), Sleep duration (short, average, long), Smoking (Currently a smoker, Past smoker, Never a smoker), Restrained eating, External Eating, Energy intake (Average over 7 days of energy intake, excluding supplements and alcohol (kcal)), Physical activity (Average minutes spent daily on at least moderate activity), Currently on a diet to lose weight (Yes, No), Eating affected by being unwell (Yes, No), BMI, Misreporting category (underreporting, normal reporting, overreporting) | | | | | | | | | | | |  |

| **Table s5.** Sensitivity analyses for External eating and frequency (n=1459), size (n=1459) and timing (n=1437). | | | | | | | | | | | |  |
| --- | --- | --- | --- | --- | --- | --- | --- | --- | --- | --- | --- | --- |
|  | **Fully adjusted*** | |  | **Fully adjusted* without BMI** | |  | **Fully adjusted* without Misreporting category** | |  | **Fully adjusted* without Emotional eating** | | |
| **Aspect of eating architecture** | **Β** | **(95% CI)** |  | **Β** | **(95% CI)** |  | **B** | **(95% CI)** |  | **B** | **(95% CI)** | |
| **Frequency** |  |  |  |  |  |  |  |  |  |  |  | |
| Number of  eating occasions/week | -0.1 | (-2.2, 2) |  | 0 | (-2.1, 2.1) |  | 0 | (-2.1, 2.2) |  | 0.9 | (-1, 2.7) | |
| Number of meals/week | 0.1 | (-0.3, 0.5) |  | 0.1 | (-0.3, 0.5) |  | 0.1 | (-0.3, 0.5) |  | 0.1 | (-0.3, 0.5) | |
| Number of snacks/week | -0.3 | (-1.4, 0.7) |  | -0.3 | (-1.4, 0.8) |  | -0.3 | (-1.4, 0.8) |  | 0.5 | (-0.5, 1.4) | |
| Number of drinks/week | 0 | (-1.7, 1.7) |  | 0 | (-1.7, 1.8) |  | 0.1 | (-1.6, 1.8) |  | 0.1 | (-1.4, 1.6) | |
| **Size** |  |  |  |  |  |  |  |  |  |  |  | |
| Kcal per  eating occasion | 2 | (-7, 10) |  | 1 | (-7, 10) |  | 2 | (-7, 10) |  | -1 | (-9, 6) | |
| Kcal per meal | 0 | (-14, 14) |  | -1 | (-15, 13) |  | 0 | (-14, 14) |  | -8 | (-21, 4) | |
| Kcal per snack | 3 | (-11, 17) |  | 3 | (-11, 17) |  | 2 | (-12, 16) |  | 2 | (-11, 14) | |
| Kcal per drink | 0 | (-4, 4) |  | 0 | (-5, 4) |  | 1 | (-4, 5) |  | -1 | (-4, 3) | |
| **Eating period (min)** |  |  |  |  |  |  |  |  |  |  |  | |
| Eating occasions | 6 | (-7, 18) |  | 6 | (-6, 19) |  | 7 | (-6, 19) |  | 11 | (0, 22) | |
| Meals | 3 | (-14, 20) |  | 3 | (-13, 20) |  | 4 | (-13, 20) |  | 6 | (-9, 20) | |
| Snacks | -24 | (-46, -1) |  | -23 | (-45, 0) |  | -23 | (-46, -1) |  | -4 | (-24, 16) | |
| Drinks | 10 | (-18, 38) |  | 12 | (-17, 40) |  | 11 | (-17, 39) |  | 13 | (-12, 38) | |
| Time of first eating occasion (clock time) |  |  |  |  |  |  |  |  |  |  |  | |
| Eating occasions | -2 | (-12, 7) |  | -3 | (-12, 7) |  | -3 | (-12, 7) |  | -5 | (-14, 3) | |
| Meals | 1 | (-14, 17) |  | 1 | (-15, 16) |  | 1 | (-15, 16) |  | -1 | (-15, 13) | |
| Snacks | 12 | (-7, 31) |  | 12 | (-7, 31) |  | 12 | (-7, 32) |  | 2 | (-15, 19) | |
| Drinks | 0 | (-22, 22) |  | -1 | (-23, 21) |  | 0 | (-22, 22) |  | -2 | (-22, 17) | |
| Time of last eating occasion (clock time) |  |  |  |  |  |  |  |  |  |  |  | |
| Eating occasions | 3 | (-4, 11) |  | 4 | (-4, 11) |  | 4 | (-4, 12) |  | 6 | (-1, 13) | |
| Meals | 4 | (-5, 14) |  | 4 | (-5, 14) |  | 5 | (-5, 14) |  | 5 | (-4, 13) | |
| Snacks | -11 | (-32, 9) |  | -11 | (-31, 10) |  | -11 | (-31, 10) |  | -2 | (-20, 16) | |
| Drinks | 10 | (-8, 28) |  | 10 | (-7, 28) |  | 11 | (-7, 29) |  | 10 | (-5, 26) | |
| *Most adjusted includes: Age, Sex, Ethnicity (White, non-white), Occupational social class (manual, non-manual), Educational attainment (Highest educational qualification: Degree or equivalent, Higher education or GCE A level equivalent, GCSE grades A-E or equivalent, No qualifications or other qualifications), Sleep duration (short, average, long), Smoking (Currently a smoker, Past smoker, Never a smoker), Restrained eating, External Eating, Energy intake (Average over 7 days of energy intake, excluding supplements and alcohol (kcal)), Physical activity (Average minutes spent daily on at least moderate activity), Currently on a diet to lose weight (Yes, No), Eating affected by being unwell (Yes, No), BMI, Misreporting category (underreporting, normal reporting, overreporting) | | | | | | | | | | | |  |

| **Table s6.** Effect measure modification by Restrained eating status (n=1437 for timing, n=1459 for size and frequency) | | | | | | | | | | | | | | | | | | | | | |
| --- | --- | --- | --- | --- | --- | --- | --- | --- | --- | --- | --- | --- | --- | --- | --- | --- | --- | --- | --- | --- | --- |
|  | **Emotional eating** | | | | | | | |  |  |  | **External eating** | | | | | | | |  |  |
|  | **All** | |  | **Unrestrained eaters** | |  | **Restrained  eaters** | |  | **P** |  | **All** | |  | **Unrestrained eaters** | |  | **Restrained eaters** | |  | **P** |
| **Eating architecture  aspect** |  | |  |  | |  |  | |  |  |  |  | |  |  | |  |  | |  |  |
|  | **Β** | **(95% CI)** |  | **B** | **(95% CI)** |  | **Β** | **(95% CI)** |  |  |  | **B** | **(95% CI)** |  | **B** | **(95% CI)** |  | **B** | **(95% CI)** |  |  |
| **Frequency** |  |  |  |  |  |  |  |  |  |  |  |  |  |  |  |  |  |  |  |  |  |
| Number of eating occasions/week | 1.7 | (0, 3.4) |  | 1.2 | (-0.7, 3.1) |  | 3 | (0.2, 5.8) |  | 0.256 |  | -0.1 | (-2.2, 2) |  | -0.1 | (-2.4, 2.1) |  | -0.1 | (-4.2, 4) |  | 0.998 |
| Number of meals/week | 0.1 | (-0.3, 0.4) |  | 0.1 | (-0.3, 0.5) |  | -0.2 | (-0.7, 0.4) |  | 0.337 |  | 0.1 | (-0.3, 0.5) |  | 0.3 | (-0.2, 0.7) |  | -0.7 | (-1.5, 0.2) |  | 0.038 |
| Number of snacks / week | 1.4 | (0.5, 2.3) |  | 1.4 | (0.4, 2.4) |  | 1.3 | (-0.2, 2.8) |  | 0.896 |  | -0.3 | (-1.4, 0.7) |  | -0.4 | (-1.6, 0.7) |  | 0.1 | (-2, 2.2) |  | 0.613 |
| Number of drinks/week | 0.2 | (-1.2, 1.6) |  | -0.3 | (-1.8, 1.3) |  | 1.4 | (-0.9, 3.7) |  | 0.188 |  | 0 | (-1.7, 1.7) |  | 0 | (-1.8, 1.8) |  | 0.1 | (-3.2, 3.5) |  | 0.937 |
| **Size** |  |  |  |  |  |  |  |  |  |  |  |  |  |  |  |  |  |  |  |  |  |
| Kcal per eating occasion | -6 | (-12, 1) |  | -4 | (-11, 4) |  | -11 | (-22, 1) |  | 0.252 |  | 2 | (-7, 10) |  | 2 | (-7, 11) |  | -1 | (-18, 15) |  | 0.667 |
| Kcal per meal | -15 | (-26, -3) |  | -16 | (-28, -3) |  | -11 | (-30, 7) |  | 0.65 |  | 0 | (-14, 14) |  | -6 | (-20, 9) |  | 25 | (-2, 52) |  | 0.032 |
| Kcal per snack | -2 | (-14, 9) |  | -5 | (-17, 8) |  | 4 | (-15, 23) |  | 0.383 |  | 3 | (-11, 17) |  | 1 | (-14, 15) |  | 13 | (-14, 41) |  | 0.374 |
| Kcal per drink | -1 | (-5, 2) |  | -3 | (-7, 1) |  | 3 | (-3, 9) |  | 0.059 |  | 0 | (-4, 4) |  | -1 | (-6, 3) |  | 5 | (-3, 14) |  | 0.159 |
| **Period** |  |  |  |  |  |  |  |  |  |  |  |  |  |  |  |  |  |  |  |  |  |
| Eating occasions | 10 | (0, 20) |  | 8 | (-3, 19) |  | 15 | (-2, 32) |  | 0.425 |  | 6 | (-7, 18) |  | 6 | (-8, 19) |  | 7 | (-17, 31) |  | 0.936 |
| Meals | 5 | (-9, 18) |  | 6 | (-9, 21) |  | 2 | (-21, 24) |  | 0.744 |  | 3 | (-14, 20) |  | 7 | (-11, 24) |  | -13 | (-45, 19) |  | 0.248 |
| Snacks | 35 | (16, 53) |  | 31 | (11, 51) |  | 45 | (14, 75) |  | 0.425 |  | -24 | (-46, -1) |  | -26 | (-50, -1) |  | -14 | (-58, 30) |  | 0.623 |
| Drinks | 5 | (-18, 27) |  | 1 | (-24, 26) |  | 15 | (-23, 53) |  | 0.515 |  | 10 | (-18, 38) |  | 11 | (-19, 41) |  | 5 | (-49, 60) |  | 0.849 |
| **Time of first occasion** |  |  |  |  |  |  |  |  |  |  |  |  |  |  |  |  |  |  |  |  |  |
| Eating occasions | -5 | (-13, 2) |  | -5 | (-14, 3) |  | -5 | (-18, 7) |  | 0.968 |  | -2 | (-12, 7) |  | -3 | (-13, 7) |  | 1 | (-17, 19) |  | 0.654 |
| Meals | -4 | (-17, 8) |  | -5 | (-19, 9) |  | -1 | (-22, 20) |  | 0.738 |  | 1 | (-14, 17) |  | -3 | (-19, 14) |  | 20 | (-10, 50) |  | 0.151 |
| Snacks | -18 | (-34, -3) |  | -18 | (-35, 0) |  | -20 | (-46, 6) |  | 0.851 |  | 12 | (-7, 31) |  | 16 | (-5, 36) |  | -3 | (-40, 35) |  | 0.366 |
| Drinks | -4 | (-22, 14) |  | -3 | (-23, 17) |  | -8 | (-38, 22) |  | 0.779 |  | 0 | (-22, 22) |  | -3 | (-26, 21) |  | 11 | (-32, 54) |  | 0.547 |
| **Time of last occasion** |  |  |  |  |  |  |  |  |  |  |  |  |  |  |  |  |  |  |  |  |  |
| Eating occasions | 4 | (-2, 11) |  | 2 | (-4, 9) |  | 10 | (0, 20) |  | 0.179 |  | 3 | (-4, 11) |  | 2 | (-6, 11) |  | 8 | (-7, 23) |  | 0.501 |
| Meals | 0 | (-7, 8) |  | 0 | (-8, 9) |  | 0 | (-12, 13) |  | 0.981 |  | 4 | (-5, 14) |  | 4 | (-6, 14) |  | 7 | (-11, 25) |  | 0.739 |
| Snacks | 16 | (0, 33) |  | 14 | (-5, 32) |  | 24 | (-3, 52) |  | 0.482 |  | -11 | (-32, 9) |  | -10 | (-32, 12) |  | -17 | (-56, 23) |  | 0.757 |
| Drinks | 0 | (-14, 15) |  | -2 | (-18, 14) |  | 7 | (-17, 31) |  | 0.491 |  | 10 | (-8, 28) |  | 8 | (-10, 27) |  | 17 | (-18, 51) |  | 0.651 |
| Adjusted for: Age, Sex, Ethnicity (White, non-white), Occupational social class (manual, non-manual), Educational attainment (Highest educational qualification: Degree or equivalent, Higher education or GCE A level equivalent, GCSE grades A-E or equivalent, No qualifications or other qualifications), Sleep duration (short, average, long), Smoking (Currently a smoker, Past smoker, Never a smoker), Restrained eating, External Eating, Energy intake (Average over 7 days of energy intake, excluding supplements and alcohol (kcal)), Physical activity (Average minutes spent daily on at least moderate activity), Currently on a diet to lose weight (Yes, No), Eating affected by being unwell (Yes, No), BMI, Misreporting category (underreporting, normal reporting, overreporting) | | | | | | | | | | | | | | | | | | | | | |

| **Table s7.** Effect measure modification by Sex (n=1437 for timing, n=1459 for size and frequency). | | | | | | | | | | | | | | | | | | | | | |
| --- | --- | --- | --- | --- | --- | --- | --- | --- | --- | --- | --- | --- | --- | --- | --- | --- | --- | --- | --- | --- | --- |
|  | **Emotional eating** | | | | | | | |  |  |  | **External eating** | | | | | | | |  |  |
|  | **All** | |  | **Men** | |  | **Women** | |  | **P** |  | **All** | |  | **Men** | |  | **Women** | |  | **P** |
| **Eating architecture  aspect** |  | |  |  | |  |  | |  |  |  |  | |  |  | |  |  | |  |  |
|  | **Β** | **(95% CI)** |  | **B** | **(95% CI)** |  | **Β** | **(95% CI)** |  |  |  | **B** | **(95% CI)** |  | **B** | **(95% CI)** |  | **B** | **(95% CI)** |  |  |
| **Frequency** |  |  |  |  |  |  |  |  |  |  |  |  |  |  |  |  |  |  |  |  |  |
| Number of eating occasions/week | 1.7 | (0, 3.4) |  | 0.8 | (-1.8, 3.4) |  | 2.1 | (0.2, 4.1) |  | 0.385 |  | -0.1 | (-2.2, 2) |  | -0.7 | (-3.3, 1.9) |  | 0.7 | (-2.2, 3.5) |  | 0.435 |
| Number of meals/week | 0.1 | (-0.3, 0.4) |  | 0 | (-0.6, 0.5) |  | 0.1 | (-0.3, 0.5) |  | 0.724 |  | 0.1 | (-0.3, 0.5) |  | 0.1 | (-0.4, 0.6) |  | 0.1 | (-0.5, 0.7) |  | 0.927 |
| Number of snacks / week | 1.4 | (0.5, 2.3) |  | 0.7 | (-0.7, 2) |  | 1.7 | (0.7, 2.7) |  | 0.179 |  | -0.3 | (-1.4, 0.7) |  | -0.7 | (-2, 0.6) |  | 0.2 | (-1.3, 1.6) |  | 0.340 |
| Number of drinks/week | 0.2 | (-1.2, 1.6) |  | 0.3 | (-1.8, 2.5) |  | 0.1 | (-1.5, 1.7) |  | 0.855 |  | 0 | (-1.7, 1.7) |  | -0.2 | (-2.3, 1.9) |  | 0.3 | (-2.1, 2.6) |  | 0.735 |
| **Size** |  |  |  |  |  |  |  |  |  |  |  |  |  |  |  |  |  |  |  |  |  |
| Kcal per eating occasion | -6 | (-12, 1) |  | -4 | (-14, 7) |  | -6 | (-14, 1) |  | 0.682 |  | 2 | (-7, 10) |  | 4 | (-6, 14) |  | -1 | (-13, 10) |  | 0.441 |
| Kcal per meal | -15 | (-26, -3) |  | -10 | (-27, 8) |  | -17 | (-30, -4) |  | 0.476 |  | 0 | (-14, 14) |  | -1 | (-18, 16) |  | 1 | (-18, 19) |  | 0.920 |
| Kcal per snack | -2 | (-14, 9) |  | 4 | (-14, 21) |  | -5 | (-18, 8) |  | 0.356 |  | 3 | (-11, 17) |  | 8 | (-9, 25) |  | -4 | (-23, 15) |  | 0.290 |
| Kcal per drink | -1 | (-5, 2) |  | 1 | (-4, 7) |  | -2 | (-6, 2) |  | 0.222 |  | 0 | (-4, 4) |  | 1 | (-5, 6) |  | -1 | (-7, 5) |  | 0.699 |
| **Period** |  |  |  |  |  |  |  |  |  |  |  |  |  |  |  |  |  |  |  |  |  |
| Eating occasions | 10 | (0, 20) |  | 1 | (-15, 16) |  | 14 | (3, 25) |  | 0.138 |  | 6 | (-7, 18) |  | 4 | (-11, 20) |  | 8 | (-9, 24) |  | 0.766 |
| Meals | 5 | (-9, 18) |  | -2 | (-23, 19) |  | 8 | (-8, 23) |  | 0.401 |  | 3 | (-14, 20) |  | 5 | (-16, 25) |  | 1 | (-22, 23) |  | 0.758 |
| Snacks | 35 | (16, 53) |  | 27 | (-1, 55) |  | 38 | (17, 59) |  | 0.479 |  | -24 | (-46, -1) |  | -27 | (-54, 1) |  | -20 | (-50, 11) |  | 0.709 |
| Drinks | 5 | (-18, 27) |  | 15 | (-21, 50) |  | 0 | (-26, 26) |  | 0.464 |  | 10 | (-18, 38) |  | 23 | (-12, 57) |  | -6 | (-45, 32) |  | 0.214 |
| **Time of first occasion** |  |  |  |  |  |  |  |  |  |  |  |  |  |  |  |  |  |  |  |  |  |
| Eating occasions | -5 | (-13, 2) |  | -2 | (-13, 10) |  | -7 | (-16, 2) |  | 0.420 |  | -2 | (-12, 7) |  | -3 | (-15, 8) |  | -1 | (-14, 11) |  | 0.773 |
| Meals | -4 | (-17, 8) |  | 3 | (-16, 23) |  | -8 | (-22, 7) |  | 0.314 |  | 1 | (-14, 17) |  | -5 | (-24, 14) |  | 10 | (-11, 31) |  | 0.252 |
| Snacks | -18 | (-34, -3) |  | -14 | (-38, 10) |  | -20 | (-38, -2) |  | 0.664 |  | 12 | (-7, 31) |  | 19 | (-5, 42) |  | 4 | (-22, 30) |  | 0.358 |
| Drinks | -4 | (-22, 14) |  | -20 | (-48, 8) |  | 3 | (-17, 23) |  | 0.145 |  | 0 | (-22, 22) |  | -10 | (-37, 17) |  | 13 | (-18, 43) |  | 0.222 |
| **Time of last occasion** |  |  |  |  |  |  |  |  |  |  |  |  |  |  |  |  |  |  |  |  |  |
| Eating occasions | 4 | (-2, 11) |  | -1 | (-10, 9) |  | 7 | (0, 14) |  | 0.152 |  | 3 | (-4, 11) |  | 1 | (-8, 11) |  | 6 | (-4, 17) |  | 0.405 |
| Meals | 0 | (-7, 8) |  | 1 | (-11, 13) |  | 0 | (-9, 9) |  | 0.860 |  | 4 | (-5, 14) |  | 0 | (-12, 11) |  | 10 | (-3, 23) |  | 0.180 |
| Snacks | 16 | (0, 33) |  | 13 | (-13, 38) |  | 18 | (-1, 37) |  | 0.710 |  | -11 | (-32, 9) |  | -8 | (-33, 17) |  | -16 | (-43, 12) |  | 0.649 |
| Drinks | 0 | (-14, 15) |  | -5 | (-27, 17) |  | 3 | (-13, 19) |  | 0.509 |  | 10 | (-8, 28) |  | 13 | (-9, 35) |  | 6 | (-18, 30) |  | 0.653 |
| Adjusted for: Age, Sex, Ethnicity (White, non-white), Occupational social class (manual, non-manual), Educational attainment (Highest educational qualification: Degree or equivalent, Higher education or GCE A level equivalent, GCSE grades A-E or equivalent, No qualifications or other qualifications), Sleep duration (short, average, long), Smoking (Currently a smoker, Past smoker, Never a smoker), Restrained eating, External Eating, Energy intake (Average over 7 days of energy intake, excluding supplements and alcohol (kcal)), Physical activity (Average minutes spent daily on at least moderate activity), Currently on a diet to lose weight (Yes, No), Eating affected by being unwell (Yes, No), BMI, Misreporting category (underreporting, normal reporting, overreporting) | | | | | | | | | | | | | | | | | | | | | |

| Table s8. All models for emotional eating (Size and frequency, n=1459, Timing n=1437) | | | | | | | | | | | |
| --- | --- | --- | --- | --- | --- | --- | --- | --- | --- | --- | --- |
|  | ***Frequency of eating occasions*** | | | | | | | | | | |
| Model | **Number of eating occasions per week** | |  | **Number of meals per week** | |  | **Number of snacks per week** | |  | **Number of drinks per week** | |
|  | **Β**  **(95% CI)** | |  | **B** | **(95% CI)** |  | **B** | **(95% CI)** |  | **B** | **(95% CI)** |
| Emotional | 1.7 (0.2, 3.2) | |  | 0.1 (-0.3, 0.4) | |  | 1 (0.3, 1.7) | |  | 1 (0.3, 1.7) | |
| Emotional, Sex | 2.4 (0.8, 3.9) | |  | 0.4 (0, 0.7) | |  | 1.2 (0.4, 1.9) | |  | 1.2 (0.4, 1.9) | |
| Emotional, Age and Sex | 3.1 (1.5, 4.7) | |  | 0.6 (0.3, 1) | |  | 1.5 (0.7, 2.2) | |  | 1.5 (0.7, 2.2) | |
| Most adjusted* | 1.7 (0, 3.4) | |  | 0.1 (-0.3, 0.4) | |  | 1.4 (0.5, 2.3) | |  | 0.2 (-1.2, 1.6) | |
| Sensitivity (BMI) | 1.2 (-0.5, 2.9) | |  | 0 (-0.4, 0.3) | |  | 1.2 (0.4, 2.1) | |  | 0 (-1.4, 1.4) | |
| Sensitivity 2 (Plausibility) | 1.5 (-0.2, 3.2) | |  | 0 (-0.3, 0.4) | |  | 1.4 (0.5, 2.2) | |  | 0 (-1.4, 1.4) | |
| Sensitivity 3 (External) | 1.6 (0.1, 3.2) | |  | 0.1 (-0.2, 0.4) | |  | 1.3 (0.5, 2) | |  | 0.2 (-1.1, 1.4) | |
|  | ***Size of eating occasions*** | | | | | | | | | | |
|  | **Kcal per eating occasion** | |  | **Kcal per meal** | |  | **Kcal per snack** | |  | **Kcal per drink** | |
|  | Β  **(95% CI)** | |  | **B**  **(95% CI)** | |  | **B**  **(95% CI)** | |  | **B**  **(95% CI)** | |
| Emotional | -16 (-22, -10) | |  | -37 (-49, -26) | |  | -8 (-17, 2) | |  | -7 (-10, -4) | |
| Emotional, Sex | -1 (-7, 6) | |  | -2 (-13, 8) | |  | 2 (-7, 12) | |  | -1 (-4, 2) | |
| Emotional, Age and Sex | -3 (-9, 3) | |  | -9 (-20, 2) | |  | -3 (-13, 6) | |  | -3 (-6, 0) | |
| Most adjusted* | -6 (-12, 1) | |  | -15 (-26, -3) | |  | -2 (-14, 9) | |  | -1 (-5, 2) | |
| Sensitivity (BMI) | -3 (-10, 3) | |  | -10 (-21, 1) | |  | -2 (-13, 9) | |  | 0 (-4, 3) | |
| Sensitivity 2 (Plausibility) | -5 (-12, 1) | |  | -14 (-26, -3) | |  | -2 (-13, 9) | |  | -2 (-5, 2) | |
| Sensitivity 3 (External) | -5 (-11, 1) | |  | -15 (-25, -5) | |  | -1 (-11, 9) | |  | -1 (-4, 2) | |
|  | ***Eating period*** | | | | | | | | | | |
|  | **Eating occasions** | |  | **Meals** | |  | **Snacks** | |  | **Drinks** | |
|  | **Β** | **(95% CI)** |  | **Β** | **(95% CI)** |  | **Β** | **(95% CI)** |  | **Β** | **(95% CI)** |
| Emotional | 5 (-4, 14) | |  | 4 (-9, 17) | |  | 18 (4, 33) | |  | 13 (-6, 32) | |
| Emotional, Sex | 8 (-1, 18) | |  | 14 (0, 27) | |  | 23 (8, 38) | |  | 9 (-11, 28) | |
| Emotional, Age and Sex | 16 (7, 25) | |  | 25 (11, 38) | |  | 27 (12, 43) | |  | 12 (-7, 32) | |
| Most adjusted* | 10 (0, 20) | |  | 5 (-9, 18) | |  | 35 (16, 53) | |  | 5 (-18, 27) | |
| Sensitivity (BMI) | 7 (-3, 17) | |  | 3 (-10, 16) | |  | 32 (14, 50) | |  | -3 (-26, 19) | |
| Sensitivity 2 (Plausibility) | 9 (-1, 19) | |  | 4 (-9, 18) | |  | 34 (16, 53) | |  | 3 (-19, 26) | |
| Sensitivity 3 (External) | 12 (3, 21) | |  | 6 (-6, 18) | |  | 26 (10, 42) | |  | 8 (-12, 29) | |
|  | ***First time of eating*** | | | | | | | | | | |
|  | **Eating occasions** | |  | **Meals** | |  | **Snacks** | |  | **Drinks** | |
|  | **Β** | **(95% CI)** |  | **Β** | **(95% CI)** |  | **Β** | **(95% CI)** |  | **Β** | **(95% CI)** |
| Emotional | 0 (-6, 7) | |  | -5 (-16, 7) | |  | -5 (-17, 7) | |  | -4 (-19, 10) | |
| Emotional, Sex | -2 (-8, 5) | |  | -8 (-20, 4) | |  | -8 (-20, 5) | |  | 1 (-14, 16) | |
| Emotional, Age and Sex | -7 (-14, -1) | |  | -18 (-30, -6) | |  | -10 (-23, 3) | |  | -3 (-18, 13) | |
| Most adjusted* | -5 (-13, 2) | |  | -4 (-17, 8) | |  | -18 (-34, -3) | |  | -4 (-22, 14) | |
| Sensitivity (BMI) | -4 (-11, 4) | |  | -2 (-15, 10) | |  | -18 (-33, -2) | |  | 2 (-16, 19) | |
| Sensitivity 2 (Plausibility) | -5 (-13, 2) | |  | -4 (-17, 8) | |  | -18 (-34, -2) | |  | -4 (-22, 14) | |
| Sensitivity 3 (External) | -6 (-13, 0) | |  | -4 (-15, 7) | |  | -14 (-27, 0) | |  | -4 (-20, 12) | |
|  | ***Last time of eating*** | | | | | | | | | | |
|  | **Eating occasions** | |  | **Meals** | |  | **Snacks** | |  | **Drinks** | |
|  | **Β** | **(95% CI)** |  | **Β** | **(95% CI)** |  | **Β** | **(95% CI)** |  | **Β** | **(95% CI)** |
| Emotional | 5 (0, 10) | |  | 0 (-7, 6) | |  | 13 (0, 27) | |  | 9 (-3, 20) | |
| Emotional, Sex | 7 (1, 12) | |  | 6 (-1, 12) | |  | 15 (1, 29) | |  | 9 (-3, 22) | |
| Emotional, Age and Sex | 8 (3, 14) | |  | 7 (0, 14) | |  | 17 (3, 31) | |  | 10 (-3, 22) | |
| Most adjusted* | 4 (-2, 11) | |  | 0 (-7, 8) | |  | 16 (0, 33) | |  | 0 (-14, 15) | |
| Sensitivity (BMI) | 4 (-2, 10) | |  | 1 (-7, 8) | |  | 14 (-2, 31) | |  | -2 (-16, 13) | |
| Sensitivity 2 (Plausibility) | 4 (-2, 10) | |  | 0 (-7, 8) | |  | 16 (0, 33) | |  | -1 (-15, 13) | |
| Sensitivity 3 (External) | 6 (0, 11) | |  | 2 (-5, 9) | |  | 12 (-2, 27) | |  | 4 (-8, 17) | |
| *Most adjusted includes: Age, Sex, Ethnicity (White, non-white), Occupational social class (manual, non-manual), Educational attainment (Highest educational qualification: Degree or equivalent, Higher education or GCE A level equivalent, GCSE grades A-E or equivalent, No qualifications or other qualifications), Sleep duration (short, average, long), Smoking (Currently a smoker, Past smoker, Never a smoker), Restrained eating (High, Low), External Eating, Energy intake (Average over 7 days of energy intake, excluding supplements and alcohol (kcal)), Physical activity (Average minutes spent daily on at least moderate activity), Currently on a diet to lose weight (Yes, No), Eating affected by being unwell (Yes, No), BMI, Misreporting category (underreporting, normal reporting, overreporting) | | | | | | | | | | | |

| Table s9. All models for external eating (Size and frequency, n=1459, Timing n=1437) | | | | | | | | | | | |
| --- | --- | --- | --- | --- | --- | --- | --- | --- | --- | --- | --- |
|  | ***Frequency of eating occasions*** | | | | | | | | | | |
| Model | **Number of eating occasions per week** | |  | **Number of meals per week** | |  | **Number of snacks per week** | |  | **Number of drinks per week** | |
|  | **Β** | **(95% CI)** |  | **B** | **(95% CI)** |  | **B** | **(95% CI)** |  | **B** | **(95% CI)** |
| External | 3.1 (1.2, 5.1) | |  | 0.3 (-0.1, 0.8) | |  | 0.9 (0, 1.8) | |  | 1.8 (0.4, 3.3) | |
| External, Sex | 3.2 (1.2, 5.1) | |  | 0.3 (-0.1, 0.8) | |  | 0.9 (0, 1.8) | |  | 1.9 (0.4, 3.3) | |
| External, Age and Sex | 4.9 (3, 6.9) | |  | 1 (0.5, 1.4) | |  | 1.6 (0.6, 2.5) | |  | 1.9 (0.3, 3.4) | |
| Most adjusted* | -0.1 (-2.2, 2) | |  | 0.1 (-0.3, 0.5) | |  | -0.3 (-1.4, 0.7) | |  | 0 (-1.7, 1.7) | |
| Sensitivity (BMI) | 0 (-2.1, 2.1) | |  | 0.1 (-0.3, 0.5) | |  | -0.3 (-1.4, 0.8) | |  | 0 (-1.7, 1.8) | |
| Sensitivity 2 (Plausibility) | 0 (-2.1, 2.2) | |  | 0.1 (-0.3, 0.5) | |  | -0.3 (-1.4, 0.8) | |  | 0.1 (-1.6, 1.8) | |
| Sensitivity 3 (Emotional) | 0.9 (-1, 2.7) | |  | 0.1 (-0.3, 0.5) | |  | 0.5 (-0.5, 1.4) | |  | 0.1 (-1.4, 1.6) | |
|  | ***Size of eating occasions*** | | | | | | | | | | |
|  | **Kcal per eating occasion** | |  | **Kcal per meal** | |  | **Kcal per snack** | |  | **Kcal per drink** | |
|  | Β | **(95% CI)** |  | **B** | **(95% CI)** |  | **B** | **(95% CI)** |  | **B** | **(95% CI)** |
| External | 5 (-3, 13) | |  | 17 (3, 32) | |  | 14 (2, 26) | |  | 2 (-2, 5) | |
| External, Sex | 6 (-2, 13) | |  | 19 (6, 32) | |  | 15 (3, 26) | |  | 2 (-2, 6) | |
| External, Age and Sex | 1 (-7, 9) | |  | 6 (-7, 20) | |  | 3 (-9, 15) | |  | -2 (-5, 2) | |
| Most adjusted* | 2 (-7, 10) | |  | 0 (-14, 14) | |  | 3 (-11, 17) | |  | 0 (-4, 4) | |
| Sensitivity (BMI) | 1 (-7, 10) | |  | -1 (-15, 13) | |  | 3 (-11, 17) | |  | 0 (-5, 4) | |
| Sensitivity (Plausibility) | 2 (-7, 10) | |  | 0 (-14, 14) | |  | 2 (-12, 16) | |  | 1 (-4, 5) | |
| Sensitivity 3 (Emotional) | -1 (-9, 6) | |  | -8 (-21, 4) | |  | 2 (-11, 14) | |  | -1 (-4, 3) | |
|  | ***Eating period*** | | | | | | | | | | |
|  | **Eating occasions** | |  | **Meals** | |  | **Snacks** | |  | **Drinks** | |
|  | **Β** | **(95% CI)** |  | **Β** | **(95% CI)** |  | **Β** | **(95% CI)** |  | **Β** | **(95% CI)** |
| External | 13 (1, 24) | |  | 11 (-6, 27) | |  | 2 (-16, 21) | |  | 28 (4, 52) | |
| External, Sex | 13 (1, 24) | |  | 11 (-5, 28) | |  | 3 (-16, 21) | |  | 28 (4, 52) | |
| External, Age and Sex | 30 (18, 41) | |  | 36 (20, 53) | |  | 11 (-8, 31) | |  | 38 (13, 63) | |
| Most adjusted* | 6 (-7, 18) | |  | 3 (-14, 20) | |  | -24 (-46, -1) | |  | 10 (-18, 38) | |
| Sensitivity (BMI) | 6 (-6, 19) | |  | 3 (-13, 20) | |  | -23 (-45, 0) | |  | 12 (-17, 40) | |
| Sensitivity 2 (Plausibility) | 7 (-6, 19) | |  | 4 (-13, 20) | |  | -23 (-46, -1) | |  | 11 (-17, 39) | |
| Sensitivity 3 (Emotional) | 11 (0, 22) | |  | 6 (-9, 20) | |  | -4 (-24, 16) | |  | 13 (-12, 38) | |
|  | ***First time of eating*** | | | | | | | | | | |
|  | **Eating occasions** | |  | **Meals** | |  | **Snacks** | |  | **Drinks** | |
|  | **Β** | **(95% CI)** |  | **Β** | **(95% CI)** |  | **Β** | **(95% CI)** |  | **Β** | **(95% CI)** |
| External | -2 (-10, 6) | |  | -1 (-16, 14) | |  | 8 (-8, 23) | |  | -7 (-25, 12) | |
| External, Sex | -2 (-10, 6) | |  | -1 (-16, 13) | |  | 8 (-8, 23) | |  | -7 (-25, 12) | |
| External, Age and Sex | -15 (-23, -7) | |  | -23 (-38, -8) | |  | 3 (-14, 19) | |  | -15 (-34, 4) | |
| Most adjusted* | -2 (-12, 7) | |  | 1 (-14, 17) | |  | 12 (-7, 31) | |  | 0 (-22, 22) | |
| Sensitivity (BMI) | -3 (-12, 7) | |  | 1 (-15, 16) | |  | 12 (-7, 31) | |  | -1 (-23, 21) | |
| Sensitivity 2 (Plausibility) | -3 (-12, 7) | |  | 1 (-15, 16) | |  | 12 (-7, 32) | |  | 0 (-22, 22) | |
| Sensitivity 3 (Emotional) | -5 (-14, 3) | |  | -1 (-15, 13) | |  | 2 (-15, 19) | |  | -2 (-22, 17) | |
|  | ***Last time of eating*** | | | | | | | | | | |
|  | **Eating occasions** | |  | **Meals** | |  | **Snacks** | |  | **Drinks** | |
|  | **Β** | **(95% CI)** |  | **Β** | **(95% CI)** |  | **Β** | **(95% CI)** |  | **Β** | **(95% CI)** |
| External | 11 (4, 17) | |  | 10 (2, 18) | |  | 10 (-7, 27) | |  | 21 (7, 36) | |
| External, Sex | 11 (4, 17) | |  | 10 (2, 18) | |  | 10 (-7, 27) | |  | 21 (6, 36) | |
| External, Age and Sex | 15 (8, 22) | |  | 14 (5, 22) | |  | 14 (-4, 32) | |  | 23 (8, 38) | |
| Most adjusted* | 3 (-4, 11) | |  | 4 (-5, 14) | |  | -11 (-32, 9) | |  | 10 (-8, 28) | |
| Sensitivity (BMI) | 4 (-4, 11) | |  | 4 (-5, 14) | |  | -11 (-31, 10) | |  | 10 (-7, 28) | |
| Sensitivity 2 (Plausibility) | 4 (-4, 12) | |  | 5 (-5, 14) | |  | -11 (-31, 10) | |  | 11 (-7, 29) | |
| Sensitivity 3 (Emotional) | 6 (-1, 13) | |  | 5 (-4, 13) | |  | -2 (-20, 16) | |  | 10 (-5, 26) | |
| *Most adjusted includes: Age, Sex, Ethnicity (White, non-white), Occupational social class (manual, non-manual), Educational attainment (Highest educational qualification: Degree or equivalent, Higher education or GCE A level equivalent, GCSE grades A-E or equivalent, No qualifications or other qualifications), Sleep duration (short, average, long), Smoking (Currently a smoker, Past smoker, Never a smoker), Restrained eating (High, Low), Emotional Eating, Energy intake (Average over 7 days of energy intake, excluding supplements and alcohol (kcal)), Physical activity (Average minutes spent daily on at least moderate activity), Currently on a diet to lose weight (Yes, No), Eating affected by being unwell (Yes, No), BMI, Misreporting category (underreporting, normal reporting, overreporting) | | | | | | | | | | | |

| **Table s10.** Correlations between Eating Styles in the NDNS (2000-2001), according to inclusion in analysis. | | | | | | | |
| --- | --- | --- | --- | --- | --- | --- | --- |
|  | **Excluded* (n=360)** | | |  | **Included (n=1459)** | | |
|  | **Emotional** | **External** | **Restrained** |  | **Emotional** | **External** | **Restrained** |
| **Emotional** | 1** |  |  |  | 1 |  |  |
| **External** | 0.42 | 1 |  |  | 0.50 | 1 |  |
| **Restrained** | 0.33 | 0.19 | 1 |  | 0.33 | 0.19 | 1 |
|  | **Excluded* (n=382)** | | |  | **Included (n=1437)** | | |
| **Emotional** | 1 |  |  |  | 1 |  |  |
| **External** | 0.42 | 1 |  |  | 0.50 | 1 |  |
| **Restrained** | 0.33 | 0.20 | 1 |  | 0.33 | 0.18 | 1 |
| * Participants with available data in the respective variable but excluded from our analysis because of missing data in other covariates of the most adjusted models.  ** Pearson correlation coefficient | | | | | | | |

# Eating architecture syntax

SPSS syntax used to generate eating architecture data in the UK adult NDNS in 2000.

* Encoding: UTF-8.

SET UNICODE OFF.

***Read in food item file and sort for merging with container file data.

IMPORT

FILE='N:\NDNS pre 2000\NDNS 19-64 yrs 2000-2001\UKDA-5140-spss\spss\container.por' .

SORT CASES BY caseid dayno cntnrno.

SAVE OUTFILE='N:\NDNS pre 2000\NDNS 19-64 yrs 2000-2001\TEMP.sav'

/COMPRESSED.

**Read in container file, sort and then merge with food item file data.

IMPORT

FILE='N:\NDNS pre 2000\NDNS 19-64 yrs 2000-2001\UKDA-5140-spss\spss\fooditem.por' .

SORT CASES BY caseid dayno cntnrno.

STAR JOIN

/SELECT t0.area, t0.address, t0.checkl, t0.itemno, t0.homegrow, t0.wtserved, t0.leftind,

t0.estimate, t0.foodcode, t0.brand, t0.spillage, t0.fdname, t0.wteaten, t0.nutf01, t0.nutf02,

t0.nutf03, t0.nutf04, t0.nutf05, t0.nutf06, t0.nutf07, t0.nutf08, t0.nutf09, t0.nutf10, t0.nutf11,

t0.nutf12, t0.nutf13, t0.nutf14, t0.nutf15, t0.nutf16, t0.nutf17, t0.nutf18, t0.nutf19, t0.nutf20,

t0.nutf21, t0.nutf22, t0.nutf23, t0.nutf24, t0.nutf25, t0.nutf26, t0.nutf27, t0.nutf28, t0.nutf29,

t0.nutf30, t0.nutf31, t0.nutf32, t0.nutf33, t0.nutf34, t0.nutf35, t0.nutf36, t0.nutf37, t0.nutf38,

t0.nutf39, t0.nutf40, t0.nutf41, t0.nutf42, t0.nutf43, t0.nutf44, t0.nutf45, t0.nutf46, t0.nutf47,

t0.nutf48, t0.nutf49, t0.nutf50, t0.nutf51, t0.nutf52, t0.nutf53, t0.nutf54, t0.nutf55,

t0.foodgrpc, t0.dilute, t0.startdat, t0.dvhsize, t0.dvilores, t0.dvilohoh, t0.dvilohih, t0.dietary,

t0.hhtype1, t0.hhtype2, t0.regsumm, t0.waveint, t0.respsex, t0.respage, t0.respmar, t0.respwith,

t0.resphldr, t0.dvincgp, t0.dvrecben, t0.dveducgp, t0.dvhrpsc3, t0.ragegp, t0.intwgt, t0.scresp,

t0.schoh, t0.schrp, t0.resphoh, t0.resphrp, t0.ownhome, t0.fcredit, t0.isupp, t0.iseek, t0.gincome,

t0.rspdvmar, t0.vegi, t0.ethnic, t0.agebp, t0.ageht, t0.agewt, t0.agehip, t0.agebld, t0.ageur,

t0.ageintd, t0.agediet, t1.mealtime, t1.whereeat, t1.weighby, t1.cntnrwt, t1.totleft, t1.foodsrce,

t1.wtleft

/FROM * AS t0

/JOIN 'N:\NDNS pre 2000\NDNS 19-64 yrs 2000-2001\TEMP.sav' AS t1

ON t0.caseid=t1.caseid

AND t0.dayno=t1.dayno AND t0.cntnrno=t1.cntnrno

/OUTFILE FILE='N:\NDNS pre 2000\NDNS 19-64 yrs 2000-2001\FooditemMealtime.sav' .

***Convert stored data in integers to real numbers page 755 explanation and 777 Figure 3.13 for conversion factors**.

GET

FILE='N:\NDNS pre 2000\NDNS 19-64 yrs 2000-2001\FooditemMealtime.sav'.

Compute Grams_item = wteaten/10 .

VAR LAB Grams_item 'Gram intake of food item'.

Compute EI_Kcal_item = nutf05/10000 .

VAR LAB EI_Kcal_item 'Energy intake (Kcal) of food item'.

Compute EI_kJ_item = nutf06/10000 .

VAR LAB EI_kJ_item 'Energy intake (kJ) food item'.

EXECUTE.

SORT CASES BY caseid.

***Label foods as meal foods, snack foods, drinks or supplements***.

If (foodgrpc=1)Meal_item=1.

If (foodgrpc=2)Meal_item=1.

If (foodgrpc=3)Meal_item=1.

If (foodgrpc=4)Meal_item=1.

If (foodgrpc=5)Meal_item=1.

If (foodgrpc=6)Meal_item=1.

If (foodgrpc=7)Meal_item=1.

If (foodgrpc=8)Meal_item=1.

If (foodgrpc=9)Meal_item=1.

If (foodgrpc=10)Meal_item=1.

If (foodgrpc=11)Meal_item=2.

If (foodgrpc=12)Meal_item=2.

If (foodgrpc=13)Meal_item=2.

If (foodgrpc=14)Meal_item=2.

If (foodgrpc=15)Meal_item=2.

If (foodgrpc=16)Meal_item=2.

If (foodgrpc=17)Meal_item=3.

If (foodgrpc=18)Meal_item=3.

If (foodgrpc=19)Meal_item=3.

If (foodgrpc=20)Meal_item=3.

If (foodgrpc=21)Meal_item=2.

If (foodgrpc=22)Meal_item=3.

If (foodgrpc=23)Meal_item=2.

If (foodgrpc=24)Meal_item=2.

If (foodgrpc=25)Meal_item=2.

If (foodgrpc=26)Meal_item=2.

If (foodgrpc=27)Meal_item=2.

If (foodgrpc=28)Meal_item=1.

If (foodgrpc=29)Meal_item=1.

If (foodgrpc=30)Meal_item=2.

If (foodgrpc=31)Meal_item=2.

If (foodgrpc=32)Meal_item=2.

If (foodgrpc=33)Meal_item=2.

If (foodgrpc=34)Meal_item=2.

If (foodgrpc=35)Meal_item=2.

If (foodgrpc=36)Meal_item=2.

If (foodgrpc=37)Meal_item=2.

If (foodgrpc=38)Meal_item=2.

If (foodgrpc=39)Meal_item=2.

If (foodgrpc=40)Meal_item=1.

If (foodgrpc=41)Meal_item=1.

If (foodgrpc=42)Meal_item=1.

If (foodgrpc=43)Meal_item=1.

If (foodgrpc=44)Meal_item=1.

If (foodgrpc=45)Meal_item=1.

If (foodgrpc=46)Meal_item=1.

If (foodgrpc=47)Meal_item=1.

If (foodgrpc=48)Meal_item=1.

If (foodgrpc=49)Meal_item=1.

If (foodgrpc=50)Meal_item=1.

If (foodgrpc=51)Meal_item=1.

If (foodgrpc=52)Meal_item=1.

If (foodgrpc=53)Meal_item=1.

If (foodgrpc=54)Meal_item=1.

If (foodgrpc=55)Meal_item=2.

If (foodgrpc=56)Meal_item=2.

If (foodgrpc=57)Meal_item=2.

If (foodgrpc=58)Meal_item=1.

If (foodgrpc=59)Meal_item=1.

If (foodgrpc=60)Meal_item=1.

If (foodgrpc=61)Meal_item=1.

If (foodgrpc=62)Meal_item=1.

If (foodgrpc=63)Meal_item=1.

If (foodgrpc=64)Meal_item=1.

If (foodgrpc=65)Meal_item=1.

If (foodgrpc=66)Meal_item=1.

If (foodgrpc=67)Meal_item=1.

If (foodgrpc=68)Meal_item=1.

If (foodgrpc=69)Meal_item=1.

If (foodgrpc=70)Meal_item=2.

If (foodgrpc=71)Meal_item=2.

If (foodgrpc=72)Meal_item=2.

If (foodgrpc=73)Meal_item=2.

If (foodgrpc=74)Meal_item=2.

If (foodgrpc=75)Meal_item=2.

If (foodgrpc=76)Meal_item=2.

If (foodgrpc=77)Meal_item=2.

If (foodgrpc=78)Meal_item=2.

If (foodgrpc=79)Meal_item=2.

If (foodgrpc=80)Meal_item=2.

If (foodgrpc=81)Meal_item=2.

If (foodgrpc=82)Meal_item=3.

If (foodgrpc=83)Meal_item=3.

If (foodgrpc=84)Meal_item=3.

If (foodgrpc=85)Meal_item=3.

If (foodgrpc=86)Meal_item=3.

If (foodgrpc=87)Meal_item=3.

If (foodgrpc=88)Meal_item=3.

If (foodgrpc=89)Meal_item=3.

If (foodgrpc=90)Meal_item=3.

If (foodgrpc=91)Meal_item=3.

If (foodgrpc=92)Meal_item=3.

If (foodgrpc=93)Meal_item=3.

If (foodgrpc=94)Meal_item=1.

If (foodgrpc=95)Meal_item=2.

If (foodgrpc=96)Meal_item=3.

If (foodgrpc=97)Meal_item=3.

If (foodgrpc=98)Meal_item=3.

If (foodgrpc=99)Meal_item=3.

If (foodgrpc=100)Meal_item=3.

If (foodgrpc=101)Meal_item=3.

If (foodgrpc=102)Meal_item=1.

If (foodgrpc=103)Meal_item=2.

If (foodgrpc=104)Meal_item=4.

If (foodgrpc=105)Meal_item=4.

If (foodgrpc=106)Meal_item=4.

If (foodgrpc=107)Meal_item=4.

If (foodgrpc=108)Meal_item=2.

If (foodgrpc=109)Meal_item=2.

If (foodgrpc=110)Meal_item=3.

If (foodgrpc=111)Meal_item=3.

If (foodgrpc=112)Meal_item=3.

If (foodgrpc=113)Meal_item=3.

If (foodgrpc=114)Meal_item=3.

If (foodgrpc=115)Meal_item=3.

VAR LAB Meal_item 'Food item is meal, snack, drink or supplement type'.

VAL LAB Meal_item 1 'Meal' 2 'Snack' 3 'Drink' 4 'Supplement' .

VAR LEV Meal_item (Nominal) .

***Add value labels to food group codes***.

VAL LAB foodgrpc 1 'pasta' 2 'rice' 3 'pizza' 4 'other cereal' 5 'whitebread' 6 'wholemealbread' 7 'softgrainbread' 8 'otherbread'

9 'whg&hfbfcereal' 10 'otherbfcereals' 11 'biscuits' 12 'fruitpies' 13 'bunscakespastries'14 'milkpuds' 15 'spongepuds'

16 'other' 17 'wholemilk' 18 'semi-skimmed' 19 'skimmedmilk' 20 'Infantformula' 21 'cream' 22 'othermilk' 23 'cottagechse'

24 'othercheese' 25 'fromagefrais' 26 'yogurt' 27 'othdairydessert' 28 'eggs' 29 'eggdishes' 30 'butter' 31 'pufamarge'

32 'pufaoils' 33 'pufalowfatsprd' 34 'lowfatspread' 35 'blockmarge' 36 'softmargenotpufa' 37 'oils&fatsnotpufa' 38 'pufareducfatsprd'

39 'reducedfatspread' 40 'bacon&ham' 41 'beefvealetc' 42 'lambetc' 43 'porketc' 44 'coatedchicken' 45 'chckn&turkey' 46 'liveretc'

47 'burgerskebab' 48 'sausages' 49 'meatpiesetc' 50 'othermeat' 51 'friedwhitefish' 52 'othwhitefish' 53 'shellfish' 54 'oilyfish'

55 'rawcarrots' 56 'othersalad' 57 'rawtomatoes' 58 'peas' 59 'greenbeans' 60 'bakedbeans' 61 'leafygreen' 62 'carrot' 63 'cookedtoms'

64 'vegetabledishes' 65 'otherveg' 66 'potatochips' 67 'otherfriedpots' 68 'nonfriedpotprods' 69 'otherpotatodishes' 70 'applespears'

71 'orangesetc' 72 'bananas' 73 'fruitinjuice' 74 'fruitinsyrup' 75 'otherfruit' 76 'sugar' 77 'preserves' 78 'othersugars' 79 'savourysnacks'

80 'sugarconfect' 81 'chocolatecon' 82 'fruitjuice' 83 'liqueurs' 84 'spirits' 85 'wine' 86 'fortifiedwn' 87 'lowalcwine' 88 'beers'

89 'lowalcbeers' 90 'ciderperry' 91 'lowalcciderperry' 92 'alco-pops' 93 'beverage' 94 'soups' 95 'savourysauces' 96 'coffee'

97 'tea' 98 'herbaltea' 99 'bottledwater' 100 'tapwater' 101 'commtoddlerdrinks' 102 'commtoddlerfood' 103 'icecream' 104 'vitamintab'

105 'vitaminoil' 106 'vitamindrop' 107 'nutrcompletesuppl' 108 'sweeteners' 109 'nuts&seeds' 110 'softdrinkconcnd' 111 'softdrinkco2nd'

112 'softdrinkstillnd' 113 'softdrinkconcdt' 114 'softdrinkco2dt' 115 'softdrinkstilldt'.

EXECUTE.

SAVE OUTFILE='N:\NDNS pre 2000\NDNS 19-64 yrs 2000-2001\FooditemMealtime.sav'

/DROP=nutf01 nutf02 nutf03 nutf04 nutf05 nutf06 nutf07 nutf08 nutf09 nutf10 nutf11 nutf12 nutf13 nutf14 nutf15 nutf16

nutf17 nutf18 nutf19 nutf20 nutf21 nutf22 nutf23 nutf24 nutf25 nutf26 nutf27 nutf28 nutf29 nutf30 nutf31 nutf32 nutf33 nutf34

nutf35 nutf36 nutf37 nutf38 nutf39 nutf40 nutf41 nutf42 nutf43 nutf44 nutf45 nutf46 nutf47 nutf48 nutf49 nutf50 nutf51 nutf52

nutf53 nutf54 nutf55

/COMPRESSED.

***Aggregate data on food items eaten at the same mealtime***.

GET

FILE='N:\NDNS pre 2000\NDNS 19-64 yrs 2000-2001\FooditemMealtime.sav' .

SORT CASES BY caseid(A) dayno(A) mealtime(A).

AGGREGATE

/OUTFILE='N:\NDNS pre 2000\NDNS 19-64 yrs 2000-2001\Period0.sav'

/PRESORTED

/BREAK=caseid dayno mealtime

/Grams 'Amount eaten (g) per occasion'=SUM(Grams_item)

/Energy_kcal 'Energy intake (kcal) per occasion'=SUM(EI_Kcal_item)

/Energy_kJ 'Energy intake (kJ) per occasion'=SUM(EI_kJ_item)

/p_meal 'Percent of food items in occasion that are meal foods'=PIN(Meal_item,0.5,1.5)

/p_snack 'Percent of food items in occasion that are snack foods'=PIN(Meal_item,1.5,2.5)

/p_drink 'Percent of food items in occasion that are drinks'=PIN(Meal_item,2.5,3.5)

/p_supp 'Percent of food items in occasion that are supplements'=PIN(Meal_item,3.5,4.5)

/Time 'Time of eating occasion'=MEDIAN(mealtime)

/Min_food 'Lowest food group included in eating occasion'=MIN(foodgrpc)

/Med_food 'Median food group included in eating occasion'=MEDIAN(foodgrpc)

/Max_food 'Highest food group included in eating occasion'=MAX(foodgrpc)

/n_items 'Total number of items in eating occasion'=N.

GET

FILE='N:\NDNS pre 2000\NDNS 19-64 yrs 2000-2001\Period0.sav' .

SORT CASES BY caseid(A) dayno(A) mealtime(A).

***Convert time data into separate hour and minute variables to compute time in minutes since midnight, to be able to compute total eating period and intermeal intervals using subtraction (and for meaningful description on a continuous decimal scale).

COMPUTE MealHour=TRUNC(mealtime/100).

COMPUTE MealMinute=mealtime-(MealHour*100).

COMPUTE mealtime_minutes=(MealHour*60)+MealMinute.

VAR LAB mealtime_minutes 'Time of eating occasion in minutes since midnight'.

***Aggregate timing of eating occasions in the same day***.

AGGREGATE

/OUTFILE='N:\NDNS pre 2000\NDNS 19-64 yrs 2000-2001\aggr.sav'

/PRESORTED

/BREAK=caseid dayno

/TimeMed 'Time of median eating occasion (minutes since midnight)'=MEDIAN(mealtime_minutes)

/TimeFirst 'Time of first eating occasion (minutes since midnight)'=FIRST(mealtime_minutes)

/TimeLast 'Time of last eating occasion (minutes since midnight)'=LAST(mealtime_minutes)

/n_occasions 'Total number of eating occasions (based on unique times)'=N.

GET

FILE='N:\NDNS pre 2000\NDNS 19-64 yrs 2000-2001\aggr.sav' .

***Estimate eating period by subtracting the first time of eating from the last time of eating, the is the total time duration over which eating occurs in a day.

COMPUTE EatingPeriod=TimeLast-TimeFirst.

VAR LAB EatingPeriod 'Time duration (minutes) over which eating occasion occur in a day)'.

***Estimate intermeal intervals by dividing total eating period by the number of eating occasions. This is OK if inter-meal intervals are not highly variable (see later code to check this).

COMPUTE InterMealInterval=EatingPeriod/n_occasions.

VAR LAB InterMealInterval 'Estimated daily average intermeal interval (minutes) from EatingPeriod/n_occasions'.

EXECUTE .

SORT CASES BY caseid(A).

***Aggregate timing and frequency of eating occasions over 7 days in the same person***.

AGGREGATE

/OUTFILE='N:\NDNS pre 2000\NDNS 19-64 yrs 2000-2001\TEMP.sav'

/PRESORTED

/BREAK=caseid

/TimeFirst_median 'First time of eating (Median over 7 days minutes since midnight)'=MEDIAN(TimeFirst)

/TimeFirst_mean 'First time of eating (Mean over 7 days minutes since midnight)'=MEAN(TimeFirst)

/TimeFirst_SD 'First time of eating (SD over 7 days minutes since midnight)'=SD(TimeFirst)

/TimeFirst_min 'First time of eating (Min over 7 days minutes since midnight)'=MIN(TimeFirst)

/TimeFirst_max 'First time of eating (Max over 7 days minutes since midnight)'=MAX(TimeFirst)

/TimeLast_median 'Last time of eating (Median over 7 days minutes since midnight)'=MEDIAN(TimeLast)

/TimeLast_mean 'Last time of eating (Mean over 7 days minutes since midnight)'=MEAN(TimeLast)

/TimeLast_SD 'Last time of eating (SD over 7 days minutes since midnight)'=SD(TimeLast)

/TimeLast_min 'Last time of eating (Min over 7 days minutes since midnight)'=MIN(TimeLast)

/TimeLast_max 'Last time of eating (Max over 7 days minutes since midnight)'=MAX(TimeLast)

/EatingPeriod_mean 'Mean eating period (minutes) over 7 days'=MEAN(EatingPeriod)

/EatingPeriod_SD 'SD eating period (minutes) over 7 days'=SD(EatingPeriod)

/EatingPeriod_min 'Minimum eating period (minutes) over 7 days'=MIN(EatingPeriod)

/EatingPeriod_max 'Maximum eating period (minutes) over 7 days'=MAX(EatingPeriod)

/InterMealInterval_mean 'Mean (across 7 days) of estimated daily average intermeal interval (minutes) from EatingPeriod/n_occasions'=MEAN(InterMealInterval)

/n_occasions_mean 'Mean number of eating occasions (times per day) over 7 days'=MEAN(n_occasions)

/n_occasions_median 'Median number of eating occasions (times per day) over 7 days'=MEDIAN(n_occasions)

/n_occasions_SD 'SD of number of eating occasions (times per day) over 7 days'=SD(n_occasions)

/n_occasions_min 'Minimum of number of eating occasions (times per day) over 7 days'=MIN(n_occasions)

/n_occasions_max 'Maximum of number of eating occasions (times per day) over 7 days'=MAX(n_occasions)

/n_days 'Total number of days'=N.

GET

FILE='N:\NDNS pre 2000\NDNS 19-64 yrs 2000-2001\TEMP.sav' .

**Compute the range of eating occasion frequency over 7 days.

COMPUTE n_occasion_range=n_occasions_max - n_occasions_min.

VAR LAB n_occasion_range 'Range of number of eating occasions (times per day) over 7 days'.

COMPUTE TimeFirst_range=TimeFirst_max - TimeFirst_min.

VAR LAB TimeFirst_range 'Range of first time of eating occasions (minutes since midnight) over 7 days'.

COMPUTE TimeLast_range=TimeLast_max - TimeLast_min.

VAR LAB TimeLast_range 'Range of Last time of eating occasions (minutes since midnight) over 7 days'.

COMPUTE EatingPeriod_range=EatingPeriod_max - EatingPeriod_min.

VAR LAB EatingPeriod_range 'Range of eating period (minutes) over 7 days'.

exe .

SORT CASES BY caseid(A) .

**Save person level file containing eating architecture variables, merge all subsequent EA variables created into this file.

SAVE OUTFILE='N:\NDNS pre 2000\NDNS 19-64 yrs 2000-2001\Eating Architecture.sav'

/COMPRESSED.

GET

FILE='N:\NDNS pre 2000\NDNS 19-64 yrs 2000-2001\Period0.sav' .

SORT CASES BY caseid(A) dayno(A) mealtime(A).

***Convert time data into separate hour and minute variables to compute time in minutes since midnight, to be able to compute total eating period and intermeal intervals using subtraction (and for meaningful description on a continuous decimal scale).

COMPUTE MealHour=TRUNC(mealtime/100).

COMPUTE MealMinute=mealtime-(MealHour*100).

COMPUTE mealtime_minutes=(MealHour*60)+MealMinute.

exe.

***Transpose the long (line per eating occasion) file into a wide day level file. This is so that different eating occasion in each day are stored in separate variables and not on separate line.

***Enables exact time difference between eating occasion to be computed rather than estimated from EatingPeriod/n_occasions- this enables variation in IMI to be characterised.

CASESTOVARS

/ID=caseid dayno

/GROUPBY=VARIABLE

/COUNT=occasion "Total number of eating occasions (in each day)".

***In this dataset the max number of eating occasion in a day that any person reports is 31, therfore 30 time difference variables are computed.

***Not all people have values for each time difference, it depends on the total number of eating occasion they had on each day.

***Some people only have one eating occasion in a day therefore no time differences can be computed.

COMPUTE timediff1 = mealtime_minutes.2 - mealtime_minutes.1 .

COMPUTE timediff2 = mealtime_minutes.3 - mealtime_minutes.2 .

COMPUTE timediff3 = mealtime_minutes.4 - mealtime_minutes.3 .

COMPUTE timediff4 = mealtime_minutes.5 - mealtime_minutes.4 .

COMPUTE timediff5 = mealtime_minutes.6 - mealtime_minutes.5 .

COMPUTE timediff6 = mealtime_minutes.7 - mealtime_minutes.6 .

COMPUTE timediff7 = mealtime_minutes.8 - mealtime_minutes.7 .

COMPUTE timediff8 = mealtime_minutes.9 - mealtime_minutes.8 .

COMPUTE timediff9 = mealtime_minutes.10 - mealtime_minutes.9 .

COMPUTE timediff10 = mealtime_minutes.11 - mealtime_minutes.10 .

COMPUTE timediff11 = mealtime_minutes.12 - mealtime_minutes.11 .

COMPUTE timediff12 = mealtime_minutes.13 - mealtime_minutes.12 .

COMPUTE timediff13 = mealtime_minutes.14 - mealtime_minutes.13 .

COMPUTE timediff14 = mealtime_minutes.15 - mealtime_minutes.14 .

COMPUTE timediff15 = mealtime_minutes.16 - mealtime_minutes.15 .

COMPUTE timediff16 = mealtime_minutes.17 - mealtime_minutes.16 .

COMPUTE timediff17 = mealtime_minutes.18 - mealtime_minutes.17 .

COMPUTE timediff18 = mealtime_minutes.19 - mealtime_minutes.18 .

COMPUTE timediff19 = mealtime_minutes.20 - mealtime_minutes.19 .

COMPUTE timediff20 = mealtime_minutes.21 - mealtime_minutes.20 .

COMPUTE timediff21 = mealtime_minutes.22 - mealtime_minutes.21 .

COMPUTE timediff22 = mealtime_minutes.23 - mealtime_minutes.22 .

COMPUTE timediff23 = mealtime_minutes.24 - mealtime_minutes.23 .

COMPUTE timediff24 = mealtime_minutes.25 - mealtime_minutes.24 .

COMPUTE timediff25 = mealtime_minutes.26 - mealtime_minutes.25 .

COMPUTE timediff26 = mealtime_minutes.27 - mealtime_minutes.26 .

COMPUTE timediff27 = mealtime_minutes.28 - mealtime_minutes.27 .

COMPUTE timediff28 = mealtime_minutes.29 - mealtime_minutes.28 .

COMPUTE timediff29 = mealtime_minutes.30 - mealtime_minutes.29 .

COMPUTE timediff30 = mealtime_minutes.31 - mealtime_minutes.30 .

COMPUTE timediff_mean=MEAN(timediff1,timediff2,timediff3,timediff4,timediff5,timediff6,timediff7,timediff8,timediff9,timediff10,

timediff11,timediff12,timediff13,timediff14,timediff15,timediff16,timediff17,timediff18,timediff19,timediff20,timediff21,timediff22,timediff23,

timediff24,timediff25,timediff26,timediff27,timediff28,timediff29,timediff30).

EXECUTE.

SORT CASES BY caseid(A) dayno(A).

***Aggregate the time differences across 7 days within a person - this enables variation in IMI to be characterised.

AGGREGATE

/OUTFILE='N:\NDNS pre 2000\NDNS 19-64 yrs 2000-2001\TEMP.sav'

/PRESORTED

/BREAK=caseid

/timediff_mean_7days 'Mean inter-meal interval (minutes) directly computed average over 7 days'=MEAN(timediff_mean)

/timediff_median_7days 'Median inter-meal interval (minutes) directly computed average over 7 days'=MEDIAN(timediff_mean)

/timediff_SD_7days 'SD inter-meal interval (minutes) directly computed average over 7 days'=SD(timediff_mean)

/timediff_min_7days 'Min inter-meal interval (minutes) directly computed average over 7 days'=MIN(timediff_mean)

/timediff_max_7days 'Max inter-meal interval (minutes) directly computed average over 7 days'=MAX(timediff_mean)

/n_days 'Total number of days'=N.

GET

FILE='N:\NDNS pre 2000\NDNS 19-64 yrs 2000-2001\TEMP.sav' .

SORT CASES BY caseid (A) .

MATCH FILES /FILE=*

/FILE='N:\NDNS pre 2000\NDNS 19-64 yrs 2000-2001\Eating Architecture.sav'

/BY caseid.

EXECUTE.

SAVE OUTFILE='N:\NDNS pre 2000\NDNS 19-64 yrs 2000-2001\Eating Architecture.sav'

/COMPRESSED.

********************************************************************************************************************************************************************************

Compute meal and snack size and frequency by unique times

********************************************************************************************************************************************************************************.

GET

FILE='N:\NDNS pre 2000\NDNS 19-64 yrs 2000-2001\period0.sav' .

***Convert time data into separate hour and minute variables to compute time in minutes since midnight, to be able to compute total eating period and intermeal intervals using subtraction (and for meaningful description on a continuous decimal scale).

COMPUTE MealHour=TRUNC(mealtime/100).

COMPUTE MealMinute=mealtime-(MealHour*100).

COMPUTE mealtime_minutes=(MealHour*60)+MealMinute.

VAR LAB mealtime_minutes 'Time of eating occasion in minutes since midnight'.

***Labelling eating occasions as meal or snack, first step to set all occasion to -1=unclassified**.

compute Occasion_Type=-1 .

**Criteria for a meal: If all items (100%) are meal items**.

IF (p_meal=100) Occasion_Type=1 .

FREQUENCIES VARIABLES=Occasion_Type /ORDER=ANALYSIS.

**Criteria for a snack: If 100% are snack items**.

IF (p_snack=100) Occasion_Type=2 .

FREQUENCIES VARIABLES=Occasion_Type /ORDER=ANALYSIS.

**Criteria for a drink: If 100% are drink items**.

IF (p_drink=100) Occasion_Type=3 .

FREQUENCIES VARIABLES=Occasion_Type /ORDER=ANALYSIS.

**Criteria for a supplement: If 100% are supplement items or if accompanied by a single snack item - 24 occasions, 16 of which are with sav sauces=medicine**.

IF (p_supp=100) Occasion_Type=4 .

FREQUENCIES VARIABLES=Occasion_Type /ORDER=ANALYSIS.

**Criteria for a meal: If 1 or more items (% depends on number of items) are meal items**.

IF (n_items=2 & p_meal>=50.0000 ) Occasion_Type=1 .

IF (n_items=3 & p_meal>=33.3333 ) Occasion_Type=1 .

IF (n_items=4 & p_meal>=25.0000 ) Occasion_Type=1 .

IF (n_items=5 & p_meal>=20.0000 ) Occasion_Type=1 .

IF (n_items=6 & p_meal>=16) Occasion_Type=1 .

IF (n_items=7 & p_meal>=14.2857 ) Occasion_Type=1 .

IF (n_items=8 & p_meal>=12.5000 ) Occasion_Type=1 .

IF (n_items=9 & p_meal>=11.1111 ) Occasion_Type=1 .

IF (n_items=10 & p_meal>=10.0000) Occasion_Type=1 .

IF (n_items=11 & p_meal>=9.0909 ) Occasion_Type=1 .

IF (n_items=12 & p_meal>=8.3333 ) Occasion_Type=1 .

IF (n_items=13 & p_meal>=7.6923 ) Occasion_Type=1 .

IF (n_items=14 & p_meal>=7) Occasion_Type=1 .

IF (n_items=15 & p_meal>=6) Occasion_Type=1 .

IF (n_items=16 & p_meal>=6.2500 ) Occasion_Type=1 .

IF (n_items=17 & p_meal>=5) Occasion_Type=1 .

IF (n_items=18 & p_meal>=5) Occasion_Type=1 .

IF (n_items=19 & p_meal>=5) Occasion_Type=1 .

IF (n_items=20 & p_meal>=5.0000 ) Occasion_Type=1 .

IF (n_items=21 & p_meal>=4.7619 ) Occasion_Type=1 .

IF (n_items=22 & p_meal>=4.5455 ) Occasion_Type=1 .

IF (n_items=23 & p_meal>=4.3478 ) Occasion_Type=1 .

IF (n_items=24 & p_meal>=4.1667 ) Occasion_Type=1 .

IF (n_items=25 & p_meal>=4.0000 ) Occasion_Type=1 .

IF (n_items=26 & p_meal>=3.8462 ) Occasion_Type=1 .

IF (n_items=27 & p_meal>=3.7037 ) Occasion_Type=1 .

IF (n_items=28 & p_meal>=3.571428571) Occasion_Type=1 .

IF (n_items=29 & p_meal>=3.448275862) Occasion_Type=1 .

IF (n_items=30 & p_meal>=3.333333333) Occasion_Type=1 .

IF (n_items=31 & p_meal>=3.225806452) Occasion_Type=1 .

IF (n_items=32 & p_meal>=3.125) Occasion_Type=1 .

FREQUENCIES VARIABLES=Occasion_Type /ORDER=ANALYSIS.

**Criteria for a snack: If there is just one meal item (ONLY e.g. soup on it's own or pizza on it's own) or one meal item with a snack item (77% bread and fat spread) or supp item**.

IF (n_items=1 & p_meal=100.0) Occasion_Type=2 .

FREQUENCIES VARIABLES=Occasion_Type /ORDER=ANALYSIS.

IF (n_items=2 & p_meal=50 & p_supp=50) Occasion_Type=2 .

FREQUENCIES VARIABLES=Occasion_Type /ORDER=ANALYSIS.

IF (n_items=2 & p_meal=50 & p_snack=50) Occasion_Type=2 .

FREQUENCIES VARIABLES=Occasion_Type /ORDER=ANALYSIS.

**Criteria for a drink: In a 2 item eating occasion if 50%=snack and 50%=drink on 60% of occasions this is medicine with water; then tea with sugar; coffee with cream**.

IF (n_items=2 & p_snack=50 & p_drink=50) Occasion_Type=3 .

FREQUENCIES VARIABLES=Occasion_Type /ORDER=ANALYSIS.

**Criteria for a drink: In a 2 item eating occasion if 50%=drink and 50%=supp this is vitamins with water**.

IF (n_items=2 & p_drink=50 & p_supp=50) Occasion_Type=3 .

FREQUENCIES VARIABLES=Occasion_Type /ORDER=ANALYSIS.

**Criteria for a supplement: If supplement items are accompanied by a snack item - most of which are with sav sauces=medicine**.

IF (n_items>1 & p_meal=0 & p_drink=0 & p_snack>0 & p_supp>0) Occasion_Type=4 .

FREQUENCIES VARIABLES=Occasion_Type /ORDER=ANALYSIS.

IF (n_items=3 & p_meal=0 & p_drink>0 & p_snack>0 & p_supp>0 & Min_food=95 & Med_food=100 & Max_food=104) Occasion_Type=4 .

FREQUENCIES VARIABLES=Occasion_Type /ORDER=ANALYSIS.

**Criteria for a snack with drink: If 0 items are meal items then eating occasion is a snack & drink (with or without a supp)**.

IF (n_items>2 & p_meal=0 & p_snack>0 & p_drink>0) Occasion_Type=5 .

FREQUENCIES VARIABLES=Occasion_Type /ORDER=ANALYSIS.

**Criteria for a drink: If 0 items are meal or snack then period is a drink (with or without a supp)**.

IF (n_items>2 & p_meal=0 & p_snack=0 & p_drink>0) Occasion_Type=3 .

FREQUENCIES VARIABLES=Occasion_Type /ORDER=ANALYSIS.

**Criteria for a drink: If period has 3 items including a snack and a drink (this could be tea or coffee with sugar or sweetener (with or without a supp))**.

IF (n_items=3 & p_meal=0 & p_drink>0 & p_snack>0 & (Min_food>16 & Min_food<22) & Med_food=76 & (Max_food>95 & Max_food<98)) Occasion_Type=3 .

FREQUENCIES VARIABLES=Occasion_Type

/ORDER=ANALYSIS.

IF (n_items=3 & p_meal=0 & p_drink>0 & p_snack>0 & (Min_food>16 & Min_food<22) & (Med_food>95 & Med_food<98) & Max_food=108) Occasion_Type=3 .

FREQUENCIES VARIABLES=Occasion_Type

/ORDER=ANALYSIS.

VAR LAB Occasion_Type 'Eating occasion is meal, snack, drink or supplement type based on food items eaten'.

VAL LAB Occasion_Type 1 'Meal' 2 'Snack' 3 'Drink' 4 'Supplement' 5 'Snack with drink' .

VAR LEV Occasion_Type (Nominal) .

EXECUTE.

SORT CASES BY Occasion_Type.

SPLIT FILE LAYERED BY Occasion_Type.

FREQUENCIES VARIABLES=Grams Energy_kcal Energy_kJ Time mealtime_minutes n_items Min_food Med_food Max_food p_meal p_snack p_drink p_supp

/FORMAT=NOTABLE

/NTILES=4

/STATISTICS=STDDEV MINIMUM MAXIMUM MEAN

/ORDER=ANALYSIS.

SORT CASES BY Occasion_Type.

**Combining Snacks with Snack+Drink for purpose of paper (revision May 2015)**.

RECODE Occasion_Type (5=2) .

EXECUTE.

SORT CASES BY Occasion_Type.

SPLIT FILE LAYERED BY Occasion_Type.

FREQUENCIES VARIABLES=Grams Energy_kcal Energy_kJ Time mealtime_minutes n_items Min_food Med_food Max_food p_meal p_snack p_drink p_supp

/FORMAT=NOTABLE

/NTILES=4

/STATISTICS=STDDEV MINIMUM MAXIMUM MEAN

/ORDER=ANALYSIS.

SORT CASES BY caseid dayno .

SAVE OUTFILE='N:\NDNS pre 2000\NDNS 19-64 yrs 2000-2001\period0.sav'

/COMPRESSED.

***Counting total number of MEAL eating occasions per day***.

USE ALL.

**Selects only eating occasion that are classified as meal periods .

SELECT IF (Occasion_Type = 1).

EXECUTE.

***Aggregate timing of eating occasions in the same day***.

AGGREGATE

/OUTFILE='N:\NDNS pre 2000\NDNS 19-64 yrs 2000-2001\aggr.sav'

/PRESORTED

/BREAK=caseid dayno

/TimeMed 'Time of median meal (minutes since midnight)'=MEDIAN(mealtime_minutes)

/TimeFirst 'Time of first meal (minutes since midnight)'=FIRST(mealtime_minutes)

/TimeLast 'Time of last meal (minutes since midnight)'=LAST(mealtime_minutes)

/n_occasions 'Total number of meals (based on unique times)'=N.

GET

FILE='N:\NDNS pre 2000\NDNS 19-64 yrs 2000-2001\aggr.sav' .

**Transposes the file from one line per day to one line per person with features of days as variables.

CASESTOVARS

/ID=caseid

/GROUPBY=VARIABLE

/COUNT=meal_days "Number of days meals eaten" .

***Replaces any missing values with 0 because these people did report eating on 7 days but simply had 0 meals on some days, this appears missing in the long file but should be 0.

RECODE n_occasions.1 n_occasions.2 n_occasions.3 n_occasions.4 n_occasions.5 n_occasions.6 n_occasions.7 (SYSMIS=0).

******************************************************************************************************************************************************************

***Adding in computation of eating period specifically for meals. Need to edit so that eating perio is generated for each day 1 to 7

***Then compute mean over 7 days, then replicate for snacks and drinks****

******************************************************************************************************************************************************************.

COMPUTE mealEatingPeriod1=TimeLast.1-TimeFirst.1.

VAR LAB mealEatingPeriod1 'Time duration (minutes) over which meal eating occasions occur on day 1)'.

COMPUTE mealEatingPeriod2=TimeLast.2-TimeFirst.2.

VAR LAB mealEatingPeriod2 'Time duration (minutes) over which meal eating occasions occur on day 2)'.

COMPUTE mealEatingPeriod3=TimeLast.3-TimeFirst.3.

VAR LAB mealEatingPeriod3 'Time duration (minutes) over which meal eating occasions occur on day 3)'.

COMPUTE mealEatingPeriod4=TimeLast.4-TimeFirst.4.

VAR LAB mealEatingPeriod4 'Time duration (minutes) over which meal eating occasions occur on day 4)'.

COMPUTE mealEatingPeriod5=TimeLast.5-TimeFirst.5.

VAR LAB mealEatingPeriod5 'Time duration (minutes) over which meal eating occasions occur on day 5)'.

COMPUTE mealEatingPeriod6=TimeLast.6-TimeFirst.6.

VAR LAB mealEatingPeriod6 'Time duration (minutes) over which meal eating occasions occur on day 6)'.

COMPUTE mealEatingPeriod7=TimeLast.7-TimeFirst.7.

VAR LAB mealEatingPeriod7 'Time duration (minutes) over which meal eating occasions occur on day 7)'.

COMPUTE mealEatingPeriod_mean=MEAN(mealEatingPeriod1,mealEatingPeriod2,mealEatingPeriod3,mealEatingPeriod4,mealEatingPeriod5,mealEatingPeriod6,mealEatingPeriod7) .

COMPUTE TimeFirstmeal_mean=MEAN(TimeFirst.1,TimeFirst.2,TimeFirst.3,TimeFirst.4,TimeFirst.5,TimeFirst.6,TimeFirst.7) .

COMPUTE TimeLastmeal_mean=MEAN(TimeLast.1,TimeLast.2,TimeLast.3,TimeLast.4,TimeLast.5,TimeLast.6,TimeLast.7).

VAR LAB mealEatingPeriod_mean 'Mean meal eating period (minutes, last-first time) over 7 days'.

VAR LAB TimeFirstmeal_mean 'Mean first meal time (minutes after midnight) over 7 days'.

VAR LAB TimeLastmeal_mean 'Mean last meal time (minutes after midnight) over 7 days'.

COMPUTE TimeFirstMeal_median=MEDIAN(TimeFirst.1,TimeFirst.2,TimeFirst.3,TimeFirst.4,TimeFirst.5,TimeFirst.6,TimeFirst.7) .

COMPUTE TimeFirstMeal_min=MIN(TimeFirst.1,TimeFirst.2,TimeFirst.3,TimeFirst.4,TimeFirst.5,TimeFirst.6,TimeFirst.7) .

COMPUTE TimeFirstMeal_max=MAX(TimeFirst.1,TimeFirst.2,TimeFirst.3,TimeFirst.4,TimeFirst.5,TimeFirst.6,TimeFirst.7) .

COMPUTE TimeLastMeal_median=MEDIAN(TimeLast.1,TimeLast.2,TimeLast.3,TimeLast.4,TimeLast.5,TimeLast.6,TimeLast.7).

COMPUTE TimeLastMeal_min=MIN(TimeLast.1,TimeLast.2,TimeLast.3,TimeLast.4,TimeLast.5,TimeLast.6,TimeLast.7).

COMPUTE TimeLastMeal_max=MAX(TimeLast.1,TimeLast.2,TimeLast.3,TimeLast.4,TimeLast.5,TimeLast.6,TimeLast.7).

COMPUTE n_Meal_mean=MEAN(n_occasions.1,n_occasions.2,n_occasions.3,n_occasions.4,n_occasions.5,n_occasions.6,n_occasions.7).

COMPUTE n_Meal_median=MEDIAN(n_occasions.1,n_occasions.2,n_occasions.3,n_occasions.4,n_occasions.5,n_occasions.6,n_occasions.7).

COMPUTE n_Meal_SD=SD(n_occasions.1,n_occasions.2,n_occasions.3,n_occasions.4,n_occasions.5,n_occasions.6,n_occasions.7).

COMPUTE n_Meal_min=MIN(n_occasions.1,n_occasions.2,n_occasions.3,n_occasions.4,n_occasions.5,n_occasions.6,n_occasions.7).

COMPUTE n_Meal_max=MAX(n_occasions.1,n_occasions.2,n_occasions.3,n_occasions.4,n_occasions.5,n_occasions.6,n_occasions.7).

COMPUTE n_Meal_range=n_Meal_max - n_Meal_min.

COMPUTE TimeFirstMeal_range=TimeFirstMeal_max - TimeFirstMeal_min.

COMPUTE TimeLastMeal_range=TimeLastMeal_max - TimeLastMeal_min.

VAR LAB n_Meal_range 'Range of number of meals (times per day) over 7 days'.

VAR LAB TimeFirstMeal_median 'Median first meal time (minutes after midnight) over 7 days'.

VAR LAB TimeFirstMeal_min 'Min first meal time (minutes after midnight) over 7 days'.

VAR LAB TimeFirstMeal_max 'Max first meal time (minutes after midnight) over 7 days'.

VAR LAB TimeFirstMeal_range 'Range first meal time (minutes) over 7 days'.

VAR LAB TimeLastMeal_median 'Median last meal time (minutes after midnight) over 7 days'.

VAR LAB TimeLastMeal_min 'Min last meal time (minutes after midnight) over 7 days'.

VAR LAB TimeLastMeal_max 'Max last meal time (minutes after midnight) over 7 days'.

VAR LAB TimeLastMeal_range 'Range last meal time (minutes) over 7 days'.

VAR LAB n_Meal_mean 'Mean meal frequency (times per day) over 7 days'.

VAR LAB n_Meal_median 'Median meal frequency (times per day) over 7 days'.

VAR LAB n_Meal_SD 'SD meal frequency (times per day) over 7 days'.

VAR LAB n_Meal_min 'Min meal frequency (times per day) over 7 days'.

VAR LAB n_Meal_max 'Max meal frequency (times per day) over 7 days'.

VAR LAB n_Meal_range 'Range meal frequency (times per day) over 7 days'.

EXECUTE.

SORT CASES BY caseid(A).

***Merge new variables into eating architecture variables file.

MATCH FILES /FILE=*

/FILE='N:\NDNS pre 2000\NDNS 19-64 yrs 2000-2001\Eating Architecture.sav'

/BY caseid.

EXECUTE.

SORT CASES BY caseid(A).

SAVE OUTFILE='N:\NDNS pre 2000\NDNS 19-64 yrs 2000-2001\Eating Architecture.sav'

/COMPRESSED.

***Compute the inter-meal interval for meal occasions only****.

GET

FILE='N:\NDNS pre 2000\NDNS 19-64 yrs 2000-2001\Period0.sav' .

***computing inter-MEAL interval for MEAL eating occasions per day***.

USE ALL.

**Selects only eating occasion that are classified as meal periods .

SELECT IF (Occasion_Type = 1).

EXECUTE.

SORT CASES BY caseid(A) dayno(A) mealtime(A).

***Convert time data into separate hour and minute variables to compute time in minutes since midnight, to be able to compute total eating period and intermeal intervals using subtraction (and for meaningful description on a continuous decimal scale).

COMPUTE MealHour=TRUNC(mealtime/100).

COMPUTE MealMinute=mealtime-(MealHour*100).

COMPUTE mealtime_minutes=(MealHour*60)+MealMinute.

exe.

***Transpose the long (line per eating occasion) file into a wide day level file. This is so that different eating occasion in each day are stored in separate variables and not on separate line.

***Enables exact time difference between eating occasion to be computed rather than estimated from EatingPeriod/n_occasions- this enables variation in IMI to be characterised.

CASESTOVARS

/ID=caseid dayno

/GROUPBY=VARIABLE

/COUNT=meal_occasion "Total number of MEAL eating occasions (in each day)".

***In this dataset the max number of meal eating occasion in a day that any person reports is 31, therfore 30 time difference variables are computed.

***Not all people have values for each time difference, it depends on the total number of eating occasion they had on each day.

***Some people only have one eating occasion in a day therefore no time differences can be computed.

COMPUTE mealtimediff1 = mealtime_minutes.2 - mealtime_minutes.1 .

COMPUTE mealtimediff2 = mealtime_minutes.3 - mealtime_minutes.2 .

COMPUTE mealtimediff3 = mealtime_minutes.4 - mealtime_minutes.3 .

COMPUTE mealtimediff4 = mealtime_minutes.5 - mealtime_minutes.4 .

COMPUTE mealtimediff5 = mealtime_minutes.6 - mealtime_minutes.5 .

COMPUTE mealtimediff6 = mealtime_minutes.7 - mealtime_minutes.6 .

COMPUTE mealtimediff7 = mealtime_minutes.8 - mealtime_minutes.7 .

COMPUTE mealtimediff8 = mealtime_minutes.9 - mealtime_minutes.8 .

COMPUTE mealtimediff9 = mealtime_minutes.10 - mealtime_minutes.9 .

COMPUTE mealtimediff_mean=MEAN(mealtimediff1,mealtimediff2,mealtimediff3,mealtimediff4,mealtimediff5,mealtimediff6,mealtimediff7,mealtimediff8,mealtimediff9).

EXECUTE.

SORT CASES BY caseid(A) dayno(A).

***Aggregate the time differences across 7 days within a person - this enables variation in IMI to be characterised.

AGGREGATE

/OUTFILE='N:\NDNS pre 2000\NDNS 19-64 yrs 2000-2001\TEMP.sav'

/PRESORTED

/BREAK=caseid

/mealtimediff_mean_7days 'Mean inter-meal interval (minutes) MEALS ONLY directly computed average over 7 days'=MEAN(mealtimediff_mean)

/mealtimediff_median_7days 'Median inter-meal interval (minutes) MEALS ONLY directly computed average over 7 days'=MEDIAN(mealtimediff_mean)

/mealtimediff_SD_7days 'SD inter-meal interval (minutes) directly MEALS ONLY computed average over 7 days'=SD(mealtimediff_mean)

/mealtimediff_min_7days 'Min inter-meal interval (minutes) MEALS ONLY directly computed average over 7 days'=MIN(mealtimediff_mean)

/mealtimediff_max_7days 'Max inter-meal interval (minutes) MEALS ONLY directly computed average over 7 days'=MAX(mealtimediff_mean)

/n_days 'Total number of days'=N.

GET

FILE='N:\NDNS pre 2000\NDNS 19-64 yrs 2000-2001\TEMP.sav' .

SORT CASES BY caseid (A) .

MATCH FILES /FILE=*

/FILE='N:\NDNS pre 2000\NDNS 19-64 yrs 2000-2001\Eating Architecture.sav'

/BY caseid.

EXECUTE.

SAVE OUTFILE='N:\NDNS pre 2000\NDNS 19-64 yrs 2000-2001\Eating Architecture.sav'

/COMPRESSED.

****Go back to eating occasion file to select snacks and repeat process for deriving the timing and frequency of snack variables.

GET

FILE='N:\NDNS pre 2000\NDNS 19-64 yrs 2000-2001\period0.sav' .

SORT CASES BY caseid dayno .

***Select SNACK eating occasions***.

USE ALL.

SELECT IF (Occasion_Type = 2).

EXECUTE.

***Aggregate timing of eating occasions in the same day***.

AGGREGATE

/OUTFILE='N:\NDNS pre 2000\NDNS 19-64 yrs 2000-2001\aggr.sav'

/PRESORTED

/BREAK=caseid dayno

/TimeMed 'Time of median snack (minutes since midnight)'=MEDIAN(mealtime_minutes)

/TimeFirst 'Time of first snack (minutes since midnight)'=FIRST(mealtime_minutes)

/TimeLast 'Time of last snack (minutes since midnight)'=LAST(mealtime_minutes)

/n_occasions 'Total number of snacks (based on unique times)'=N.

GET

FILE='N:\NDNS pre 2000\NDNS 19-64 yrs 2000-2001\aggr.sav' .

**Transposes the file from one line per day to one line per person with features of days as variables.

CASESTOVARS

/ID=caseid

/GROUPBY=VARIABLE

/COUNT=snack_days "Number of days snacks reported" .

***Replaces any missing values with 0 because these people did report eating on 7 days but simply had 0 snacks on some days, this appears missing in the long file but should be 0.

RECODE n_occasions.1 n_occasions.2 n_occasions.3 n_occasions.4 n_occasions.5 n_occasions.6 n_occasions.7 (SYSMIS=0).

*******************************************************************************************************************************************************************

***Adding in computation of eating period specifically for snacks.

***Manos: Do I also need to compute the mean over 7 days?

******************************************************************************************************************************************************************.

COMPUTE snackEatingPeriod1=TimeLast.1-TimeFirst.1.

VAR LAB snackEatingPeriod1 'Time duration (minutes) over which snack eating occasions occur on day 1)'.

COMPUTE snackEatingPeriod2=TimeLast.2-TimeFirst.2.

VAR LAB snackEatingPeriod2 'Time duration (minutes) over which snack eating occasions occur on day 2)'.

COMPUTE snackEatingPeriod3=TimeLast.3-TimeFirst.3.

VAR LAB snackEatingPeriod3 'Time duration (minutes) over which snack eating occasions occur on day 3)'.

COMPUTE snackEatingPeriod4=TimeLast.4-TimeFirst.4.

VAR LAB snackEatingPeriod4 'Time duration (minutes) over which snack eating occasions occur on day 4)'.

COMPUTE snackEatingPeriod5=TimeLast.5-TimeFirst.5.

VAR LAB snackEatingPeriod5 'Time duration (minutes) over which snack eating occasions occur on day 5)'.

COMPUTE snackEatingPeriod6=TimeLast.6-TimeFirst.6.

VAR LAB snackEatingPeriod6 'Time duration (minutes) over which snack eating occasions occur on day 6)'.

COMPUTE snackEatingPeriod7=TimeLast.7-TimeFirst.7.

VAR LAB snackEatingPeriod7 'Time duration (minutes) over which snack eating occasions occur on day 7)'.

COMPUTE snackEatingPeriod_mean=MEAN(snackEatingPeriod1,snackEatingPeriod2,snackEatingPeriod3,snackEatingPeriod4,snackEatingPeriod5,snackEatingPeriod6,snackEatingPeriod7) .

COMPUTE TimeFirstsnack_mean=MEAN(TimeFirst.1,TimeFirst.2,TimeFirst.3,TimeFirst.4,TimeFirst.5,TimeFirst.6,TimeFirst.7) .

COMPUTE TimeLastsnack_mean=MEAN(TimeLast.1,TimeLast.2,TimeLast.3,TimeLast.4,TimeLast.5,TimeLast.6,TimeLast.7).

VAR LAB snackEatingPeriod_mean 'Mean snack eating period (minutes, last-first time) over 7 days'.

VAR LAB TimeFirstsnack_mean 'Mean first snack time (minutes after midnight) over 7 days'.

VAR LAB TimeLastsnack_mean 'Mean last snack time (minutes after midnight) over 7 days'.

COMPUTE TimeFirstsnack_median=MEDIAN(TimeFirst.1,TimeFirst.2,TimeFirst.3,TimeFirst.4,TimeFirst.5,TimeFirst.6,TimeFirst.7) .

COMPUTE TimeFirstsnack_min=MIN(TimeFirst.1,TimeFirst.2,TimeFirst.3,TimeFirst.4,TimeFirst.5,TimeFirst.6,TimeFirst.7) .

COMPUTE TimeFirstsnack_max=MAX(TimeFirst.1,TimeFirst.2,TimeFirst.3,TimeFirst.4,TimeFirst.5,TimeFirst.6,TimeFirst.7) .

COMPUTE TimeLastsnack_median=MEDIAN(TimeLast.1,TimeLast.2,TimeLast.3,TimeLast.4,TimeLast.5,TimeLast.6,TimeLast.7).

COMPUTE TimeLastsnack_min=MIN(TimeLast.1,TimeLast.2,TimeLast.3,TimeLast.4,TimeLast.5,TimeLast.6,TimeLast.7).

COMPUTE TimeLastsnack_max=MAX(TimeLast.1,TimeLast.2,TimeLast.3,TimeLast.4,TimeLast.5,TimeLast.6,TimeLast.7).

COMPUTE n_snack_mean=MEAN(n_occasions.1,n_occasions.2,n_occasions.3,n_occasions.4,n_occasions.5,n_occasions.6,n_occasions.7).

COMPUTE n_snack_median=MEDIAN(n_occasions.1,n_occasions.2,n_occasions.3,n_occasions.4,n_occasions.5,n_occasions.6,n_occasions.7).

COMPUTE n_snack_SD=SD(n_occasions.1,n_occasions.2,n_occasions.3,n_occasions.4,n_occasions.5,n_occasions.6,n_occasions.7).

COMPUTE n_snack_min=MIN(n_occasions.1,n_occasions.2,n_occasions.3,n_occasions.4,n_occasions.5,n_occasions.6,n_occasions.7).

COMPUTE n_snack_max=MAX(n_occasions.1,n_occasions.2,n_occasions.3,n_occasions.4,n_occasions.5,n_occasions.6,n_occasions.7).

COMPUTE n_snack_range=n_snack_max - n_snack_min.

COMPUTE TimeFirstsnack_range=TimeFirstsnack_max - TimeFirstsnack_min.

COMPUTE TimeLastsnack_range=TimeLastsnack_max - TimeLastsnack_min.

VAR LAB n_snack_range 'Range of number of snacks (times per day) over 7 days'.

VAR LAB TimeFirstsnack_median 'Median first snack time (minutes after midnight) over 7 days'.

VAR LAB TimeFirstsnack_min 'Min first snack time (minutes after midnight) over 7 days'.

VAR LAB TimeFirstsnack_max 'Max first snack time (minutes after midnight) over 7 days'.

VAR LAB TimeFirstsnack_range 'Range first snack time (minutes) over 7 days'.

VAR LAB TimeLastsnack_median 'Median last snack time (minutes after midnight) over 7 days'.

VAR LAB TimeLastsnack_min 'Min last snack time (minutes after midnight) over 7 days'.

VAR LAB TimeLastsnack_max 'Max last snack time (minutes after midnight) over 7 days'.

VAR LAB TimeLastsnack_range 'Range last snack time (minutes) over 7 days'.

VAR LAB n_snack_mean 'Mean snack frequency (times per day) over 7 days'.

VAR LAB n_snack_median 'Median snack frequency (times per day) over 7 days'.

VAR LAB n_snack_SD 'SD snack frequency (times per day) over 7 days'.

VAR LAB n_snack_min 'Min snack frequency (times per day) over 7 days'.

VAR LAB n_snack_max 'Max snack frequency (times per day) over 7 days'.

VAR LAB n_snack_range 'Range snack frequency (times per day) over 7 days'.

EXECUTE.

SORT CASES BY caseid(A).

***Merge new variables into eating architecture variables file.

MATCH FILES /FILE=*

/FILE='N:\NDNS pre 2000\NDNS 19-64 yrs 2000-2001\Eating Architecture.sav'

/BY caseid.

EXECUTE.

SORT CASES BY caseid(A).

SAVE OUTFILE='N:\NDNS pre 2000\NDNS 19-64 yrs 2000-2001\Eating Architecture.sav'

/DROP=dayno.1 dayno.2 dayno.3 dayno.4 dayno.5 dayno.6 dayno.7 TimeMed.1 TimeMed.2 TimeMed.3 TimeMed.4 TimeMed.5 TimeMed.6 TimeMed.7

TimeFirst.1 TimeFirst.2 TimeFirst.3 TimeFirst.4 TimeFirst.5 TimeFirst.6 TimeFirst.7 TimeLast.1 TimeLast.2 TimeLast.3 TimeLast.4 TimeLast.5 TimeLast.6 TimeLast.7

n_occasions.1 n_occasions.2 n_occasions.3 n_occasions.4 n_occasions.5 n_occasions.6 n_occasions.7

/COMPRESSED.

****Go back to eating occasion file to select drinks and repeat process for deriving the timing and frequency of drink variables.

GET

FILE='N:\NDNS pre 2000\NDNS 19-64 yrs 2000-2001\period0.sav' .

SORT CASES BY caseid dayno .

***Counting total number of 60 minute DRINK eating periods per day***.

USE ALL.

SELECT IF (Occasion_Type = 3).

EXECUTE.

***Aggregate timing of eating occasions in the same day***.

AGGREGATE

/OUTFILE='N:\NDNS pre 2000\NDNS 19-64 yrs 2000-2001\aggr.sav'

/PRESORTED

/BREAK=caseid dayno

/TimeMed 'Time of median drink (minutes since midnight)'=MEDIAN(mealtime_minutes)

/TimeFirst 'Time of first drink (minutes since midnight)'=FIRST(mealtime_minutes)

/TimeLast 'Time of last drink (minutes since midnight)'=LAST(mealtime_minutes)

/n_occasions 'Total number of drinks (based on unique times)'=N.

GET

FILE='N:\NDNS pre 2000\NDNS 19-64 yrs 2000-2001\aggr.sav' .

**Transposes the file from one line per day to one line per person with features of days as variables.

CASESTOVARS

/ID=caseid

/GROUPBY=VARIABLE

/COUNT=drink_days "Number of days drinks reported" .

***Replaces any missing values with 0 because these people did report eating on 7 days but simply had 0 drinks on some days, this appears missing in the long file but should be 0.

RECODE n_occasions.1 n_occasions.2 n_occasions.3 n_occasions.4 n_occasions.5 n_occasions.6 n_occasions.7 (SYSMIS=0).

*******************************************************************************************************************************************************************

***Adding in computation of eating period specifically for snacks.

***Manos: Do I also need to compute the mean over 7 days?

******************************************************************************************************************************************************************.

COMPUTE drinkEatingPeriod1=TimeLast.1-TimeFirst.1.

VAR LAB drinkEatingPeriod1 'Time duration (minutes) over which drink eating occasions occur on day 1)'.

COMPUTE drinkEatingPeriod2=TimeLast.2-TimeFirst.2.

VAR LAB drinkEatingPeriod2 'Time duration (minutes) over which drink eating occasions occur on day 2)'.

COMPUTE drinkEatingPeriod3=TimeLast.3-TimeFirst.3.

VAR LAB drinkEatingPeriod3 'Time duration (minutes) over which drink eating occasions occur on day 3)'.

COMPUTE drinkEatingPeriod4=TimeLast.4-TimeFirst.4.

VAR LAB drinkEatingPeriod4 'Time duration (minutes) over which drink eating occasions occur on day 4)'.

COMPUTE drinkEatingPeriod5=TimeLast.5-TimeFirst.5.

VAR LAB drinkEatingPeriod5 'Time duration (minutes) over which drink eating occasions occur on day 5)'.

COMPUTE drinkEatingPeriod6=TimeLast.6-TimeFirst.6.

VAR LAB drinkEatingPeriod6 'Time duration (minutes) over which drink eating occasions occur on day 6)'.

COMPUTE drinkEatingPeriod7=TimeLast.7-TimeFirst.7.

VAR LAB drinkEatingPeriod7 'Time duration (minutes) over which drink eating occasions occur on day 7)'.

COMPUTE drinkEatingPeriod_mean=MEAN(drinkEatingPeriod1,drinkEatingPeriod2,drinkEatingPeriod3,drinkEatingPeriod4,drinkEatingPeriod5,drinkEatingPeriod6,drinkEatingPeriod7) .

COMPUTE TimeFirstdrink_mean=MEAN(TimeFirst.1,TimeFirst.2,TimeFirst.3,TimeFirst.4,TimeFirst.5,TimeFirst.6,TimeFirst.7) .

COMPUTE TimeLastdrink_mean=MEAN(TimeLast.1,TimeLast.2,TimeLast.3,TimeLast.4,TimeLast.5,TimeLast.6,TimeLast.7).

VAR LAB drinkEatingPeriod_mean 'Mean drink eating period (minutes, last-first time) over 7 days'.

VAR LAB TimeFirstdrink_mean 'Mean first drink time (minutes after midnight) over 7 days'.

VAR LAB TimeLastdrink_mean 'Mean last drink time (minutes after midnight) over 7 days'.

COMPUTE TimeFirstdrink_median=MEDIAN(TimeFirst.1,TimeFirst.2,TimeFirst.3,TimeFirst.4,TimeFirst.5,TimeFirst.6,TimeFirst.7) .

COMPUTE TimeFirstdrink_min=MIN(TimeFirst.1,TimeFirst.2,TimeFirst.3,TimeFirst.4,TimeFirst.5,TimeFirst.6,TimeFirst.7) .

COMPUTE TimeFirstdrink_max=MAX(TimeFirst.1,TimeFirst.2,TimeFirst.3,TimeFirst.4,TimeFirst.5,TimeFirst.6,TimeFirst.7) .

COMPUTE TimeLastdrink_median=MEDIAN(TimeLast.1,TimeLast.2,TimeLast.3,TimeLast.4,TimeLast.5,TimeLast.6,TimeLast.7).

COMPUTE TimeLastdrink_min=MIN(TimeLast.1,TimeLast.2,TimeLast.3,TimeLast.4,TimeLast.5,TimeLast.6,TimeLast.7).

COMPUTE TimeLastdrink_max=MAX(TimeLast.1,TimeLast.2,TimeLast.3,TimeLast.4,TimeLast.5,TimeLast.6,TimeLast.7).

COMPUTE n_drink_mean=MEAN(n_occasions.1,n_occasions.2,n_occasions.3,n_occasions.4,n_occasions.5,n_occasions.6,n_occasions.7).

COMPUTE n_drink_median=MEDIAN(n_occasions.1,n_occasions.2,n_occasions.3,n_occasions.4,n_occasions.5,n_occasions.6,n_occasions.7).

COMPUTE n_drink_SD=SD(n_occasions.1,n_occasions.2,n_occasions.3,n_occasions.4,n_occasions.5,n_occasions.6,n_occasions.7).

COMPUTE n_drink_min=MIN(n_occasions.1,n_occasions.2,n_occasions.3,n_occasions.4,n_occasions.5,n_occasions.6,n_occasions.7).

COMPUTE n_drink_max=MAX(n_occasions.1,n_occasions.2,n_occasions.3,n_occasions.4,n_occasions.5,n_occasions.6,n_occasions.7).

COMPUTE n_drink_range=n_drink_max - n_drink_min.

COMPUTE TimeFirstdrink_range=TimeFirstdrink_max - TimeFirstdrink_min.

COMPUTE TimeLastdrink_range=TimeLastdrink_max - TimeLastdrink_min.

VAR LAB n_drink_range 'Range of number of drinks (times per day) over 7 days'.

VAR LAB TimeFirstdrink_median 'Median first drink time (minutes after midnight) over 7 days'.

VAR LAB TimeFirstdrink_min 'Min first drink time (minutes after midnight) over 7 days'.

VAR LAB TimeFirstdrink_max 'Max first drink time (minutes after midnight) over 7 days'.

VAR LAB TimeFirstdrink_range 'Range first drink time (minutes) over 7 days'.

VAR LAB TimeLastdrink_median 'Median last drink time (minutes after midnight) over 7 days'.

VAR LAB TimeLastdrink_min 'Min last drink time (minutes after midnight) over 7 days'.

VAR LAB TimeLastdrink_max 'Max last drink time (minutes after midnight) over 7 days'.

VAR LAB TimeLastdrink_range 'Range last drink time (minutes) over 7 days'.

VAR LAB n_drink_mean 'Mean drink frequency (times per day) over 7 days'.

VAR LAB n_drink_median 'Median drink frequency (times per day) over 7 days'.

VAR LAB n_drink_SD 'SD drink frequency (times per day) over 7 days'.

VAR LAB n_drink_min 'Min drink frequency (times per day) over 7 days'.

VAR LAB n_drink_max 'Max drink frequency (times per day) over 7 days'.

VAR LAB n_drink_range 'Range drink frequency (times per day) over 7 days'.

EXECUTE.

SORT CASES BY caseid(A).

***Merge new variables into eating architecture variables file.

MATCH FILES /FILE=*

/FILE='N:\NDNS pre 2000\NDNS 19-64 yrs 2000-2001\Eating Architecture.sav'

/BY caseid.

EXECUTE.

SORT CASES BY caseid(A).

SAVE OUTFILE='N:\NDNS pre 2000\NDNS 19-64 yrs 2000-2001\Eating Architecture.sav'

/DROP=dayno.1 dayno.2 dayno.3 dayno.4 dayno.5 dayno.6 dayno.7 TimeMed.1 TimeMed.2 TimeMed.3 TimeMed.4 TimeMed.5 TimeMed.6 TimeMed.7

TimeFirst.1 TimeFirst.2 TimeFirst.3 TimeFirst.4 TimeFirst.5 TimeFirst.6 TimeFirst.7 TimeLast.1 TimeLast.2 TimeLast.3 TimeLast.4 TimeLast.5 TimeLast.6 TimeLast.7

n_occasions.1 n_occasions.2 n_occasions.3 n_occasions.4 n_occasions.5 n_occasions.6 n_occasions.7

/COMPRESSED.

***Computing average size of eating occasions per day***.

GET

FILE='N:\NDNS pre 2000\NDNS 19-64 yrs 2000-2001\period0.sav' .

SORT CASES BY caseid .

***Aggregate eating occasion level data to form a day level data file.

AGGREGATE

/OUTFILE='N:\NDNS pre 2000\NDNS 19-64 yrs 2000-2001\aggr.sav'

/PRESORTED

/BREAK=caseid

/AveGrams0P 'Average amount eaten (g) per eating occasion'=MEAN(Grams)

/AveEI_kcal0P 'Energy intake (kcal) per eating occasion'=MEAN(Energy_kcal)

/AveEI_kJ0P 'Energy intake (kJ) per eating occasion'=MEAN(Energy_kJ)

/SDGrams0P 'SD of amount eaten (g) per eating occasion'=SD(Grams)

/SDEI_kcal0P 'SD of eEnergy intake (kcal) per eating occasion'=SD(Energy_kcal)

/MedGrams0P 'Median amount eaten (g) per eating occasion'=MEDIAN(Grams)

/MedEI_kcal0P 'Median Energy intake (kcal) per eating occasion'=MEDIAN(Energy_kcal)

/MedEI_kJ0P 'Median Energy intake (kJ) per eating occasion'=MEDIAN(Energy_kJ)

/MinGrams0P 'Minimum amount eaten (g) per eating occasion'=MIN(Grams)

/MinEI_kcal0P 'Minimum Energy intake (kcal) per eating occasion'=MIN(Energy_kcal)

/MinEI_kJ0P 'Minimum Energy intake (kJ) per eating occasion'=MIN(Energy_kJ)

/MaxGrams0P 'Maximum amount eaten (g) per eating occasion'=MAX(Grams)

/MaxEI_kcal0P 'Maximum Energy intake (kcal) per eating occasion'=MAX(Energy_kcal)

/MaxEI_kJ0P 'Maximum Energy intake (kJ) per eating occasion'=MAX(Energy_kJ).

***Exporting new average eating occasions variable to Eating Architecture file***.

GET

FILE='N:\NDNS pre 2000\NDNS 19-64 yrs 2000-2001\aggr.sav' .

SORT CASES BY caseid(A).

***Merge new variables into eating architecture variables file.

MATCH FILES /FILE=*

/FILE='N:\NDNS pre 2000\NDNS 19-64 yrs 2000-2001\Eating Architecture.sav'

/BY caseid.

EXECUTE.

SORT CASES BY caseid(A).

SAVE OUTFILE='N:\NDNS pre 2000\NDNS 19-64 yrs 2000-2001\Eating Architecture.sav'

/COMPRESSED.

*************************************************************************************************************19/09/2017**************************************************************

***Computing average size of meal eating occasions per day***.

GET

FILE='N:\NDNS pre 2000\NDNS 19-64 yrs 2000-2001\period0.sav' .

***Select only eating occasions classified as meals.

USE ALL.

SELECT IF (Occasion_Type = 1).

EXECUTE.

SORT CASES BY caseid .

***Aggregate eating occasion level data to form a day level data file.

AGGREGATE

/OUTFILE='N:\NDNS pre 2000\NDNS 19-64 yrs 2000-2001\aggr.sav'

/PRESORTED

/BREAK=caseid

/mealAveGrams0P 'Method 1: Average amount eaten (g) per meal eating occasion'=MEAN(Grams)

/mealAveEI_kcal0P 'Method 1: Energy intake (kcal) per meal eating occasion'=MEAN(Energy_kcal)

/mealAveEI_kJ0P 'Method 1: Energy intake (kJ) per meal eating occasion'=MEAN(Energy_kJ)

/mealSDGrams0P 'Method 1: SD of amount eaten (g) per meal eating occasion'=SD(Grams)

/mealSDEI_kcal0P 'Method 1: SD of energy intake (kcal) per meal eating occasion'=SD(Energy_kcal)

/mealSDEI_kJ0P 'Method 1: SD of energy intake (kJ) per meal eating occasion'=SD(Energy_kJ)

/mealMedGrams0P 'Method 1: Median amount eaten (g) per Meal eating occasion'=MEDIAN(Grams)

/mealMedEI_kcal0P 'Method 1: Median Energy intake (kcal) per Meal eating occasion'=MEDIAN(Energy_kcal)

/mealMedEI_kJ0P 'Method 1: Median Energy intake (kJ) per Meal eating occasion'=MEDIAN(Energy_kJ)

/mealMinGrams0P 'Method 1: Minimum amount eaten (g) per Meal eating occasion'=MIN(Grams)

/mealMinEI_kcal0P 'Method 1: Minimum Energy intake (kcal) per Meal eating occasion'=MIN(Energy_kcal)

/mealMinEI_kJ0P 'Method 1: Minimum Energy intake (kJ) per Meal eating occasion'=MIN(Energy_kJ)

/mealMaxGrams0P 'Method 1: Maximum amount eaten (g) per Meal eating occasion'=MAX(Grams)

/mealMaxEI_kcal0P 'Method 1: Maximum Energy intake (kcal) per Meal eating occasion'=MAX(Energy_kcal)

/mealMaxEI_kJ0P 'Method 1: Maximum Energy intake (kJ) per Meal eating occasion'=MAX(Energy_kJ).

***Exporting new average eating occasions variable to Eating Architecture file***.

GET

FILE='N:\NDNS pre 2000\NDNS 19-64 yrs 2000-2001\aggr.sav' .

SORT CASES BY caseid(A).

***Merge new variables into eating architecture variables file.

MATCH FILES /FILE=*

/FILE='N:\NDNS pre 2000\NDNS 19-64 yrs 2000-2001\Eating Architecture.sav'

/BY caseid.

EXECUTE.

SORT CASES BY caseid(A).

SAVE OUTFILE='N:\NDNS pre 2000\NDNS 19-64 yrs 2000-2001\Eating Architecture.sav'

/COMPRESSED.

****Need to compute size variables but to account for days when people eat no meals, snacks or drink we need to first collapse to day level then transpose to person level then recode missing and then compute mean, median, etc.

***Computing average size of MEAL eating occasions per day***.

GET

FILE='N:\NDNS pre 2000\NDNS 19-64 yrs 2000-2001\period0.sav' .

***Select only eating occasions classified as meals.

USE ALL.

SELECT IF (Occasion_Type = 1).

EXECUTE.

SORT CASES BY caseid .

***Aggregate size of meal eating occasions in the same day: EO level to day level***.

AGGREGATE

/OUTFILE='N:\NDNS pre 2000\NDNS 19-64 yrs 2000-2001\aggr.sav'

/PRESORTED

/BREAK=caseid dayno

/AveGrams0P 'Average amount eaten (g) per eating occasion'=MEAN(Grams)

/AveEI_kcal0P 'Energy intake (kcal) per eating occasion'=MEAN(Energy_kcal)

/AveEI_kJ0P 'Energy intake (kJ) per eating occasion'=MEAN(Energy_kJ)

/SDGrams0P 'SD of amount eaten (g) per eating occasion'=SD(Grams)

/SDEI_kcal0P 'SD of eEnergy intake (kcal) per eating occasion'=SD(Energy_kcal)

/SDEI_kJ0P 'SD of energy intake (kJ) per meal eating occasion'=SD(Energy_kJ)

/MedGrams0P 'Median amount eaten (g) per eating occasion'=MEDIAN(Grams)

/MedEI_kcal0P 'Median Energy intake (kcal) per eating occasion'=MEDIAN(Energy_kcal)

/MedEI_kJ0P 'Median Energy intake (kJ) per eating occasion'=MEDIAN(Energy_kJ)

/MinGrams0P 'Minimum amount eaten (g) per eating occasion'=MIN(Grams)

/MinEI_kcal0P 'Minimum Energy intake (kcal) per eating occasion'=MIN(Energy_kcal)

/MinEI_kJ0P 'Minimum Energy intake (kJ) per eating occasion'=MIN(Energy_kJ)

/MaxGrams0P 'Maximum amount eaten (g) per eating occasion'=MAX(Grams)

/MaxEI_kcal0P 'Maximum Energy intake (kcal) per eating occasion'=MAX(Energy_kcal)

/MaxEI_kJ0P 'Maximum Energy intake (kJ) per eating occasion'=MAX(Energy_kJ).

GET

FILE='N:\NDNS pre 2000\NDNS 19-64 yrs 2000-2001\aggr.sav' .

COMPUTE range_Grams0P = MaxGrams0P - MinGrams0P .

COMPUTE range_EI_kcal0P = MaxEI_kcal0P - MinEI_kcal0P .

COMPUTE range_EI_kJ0P = MaxEI_kJ0P - MinEI_kJ0P .

VAR LAB range_Grams0P 'Range of grams per meal over 1 day'.

VAR LAB range_EI_kcal0P 'Range of kcal per meal over 1 day'.

VAR LAB range_EI_kJ0P 'Range of kJ per meal over 1 day'.

**Transposes the file from one line per day to one line per person with features of days as variables.

CASESTOVARS

/ID=caseid

/GROUPBY=VARIABLE

/COUNT=days "Number of days" .

***Replaces any missing values with 0 because these people did report eating on 7 days but simply had 0 meals/snacks/drinks on some days, this appears missing in the long file but should be 0.

RECODE AveGrams0P.1 AveGrams0P.2 AveGrams0P.3 AveGrams0P.4 AveGrams0P.5 AveGrams0P.6 AveGrams0P.7

AveEI_kcal0P.1 AveEI_kcal0P.2 AveEI_kcal0P.3 AveEI_kcal0P.4 AveEI_kcal0P.5 AveEI_kcal0P.6 AveEI_kcal0P.7

AveEI_kJ0P.1 AveEI_kJ0P.2 AveEI_kJ0P.3 AveEI_kJ0P.4 AveEI_kJ0P.5 AveEI_kJ0P.6 AveEI_kJ0P.7

SDGrams0P.1 SDGrams0P.2 SDGrams0P.3 SDGrams0P.4 SDGrams0P.5 SDGrams0P.6 SDGrams0P.7

SDEI_kcal0P.1 SDEI_kcal0P.2 SDEI_kcal0P.3 SDEI_kcal0P.4 SDEI_kcal0P.5 SDEI_kcal0P.6 SDEI_kcal0P.7

SDEI_kJ0P.1 SDEI_kJ0P.2 SDEI_kJ0P.3 SDEI_kJ0P.4 SDEI_kJ0P.5 SDEI_kJ0P.6 SDEI_kJ0P.7

MedGrams0P.1 MedGrams0P.2 MedGrams0P.3 MedGrams0P.4 MedGrams0P.5 MedGrams0P.6 MedGrams0P.7

MedEI_kcal0P.1 MedEI_kcal0P.2 MedEI_kcal0P.3 MedEI_kcal0P.4 MedEI_kcal0P.5 MedEI_kcal0P.6 MedEI_kcal0P.7

MedEI_kJ0P.1 MedEI_kJ0P.2 MedEI_kJ0P.3 MedEI_kJ0P.4 MedEI_kJ0P.5 MedEI_kJ0P.6 MedEI_kJ0P.7

MinGrams0P.1 MinGrams0P.2 MinGrams0P.3 MinGrams0P.4 MinGrams0P.5 MinGrams0P.6 MinGrams0P.7

MinEI_kcal0P.1 MinEI_kcal0P.2 MinEI_kcal0P.3 MinEI_kcal0P.4 MinEI_kcal0P.5 MinEI_kcal0P.6 MinEI_kcal0P.7

MinEI_kJ0P.1 MinEI_kJ0P.2 MinEI_kJ0P.3 MinEI_kJ0P.4 MinEI_kJ0P.5 MinEI_kJ0P.6 MinEI_kJ0P.7

MaxGrams0P.1 MaxGrams0P.2 MaxGrams0P.3 MaxGrams0P.4 MaxGrams0P.5 MaxGrams0P.6 MaxGrams0P.7

MaxEI_kcal0P.1 MaxEI_kcal0P.2 MaxEI_kcal0P.3 MaxEI_kcal0P.4 MaxEI_kcal0P.5 MaxEI_kcal0P.6 MaxEI_kcal0P.7

MaxEI_kJ0P.1 MaxEI_kJ0P.2 MaxEI_kJ0P.3 MaxEI_kJ0P.4 MaxEI_kJ0P.5 MaxEI_kJ0P.6 MaxEI_kJ0P.7

range_Grams0P.1 range_Grams0P.2 range_Grams0P.3 range_Grams0P.4 range_Grams0P.5 range_Grams0P.6 range_Grams0P.7

range_EI_kcal0P.1 range_EI_kcal0P.2 range_EI_kcal0P.3 range_EI_kcal0P.4 range_EI_kcal0P.5 range_EI_kcal0P.6 range_EI_kcal0P.7

range_EI_kJ0P.1 range_EI_kJ0P.2 range_EI_kJ0P.3 range_EI_kJ0P.4 range_EI_kJ0P.5 range_EI_kJ0P.6 range_EI_kJ0P.7

(SYSMIS=0).

COMPUTE MeanGrams0Pmeal=Mean(AveGrams0P.1, AveGrams0P.2, AveGrams0P.3, AveGrams0P.4, AveGrams0P.5, AveGrams0P.6, AveGrams0P.7) .

VAR LAB MeanGrams0Pmeal 'Mean mean amount eaten (g) per meal eating occasion in a day over 7 days'.

COMPUTE MeanEI_kcal0Pmeal=Mean(AveEI_kcal0P.1, AveEI_kcal0P.2, AveEI_kcal0P.3, AveEI_kcal0P.4, AveEI_kcal0P.5, AveEI_kcal0P.6, AveEI_kcal0P.7) .

VAR LAB MeanEI_kcal0Pmeal 'Mean mean amount eaten (kcal) per meal eating occasion in a day over 7 days'.

COMPUTE MeanEI_kJ0Pmeal=Mean(AveEI_kJ0P.1, AveEI_kJ0P.2, AveEI_kJ0P.3, AveEI_kJ0P.4, AveEI_kJ0P.5, AveEI_kJ0P.6, AveEI_kJ0P.7) .

VAR LAB MeanEI_kJ0Pmeal 'Mean mean amount eaten (kJ) per meal eating occasion in a day over 7 days'.

COMPUTE SDGrams0Pmeal=SD(AveGrams0P.1, AveGrams0P.2, AveGrams0P.3, AveGrams0P.4, AveGrams0P.5, AveGrams0P.6, AveGrams0P.7) .

VAR LAB SDGrams0Pmeal 'SD mean amount eaten (g) per meal eating occasion in a day over 7 days'.

COMPUTE SDEI_kcal0Pmeal=SD(AveEI_kcal0P.1, AveEI_kcal0P.2, AveEI_kcal0P.3, AveEI_kcal0P.4, AveEI_kcal0P.5, AveEI_kcal0P.6, AveEI_kcal0P.7) .

VAR LAB SDEI_kcal0Pmeal 'SD mean amount eaten (kcal) per meal eating occasion in a day over 7 days'.

COMPUTE SDEI_kJ0Pmeal=SD(AveEI_kJ0P.1, AveEI_kJ0P.2, AveEI_kJ0P.3, AveEI_kJ0P.4, AveEI_kJ0P.5, AveEI_kJ0P.6, AveEI_kJ0P.7) .

VAR LAB SDEI_kJ0Pmeal 'SD mean amount eaten (kJ) per meal eating occasion in a day over 7 days'.

COMPUTE medianGrams0Pmeal=median(AveGrams0P.1, AveGrams0P.2, AveGrams0P.3, AveGrams0P.4, AveGrams0P.5, AveGrams0P.6, AveGrams0P.7) .

VAR LAB medianGrams0Pmeal 'median mean amount eaten (g) per meal eating occasion in a day over 7 days'.

COMPUTE medianEI_kcal0Pmeal=median(AveEI_kcal0P.1, AveEI_kcal0P.2, AveEI_kcal0P.3, AveEI_kcal0P.4, AveEI_kcal0P.5, AveEI_kcal0P.6, AveEI_kcal0P.7) .

VAR LAB medianEI_kcal0Pmeal 'median mean amount eaten (kcal) per meal eating occasion in a day over 7 days'.

COMPUTE medianEI_kJ0Pmeal=median(AveEI_kJ0P.1, AveEI_kJ0P.2, AveEI_kJ0P.3, AveEI_kJ0P.4, AveEI_kJ0P.5, AveEI_kJ0P.6, AveEI_kJ0P.7) .

VAR LAB medianEI_kJ0Pmeal 'median mean amount eaten (kJ) per meal eating occasion in a day over 7 days'.

COMPUTE minGrams0Pmeal=min(AveGrams0P.1, AveGrams0P.2, AveGrams0P.3, AveGrams0P.4, AveGrams0P.5, AveGrams0P.6, AveGrams0P.7) .

VAR LAB minGrams0Pmeal 'min mean amount eaten (g) per meal eating occasion in a day over 7 days'.

COMPUTE minEI_kcal0Pmeal=min(AveEI_kcal0P.1, AveEI_kcal0P.2, AveEI_kcal0P.3, AveEI_kcal0P.4, AveEI_kcal0P.5, AveEI_kcal0P.6, AveEI_kcal0P.7) .

VAR LAB minEI_kcal0Pmeal 'min mean amount eaten (kcal) per meal eating occasion in a day over 7 days'.

COMPUTE minEI_kJ0Pmeal=min(AveEI_kJ0P.1, AveEI_kJ0P.2, AveEI_kJ0P.3, AveEI_kJ0P.4, AveEI_kJ0P.5, AveEI_kJ0P.6, AveEI_kJ0P.7) .

VAR LAB minEI_kJ0Pmeal 'min mean amount eaten (kJ) per meal eating occasion in a day over 7 days'.

COMPUTE maxGrams0Pmeal=max(AveGrams0P.1, AveGrams0P.2, AveGrams0P.3, AveGrams0P.4, AveGrams0P.5, AveGrams0P.6, AveGrams0P.7) .

VAR LAB maxGrams0Pmeal 'max mean amount eaten (g) per meal eating occasion in a day over 7 days'.

COMPUTE maxEI_kcal0Pmeal=max(AveEI_kcal0P.1, AveEI_kcal0P.2, AveEI_kcal0P.3, AveEI_kcal0P.4, AveEI_kcal0P.5, AveEI_kcal0P.6, AveEI_kcal0P.7) .

VAR LAB maxEI_kcal0Pmeal 'max mean amount eaten (kcal) per meal eating occasion in a day over 7 days'.

COMPUTE maxEI_kJ0Pmeal=max(AveEI_kJ0P.1, AveEI_kJ0P.2, AveEI_kJ0P.3, AveEI_kJ0P.4, AveEI_kJ0P.5, AveEI_kJ0P.6, AveEI_kJ0P.7) .

VAR LAB maxEI_kJ0Pmeal 'max mean amount eaten (kJ) per meal eating occasion in a day over 7 days'.

COMPUTE meanSDGrams0Pmeal=Mean(SDGrams0P.1, SDGrams0P.2, SDGrams0P.3, SDGrams0P.4, SDGrams0P.5, SDGrams0P.6, SDGrams0P.7) .

VAR LAB meanSDGrams0Pmeal 'Mean variation in amount eaten (g) per meal eating occasion in a day over 7 days'.

COMPUTE meanSDEI_kcal0Pmeal=Mean(SDEI_kcal0P.1, SDEI_kcal0P.2, SDEI_kcal0P.3, SDEI_kcal0P.4, SDEI_kcal0P.5, SDEI_kcal0P.6, SDEI_kcal0P.7) .

VAR LAB meanSDEI_kcal0Pmeal 'Mean variation in amount eaten (kcal) per meal eating occasion in a day over 7 days'.

COMPUTE meanSDEI_kJ0Pmeal=Mean(SDEI_kJ0P.1, SDEI_kJ0P.2, SDEI_kJ0P.3, SDEI_kJ0P.4, SDEI_kJ0P.5, SDEI_kJ0P.6, SDEI_kJ0P.7) .

VAR LAB meanSDEI_kJ0Pmeal 'Mean variation in amount eaten (kJ) per meal eating occasion in a day over 7 days'.

COMPUTE MedGrams0Pmeal=MEDIAN(MedGrams0P.1, MedGrams0P.2, MedGrams0P.3, MedGrams0P.4, MedGrams0P.5, MedGrams0P.6, MedGrams0P.7) .

VAR LAB MedGrams0Pmeal 'Median amount eaten (g) per meal eating occasion in a day over 7 days'.

COMPUTE MedEI_kcal0Pmeal=MEDIAN(MedEI_kcal0P.1, MedEI_kcal0P.2, MedEI_kcal0P.3, MedEI_kcal0P.4, MedEI_kcal0P.5, MedEI_kcal0P.6, MedEI_kcal0P.7) .

VAR LAB MedEI_kcal0Pmeal 'Median amount eaten (kcal) per meal eating occasion in a day over 7 days'.

COMPUTE MedEI_kJ0Pmeal=MEDIAN(MedEI_kJ0P.1, MedEI_kJ0P.2, MedEI_kJ0P.3, MedEI_kJ0P.4, MedEI_kJ0P.5, MedEI_kJ0P.6, MedEI_kJ0P.7) .

VAR LAB MedEI_kJ0Pmeal 'Median amount eaten (kJ) per meal eating occasion in a day over 7 days'.

COMPUTE MinGrams0Pmeal=MEDIAN(MinGrams0P.1, MinGrams0P.2, MinGrams0P.3, MinGrams0P.4, MinGrams0P.5, MinGrams0P.6, MinGrams0P.7) .

VAR LAB MinGrams0Pmeal 'Median minimum amount eaten (g) per meal eating occasion in a day over 7 days'.

COMPUTE MinEI_kcal0Pmeal=MEDIAN(MinEI_kcal0P.1, MinEI_kcal0P.2, MinEI_kcal0P.3, MinEI_kcal0P.4, MinEI_kcal0P.5, MinEI_kcal0P.6, MinEI_kcal0P.7) .

VAR LAB MinEI_kcal0Pmeal 'Median minimum amount eaten (kcal) per meal eating occasion in a day over 7 days'.

COMPUTE MinEI_kJ0Pmeal=MEDIAN(MinEI_kJ0P.1, MinEI_kJ0P.2, MinEI_kJ0P.3, MinEI_kJ0P.4, MinEI_kJ0P.5, MinEI_kJ0P.6, MinEI_kJ0P.7) .

VAR LAB MinEI_kJ0Pmeal 'Median minimum amount eaten (kJ) per meal eating occasion in a day over 7 days'.

COMPUTE MaxGrams0Pmeal=MEDIAN(MaxGrams0P.1, MaxGrams0P.2, MaxGrams0P.3, MaxGrams0P.4, MaxGrams0P.5, MaxGrams0P.6, MaxGrams0P.7) .

VAR LAB MaxGrams0Pmeal 'Median maximum amount eaten (g) per meal eating occasion in a day over 7 days'.

COMPUTE MaxEI_kcal0Pmeal=MEDIAN(MaxEI_kcal0P.1, MaxEI_kcal0P.2, MaxEI_kcal0P.3, MaxEI_kcal0P.4, MaxEI_kcal0P.5, MaxEI_kcal0P.6, MaxEI_kcal0P.7) .

VAR LAB MaxEI_kcal0Pmeal 'Median maximum amount eaten (kcal) per meal eating occasion in a day over 7 days'.

COMPUTE MaxEI_kJ0Pmeal=MEDIAN(MaxEI_kJ0P.1, MaxEI_kJ0P.2, MaxEI_kJ0P.3, MaxEI_kJ0P.4, MaxEI_kJ0P.5, MaxEI_kJ0P.6, MaxEI_kJ0P.7) .

VAR LAB MaxEI_kJ0Pmeal 'Median maximum amount eaten (kJ) per meal eating occasion in a day over 7 days'.

COMPUTE range_Grams0Pmeal=MEDIAN(range_Grams0P.1, range_Grams0P.2, range_Grams0P.3, range_Grams0P.4, range_Grams0P.5, range_Grams0P.6, range_Grams0P.7) .

VAR LAB range_Grams0Pmeal 'Median range amount eaten (g) per meal eating occasion in a day over 7 days'.

COMPUTE range_EI_kcal0Pmeal=MEDIAN(range_EI_kcal0P.1, range_EI_kcal0P.2, range_EI_kcal0P.3, range_EI_kcal0P.4, range_EI_kcal0P.5, range_EI_kcal0P.6, range_EI_kcal0P.7) .

VAR LAB range_EI_kcal0Pmeal 'Median range amount eaten (kcal) per meal eating occasion in a day over 7 days'.

COMPUTE range_EI_kJ0Pmeal=MEDIAN(range_EI_kJ0P.1, range_EI_kJ0P.2, range_EI_kJ0P.3, range_EI_kJ0P.4, range_EI_kJ0P.5, range_EI_kJ0P.6, range_EI_kJ0P.7) .

VAR LAB range_EI_kJ0Pmeal 'Median range amount eaten (kJ) per meal eating occasion in a day over 7 days'.

EXECUTE.

SORT CASES BY caseid(A).

***Merge new variables into eating architecture variables file.

MATCH FILES /FILE=*

/FILE='N:\NDNS pre 2000\NDNS 19-64 yrs 2000-2001\Eating Architecture.sav'

/BY caseid.

EXECUTE.

SORT CASES BY caseid(A).

SAVE OUTFILE='N:\NDNS pre 2000\NDNS 19-64 yrs 2000-2001\Eating Architecture.sav'

/DROP=dayno.1 dayno.2 dayno.3 dayno.4 dayno.5 dayno.6 dayno.7 AveGrams0P.1 AveGrams0P.2 AveGrams0P.3 AveGrams0P.4 AveGrams0P.5 AveGrams0P.6 AveGrams0P.7

AveEI_kcal0P.1 AveEI_kcal0P.2 AveEI_kcal0P.3 AveEI_kcal0P.4 AveEI_kcal0P.5 AveEI_kcal0P.6 AveEI_kcal0P.7 AveEI_kJ0P.1 AveEI_kJ0P.2 AveEI_kJ0P.3 AveEI_kJ0P.4 AveEI_kJ0P.5 AveEI_kJ0P.6 AveEI_kJ0P.7

SDGrams0P.1 SDGrams0P.2 SDGrams0P.3 SDGrams0P.4 SDGrams0P.5 SDGrams0P.6 SDGrams0P.7 SDEI_kcal0P.1 SDEI_kcal0P.2 SDEI_kcal0P.3 SDEI_kcal0P.4 SDEI_kcal0P.5 SDEI_kcal0P.6 SDEI_kcal0P.7

SDEI_kJ0P.1 SDEI_kJ0P.2 SDEI_kJ0P.3 SDEI_kJ0P.4 SDEI_kJ0P.5 SDEI_kJ0P.6 SDEI_kJ0P.7 MedGrams0P.1 MedGrams0P.2 MedGrams0P.3 MedGrams0P.4 MedGrams0P.5 MedGrams0P.6 MedGrams0P.7

MedEI_kcal0P.1 MedEI_kcal0P.2 MedEI_kcal0P.3 MedEI_kcal0P.4 MedEI_kcal0P.5 MedEI_kcal0P.6 MedEI_kcal0P.7 MedEI_kJ0P.1 MedEI_kJ0P.2 MedEI_kJ0P.3 MedEI_kJ0P.4 MedEI_kJ0P.5 MedEI_kJ0P.6 MedEI_kJ0P.7

MinGrams0P.1 MinGrams0P.2 MinGrams0P.3 MinGrams0P.4 MinGrams0P.5 MinGrams0P.6 MinGrams0P.7 MinEI_kcal0P.1 MinEI_kcal0P.2 MinEI_kcal0P.3 MinEI_kcal0P.4 MinEI_kcal0P.5 MinEI_kcal0P.6 MinEI_kcal0P.7

MinEI_kJ0P.1 MinEI_kJ0P.2 MinEI_kJ0P.3 MinEI_kJ0P.4 MinEI_kJ0P.5 MinEI_kJ0P.6 MinEI_kJ0P.7 MaxGrams0P.1 MaxGrams0P.2 MaxGrams0P.3 MaxGrams0P.4 MaxGrams0P.5 MaxGrams0P.6 MaxGrams0P.7

MaxEI_kcal0P.1 MaxEI_kcal0P.2 MaxEI_kcal0P.3 MaxEI_kcal0P.4 MaxEI_kcal0P.5 MaxEI_kcal0P.6 MaxEI_kcal0P.7 MaxEI_kJ0P.1 MaxEI_kJ0P.2 MaxEI_kJ0P.3 MaxEI_kJ0P.4 MaxEI_kJ0P.5 MaxEI_kJ0P.6 MaxEI_kJ0P.7

range_Grams0P.1 range_Grams0P.2 range_Grams0P.3 range_Grams0P.4 range_Grams0P.5 range_Grams0P.6 range_Grams0P.7 range_EI_kcal0P.1 range_EI_kcal0P.2 range_EI_kcal0P.3 range_EI_kcal0P.4 range_EI_kcal0P.5

range_EI_kcal0P.6 range_EI_kcal0P.7 range_EI_kJ0P.1 range_EI_kJ0P.2 range_EI_kJ0P.3 range_EI_kJ0P.4 range_EI_kJ0P.5 range_EI_kJ0P.6 range_EI_kJ0P.7

/COMPRESSED.

***Checks to understand the difference in variables generated by aggregating all EOs over 7 days, vs. aggregating over a day, then transposing, recoding missing to 0 and then computing mean, SD, etc.

GET

FILE='N:\NDNS pre 2000\NDNS 19-64 yrs 2000-2001\Eating Architecture.sav'.

COMPUTE diffAveGrams=MeanGrams0Pmeal - mealAveGrams0P.

COMPUTE diffAveKcal=MeanEI_kcal0Pmeal - mealAveEI_kcal0P .

COMPUTE diffAveKJ=MeanEI_kJ0Pmeal - mealAveEI_kJ0P .

COMPUTE diffSDgrams=meanSDGrams0Pmeal - mealSDGrams0P .

COMPUTE diffSDEI_kcal =meanSDEI_kcal0Pmeal - mealSDEI_kcal0P .

COMPUTE diffSDEI_kJ =meanSDEI_kJ0Pmeal - mealSDEI_kJ0P .

COMPUTE diffMedGrams =MedGrams0Pmeal - mealMedGrams0P .

COMPUTE diffMedEI_kcal =MedEI_kcal0Pmeal - mealMedEI_kcal0P .

COMPUTE diffMedEI_kJ =MedEI_kJ0Pmeal - mealMedEI_kJ0P .

COMPUTE diffMinGrams =MinGrams0Pmeal - mealMinGrams0P .

COMPUTE diffMinEI_kcal =MinEI_kcal0Pmeal - mealMinEI_kcal0P .

COMPUTE diffMinEI_kJ =MinEI_kJ0Pmeal - mealMinEI_kJ0P .

COMPUTE diffMaxGrams =MaxGrams0Pmeal - mealMaxGrams0P .

COMPUTE diffMaxEI_kcal =MaxEI_kcal0Pmeal - mealMaxEI_kcal0P .

COMPUTE diffMaxEI_kJ =MaxEI_kJ0Pmeal - mealMaxEI_kJ0P .

FREQUENCIES VARIABLES=diffAveGrams diffAveKcal diffAveKJ diffSDgrams diffSDEI_kcal diffSDEI_kJ diffMedGrams diffMedEI_kcal diffMedEI_kJ diffMinGrams diffMinEI_kcal

diffMinEI_kJ diffMaxGrams diffMaxEI_kcal diffMaxEI_kJ

/FORMAT=NOTABLE

/NTILES=4

/STATISTICS=STDDEV MINIMUM MAXIMUM MEAN

/ORDER=ANALYSIS.

EXECUTE.

*****Need to compute size variables but to account for days when people eat no meals, snacks or drink we need to first collapse to day level then transpose to person level then recode missing and then compute mean, median, etc.

***Computing average size of snack eating occasions per day***.

GET

FILE='N:\NDNS pre 2000\NDNS 19-64 yrs 2000-2001\period0.sav' .

***Select only eating occasions classified as snacks.

USE ALL.

SELECT IF (Occasion_Type = 2).

EXECUTE.

SORT CASES BY caseid .

***Aggregate size of snack eating occasions in the same day: EO level to day level***.

AGGREGATE

/OUTFILE='N:\NDNS pre 2000\NDNS 19-64 yrs 2000-2001\aggr.sav'

/PRESORTED

/BREAK=caseid dayno

/AveGrams0P 'Average amount eaten (g) per snack eating occasion'=MEAN(Grams)

/AveEI_kcal0P 'Energy intake (kcal) per snack eating occasion'=MEAN(Energy_kcal)

/AveEI_kJ0P 'Energy intake (kJ) per snack eating occasion'=MEAN(Energy_kJ)

/SDGrams0P 'SD of amount eaten (g) per snack eating occasion'=SD(Grams)

/SDEI_kcal0P 'SD of energy intake (kcal) per snack eating occasion'=SD(Energy_kcal)

/SDEI_kJ0P 'SD of energy intake (kJ) per snack eating occasion'=SD(Energy_kJ)

/MedGrams0P 'Median amount eaten (g) per snack eating occasion'=MEDIAN(Grams)

/MedEI_kcal0P 'Median Energy intake (kcal) per snack eating occasion'=MEDIAN(Energy_kcal)

/MedEI_kJ0P 'Median Energy intake (kJ) per snack eating occasion'=MEDIAN(Energy_kJ)

/MinGrams0P 'Minimum amount eaten (g) per snack eating occasion'=MIN(Grams)

/MinEI_kcal0P 'Minimum Energy intake (kcal) per snack eating occasion'=MIN(Energy_kcal)

/MinEI_kJ0P 'Minimum Energy intake (kJ) per snack eating occasion'=MIN(Energy_kJ)

/MaxGrams0P 'Maximum amount eaten (g) per snack eating occasion'=MAX(Grams)

/MaxEI_kcal0P 'Maximum Energy intake (kcal) per snack eating occasion'=MAX(Energy_kcal)

/MaxEI_kJ0P 'Maximum Energy intake (kJ) per snack eating occasion'=MAX(Energy_kJ).

GET

FILE='N:\NDNS pre 2000\NDNS 19-64 yrs 2000-2001\aggr.sav' .

COMPUTE range_Grams0P = MaxGrams0P - MinGrams0P .

COMPUTE range_EI_kcal0P = MaxEI_kcal0P - MinEI_kcal0P .

COMPUTE range_EI_kJ0P = MaxEI_kJ0P - MinEI_kJ0P .

VAR LAB range_Grams0P 'Range of grams per snack over 1 day'.

VAR LAB range_EI_kcal0P 'Range of kcal per snack over 1 day'.

VAR LAB range_EI_kJ0P 'Range of kJ per snack over 1 day'.

**Transposes the file from one line per day to one line per person with features of days as variables.

CASESTOVARS

/ID=caseid

/GROUPBY=VARIABLE

/COUNT=days "Number of days" .

***Replaces any missing values with 0 because these people did report eating on 7 days but simply had 0 meals/snacks/drinks on some days, this appears missing in the long file but should be 0.

RECODE AveGrams0P.1 AveGrams0P.2 AveGrams0P.3 AveGrams0P.4 AveGrams0P.5 AveGrams0P.6 AveGrams0P.7

AveEI_kcal0P.1 AveEI_kcal0P.2 AveEI_kcal0P.3 AveEI_kcal0P.4 AveEI_kcal0P.5 AveEI_kcal0P.6 AveEI_kcal0P.7

AveEI_kJ0P.1 AveEI_kJ0P.2 AveEI_kJ0P.3 AveEI_kJ0P.4 AveEI_kJ0P.5 AveEI_kJ0P.6 AveEI_kJ0P.7

SDGrams0P.1 SDGrams0P.2 SDGrams0P.3 SDGrams0P.4 SDGrams0P.5 SDGrams0P.6 SDGrams0P.7

SDEI_kcal0P.1 SDEI_kcal0P.2 SDEI_kcal0P.3 SDEI_kcal0P.4 SDEI_kcal0P.5 SDEI_kcal0P.6 SDEI_kcal0P.7

SDEI_kJ0P.1 SDEI_kJ0P.2 SDEI_kJ0P.3 SDEI_kJ0P.4 SDEI_kJ0P.5 SDEI_kJ0P.6 SDEI_kJ0P.7

MedGrams0P.1 MedGrams0P.2 MedGrams0P.3 MedGrams0P.4 MedGrams0P.5 MedGrams0P.6 MedGrams0P.7

MedEI_kcal0P.1 MedEI_kcal0P.2 MedEI_kcal0P.3 MedEI_kcal0P.4 MedEI_kcal0P.5 MedEI_kcal0P.6 MedEI_kcal0P.7

MedEI_kJ0P.1 MedEI_kJ0P.2 MedEI_kJ0P.3 MedEI_kJ0P.4 MedEI_kJ0P.5 MedEI_kJ0P.6 MedEI_kJ0P.7

MinGrams0P.1 MinGrams0P.2 MinGrams0P.3 MinGrams0P.4 MinGrams0P.5 MinGrams0P.6 MinGrams0P.7

MinEI_kcal0P.1 MinEI_kcal0P.2 MinEI_kcal0P.3 MinEI_kcal0P.4 MinEI_kcal0P.5 MinEI_kcal0P.6 MinEI_kcal0P.7

MinEI_kJ0P.1 MinEI_kJ0P.2 MinEI_kJ0P.3 MinEI_kJ0P.4 MinEI_kJ0P.5 MinEI_kJ0P.6 MinEI_kJ0P.7

MaxGrams0P.1 MaxGrams0P.2 MaxGrams0P.3 MaxGrams0P.4 MaxGrams0P.5 MaxGrams0P.6 MaxGrams0P.7

MaxEI_kcal0P.1 MaxEI_kcal0P.2 MaxEI_kcal0P.3 MaxEI_kcal0P.4 MaxEI_kcal0P.5 MaxEI_kcal0P.6 MaxEI_kcal0P.7

MaxEI_kJ0P.1 MaxEI_kJ0P.2 MaxEI_kJ0P.3 MaxEI_kJ0P.4 MaxEI_kJ0P.5 MaxEI_kJ0P.6 MaxEI_kJ0P.7

range_Grams0P.1 range_Grams0P.2 range_Grams0P.3 range_Grams0P.4 range_Grams0P.5 range_Grams0P.6 range_Grams0P.7

range_EI_kcal0P.1 range_EI_kcal0P.2 range_EI_kcal0P.3 range_EI_kcal0P.4 range_EI_kcal0P.5 range_EI_kcal0P.6 range_EI_kcal0P.7

range_EI_kJ0P.1 range_EI_kJ0P.2 range_EI_kJ0P.3 range_EI_kJ0P.4 range_EI_kJ0P.5 range_EI_kJ0P.6 range_EI_kJ0P.7

(SYSMIS=0).

COMPUTE MeanGrams0Psnack=Mean(AveGrams0P.1, AveGrams0P.2, AveGrams0P.3, AveGrams0P.4, AveGrams0P.5, AveGrams0P.6, AveGrams0P.7) .

VAR LAB MeanGrams0Psnack 'Mean mean amount eaten (g) per snack eating occasion in a day over 7 days'.

COMPUTE MeanEI_kcal0Psnack=Mean(AveEI_kcal0P.1, AveEI_kcal0P.2, AveEI_kcal0P.3, AveEI_kcal0P.4, AveEI_kcal0P.5, AveEI_kcal0P.6, AveEI_kcal0P.7) .

VAR LAB MeanEI_kcal0Psnack 'Mean mean amount eaten (kcal) per snack eating occasion in a day over 7 days'.

COMPUTE MeanEI_kJ0Psnack=Mean(AveEI_kJ0P.1, AveEI_kJ0P.2, AveEI_kJ0P.3, AveEI_kJ0P.4, AveEI_kJ0P.5, AveEI_kJ0P.6, AveEI_kJ0P.7) .

VAR LAB MeanEI_kJ0Psnack 'Mean mean amount eaten (kJ) per snack eating occasion in a day over 7 days'.

COMPUTE SDGrams0Psnack=SD(AveGrams0P.1, AveGrams0P.2, AveGrams0P.3, AveGrams0P.4, AveGrams0P.5, AveGrams0P.6, AveGrams0P.7) .

VAR LAB SDGrams0Psnack 'SD mean amount eaten (g) per snack eating occasion in a day over 7 days'.

COMPUTE SDEI_kcal0Psnack=SD(AveEI_kcal0P.1, AveEI_kcal0P.2, AveEI_kcal0P.3, AveEI_kcal0P.4, AveEI_kcal0P.5, AveEI_kcal0P.6, AveEI_kcal0P.7) .

VAR LAB SDEI_kcal0Psnack 'SD mean amount eaten (kcal) per snack eating occasion in a day over 7 days'.

COMPUTE SDEI_kJ0Psnack=SD(AveEI_kJ0P.1, AveEI_kJ0P.2, AveEI_kJ0P.3, AveEI_kJ0P.4, AveEI_kJ0P.5, AveEI_kJ0P.6, AveEI_kJ0P.7) .

VAR LAB SDEI_kJ0Psnack 'SD mean amount eaten (kJ) per snack eating occasion in a day over 7 days'.

COMPUTE medianGrams0Psnack=median(AveGrams0P.1, AveGrams0P.2, AveGrams0P.3, AveGrams0P.4, AveGrams0P.5, AveGrams0P.6, AveGrams0P.7) .

VAR LAB medianGrams0Psnack 'median mean amount eaten (g) per snack eating occasion in a day over 7 days'.

COMPUTE medianEI_kcal0Psnack=median(AveEI_kcal0P.1, AveEI_kcal0P.2, AveEI_kcal0P.3, AveEI_kcal0P.4, AveEI_kcal0P.5, AveEI_kcal0P.6, AveEI_kcal0P.7) .

VAR LAB medianEI_kcal0Psnack 'median mean amount eaten (kcal) per snack eating occasion in a day over 7 days'.

COMPUTE medianEI_kJ0Psnack=median(AveEI_kJ0P.1, AveEI_kJ0P.2, AveEI_kJ0P.3, AveEI_kJ0P.4, AveEI_kJ0P.5, AveEI_kJ0P.6, AveEI_kJ0P.7) .

VAR LAB medianEI_kJ0Psnack 'median mean amount eaten (kJ) per snack eating occasion in a day over 7 days'.

COMPUTE minGrams0Psnack=min(AveGrams0P.1, AveGrams0P.2, AveGrams0P.3, AveGrams0P.4, AveGrams0P.5, AveGrams0P.6, AveGrams0P.7) .

VAR LAB minGrams0Psnack 'min mean amount eaten (g) per snack eating occasion in a day over 7 days'.

COMPUTE minEI_kcal0Psnack=min(AveEI_kcal0P.1, AveEI_kcal0P.2, AveEI_kcal0P.3, AveEI_kcal0P.4, AveEI_kcal0P.5, AveEI_kcal0P.6, AveEI_kcal0P.7) .

VAR LAB minEI_kcal0Psnack 'min mean amount eaten (kcal) per snack eating occasion in a day over 7 days'.

COMPUTE minEI_kJ0Psnack=min(AveEI_kJ0P.1, AveEI_kJ0P.2, AveEI_kJ0P.3, AveEI_kJ0P.4, AveEI_kJ0P.5, AveEI_kJ0P.6, AveEI_kJ0P.7) .

VAR LAB minEI_kJ0Psnack 'min mean amount eaten (kJ) per snack eating occasion in a day over 7 days'.

COMPUTE maxGrams0Psnack=max(AveGrams0P.1, AveGrams0P.2, AveGrams0P.3, AveGrams0P.4, AveGrams0P.5, AveGrams0P.6, AveGrams0P.7) .

VAR LAB maxGrams0Psnack 'max mean amount eaten (g) per snack eating occasion in a day over 7 days'.

COMPUTE maxEI_kcal0Psnack=max(AveEI_kcal0P.1, AveEI_kcal0P.2, AveEI_kcal0P.3, AveEI_kcal0P.4, AveEI_kcal0P.5, AveEI_kcal0P.6, AveEI_kcal0P.7) .

VAR LAB maxEI_kcal0Psnack 'max mean amount eaten (kcal) per snack eating occasion in a day over 7 days'.

COMPUTE maxEI_kJ0Psnack=max(AveEI_kJ0P.1, AveEI_kJ0P.2, AveEI_kJ0P.3, AveEI_kJ0P.4, AveEI_kJ0P.5, AveEI_kJ0P.6, AveEI_kJ0P.7) .

VAR LAB maxEI_kJ0Psnack 'max mean amount eaten (kJ) per snack eating occasion in a day over 7 days'.

COMPUTE meanSDGrams0Psnack=Mean(SDGrams0P.1, SDGrams0P.2, SDGrams0P.3, SDGrams0P.4, SDGrams0P.5, SDGrams0P.6, SDGrams0P.7) .

VAR LAB meanSDGrams0Psnack 'Mean variation in amount eaten (g) per snack eating occasion in a day over 7 days'.

COMPUTE meanSDEI_kcal0Psnack=Mean(SDEI_kcal0P.1, SDEI_kcal0P.2, SDEI_kcal0P.3, SDEI_kcal0P.4, SDEI_kcal0P.5, SDEI_kcal0P.6, SDEI_kcal0P.7) .

VAR LAB meanSDEI_kcal0Psnack 'Mean variation in amount eaten (kcal) per snack eating occasion in a day over 7 days'.

COMPUTE meanSDEI_kJ0Psnack=Mean(SDEI_kJ0P.1, SDEI_kJ0P.2, SDEI_kJ0P.3, SDEI_kJ0P.4, SDEI_kJ0P.5, SDEI_kJ0P.6, SDEI_kJ0P.7) .

VAR LAB meanSDEI_kJ0Psnack 'Mean variation in amount eaten (kJ) per snack eating occasion in a day over 7 days'.

COMPUTE MedGrams0Psnack=MEDIAN(MedGrams0P.1, MedGrams0P.2, MedGrams0P.3, MedGrams0P.4, MedGrams0P.5, MedGrams0P.6, MedGrams0P.7) .

VAR LAB MedGrams0Psnack 'Median amount eaten (g) per snack eating occasion in a day over 7 days'.

COMPUTE MedEI_kcal0Psnack=MEDIAN(MedEI_kcal0P.1, MedEI_kcal0P.2, MedEI_kcal0P.3, MedEI_kcal0P.4, MedEI_kcal0P.5, MedEI_kcal0P.6, MedEI_kcal0P.7) .

VAR LAB MedEI_kcal0Psnack 'Median amount eaten (kcal) per snack eating occasion in a day over 7 days'.

COMPUTE MedEI_kJ0Psnack=MEDIAN(MedEI_kJ0P.1, MedEI_kJ0P.2, MedEI_kJ0P.3, MedEI_kJ0P.4, MedEI_kJ0P.5, MedEI_kJ0P.6, MedEI_kJ0P.7) .

VAR LAB MedEI_kJ0Psnack 'Median amount eaten (kJ) per snack eating occasion in a day over 7 days'.

COMPUTE MinGrams0Psnack=MEDIAN(MinGrams0P.1, MinGrams0P.2, MinGrams0P.3, MinGrams0P.4, MinGrams0P.5, MinGrams0P.6, MinGrams0P.7) .

VAR LAB MinGrams0Psnack 'Median minimum amount eaten (g) per snack eating occasion in a day over 7 days'.

COMPUTE MinEI_kcal0Psnack=MEDIAN(MinEI_kcal0P.1, MinEI_kcal0P.2, MinEI_kcal0P.3, MinEI_kcal0P.4, MinEI_kcal0P.5, MinEI_kcal0P.6, MinEI_kcal0P.7) .

VAR LAB MinEI_kcal0Psnack 'Median minimum amount eaten (kcal) per snack eating occasion in a day over 7 days'.

COMPUTE MinEI_kJ0Psnack=MEDIAN(MinEI_kJ0P.1, MinEI_kJ0P.2, MinEI_kJ0P.3, MinEI_kJ0P.4, MinEI_kJ0P.5, MinEI_kJ0P.6, MinEI_kJ0P.7) .

VAR LAB MinEI_kJ0Psnack 'Median minimum amount eaten (kJ) per snack eating occasion in a day over 7 days'.

COMPUTE MaxGrams0Psnack=MEDIAN(MaxGrams0P.1, MaxGrams0P.2, MaxGrams0P.3, MaxGrams0P.4, MaxGrams0P.5, MaxGrams0P.6, MaxGrams0P.7) .

VAR LAB MaxGrams0Psnack 'Median maximum amount eaten (g) per snack eating occasion in a day over 7 days'.

COMPUTE MaxEI_kcal0Psnack=MEDIAN(MaxEI_kcal0P.1, MaxEI_kcal0P.2, MaxEI_kcal0P.3, MaxEI_kcal0P.4, MaxEI_kcal0P.5, MaxEI_kcal0P.6, MaxEI_kcal0P.7) .

VAR LAB MaxEI_kcal0Psnack 'Median maximum amount eaten (kcal) per snack eating occasion in a day over 7 days'.

COMPUTE MaxEI_kJ0Psnack=MEDIAN(MaxEI_kJ0P.1, MaxEI_kJ0P.2, MaxEI_kJ0P.3, MaxEI_kJ0P.4, MaxEI_kJ0P.5, MaxEI_kJ0P.6, MaxEI_kJ0P.7) .

VAR LAB MaxEI_kJ0Psnack 'Median maximum amount eaten (kJ) per snack eating occasion in a day over 7 days'.

COMPUTE range_Grams0Psnack=MEDIAN(range_Grams0P.1, range_Grams0P.2, range_Grams0P.3, range_Grams0P.4, range_Grams0P.5, range_Grams0P.6, range_Grams0P.7) .

VAR LAB range_Grams0Psnack 'Median range amount eaten (g) per snack eating occasion in a day over 7 days'.

COMPUTE range_EI_kcal0Psnack=MEDIAN(range_EI_kcal0P.1, range_EI_kcal0P.2, range_EI_kcal0P.3, range_EI_kcal0P.4, range_EI_kcal0P.5, range_EI_kcal0P.6, range_EI_kcal0P.7) .

VAR LAB range_EI_kcal0Psnack 'Median range amount eaten (kcal) per snack eating occasion in a day over 7 days'.

COMPUTE range_EI_kJ0Psnack=MEDIAN(range_EI_kJ0P.1, range_EI_kJ0P.2, range_EI_kJ0P.3, range_EI_kJ0P.4, range_EI_kJ0P.5, range_EI_kJ0P.6, range_EI_kJ0P.7) .

VAR LAB range_EI_kJ0Psnack 'Median range amount eaten (kJ) per snack eating occasion in a day over 7 days'.

EXECUTE.

SORT CASES BY caseid(A).

***Merge new variables into eating architecture variables file.

MATCH FILES /FILE=*

/FILE='N:\NDNS pre 2000\NDNS 19-64 yrs 2000-2001\Eating Architecture.sav'

/BY caseid.

EXECUTE.

SORT CASES BY caseid(A).

SAVE OUTFILE='N:\NDNS pre 2000\NDNS 19-64 yrs 2000-2001\Eating Architecture.sav'

/DROP=dayno.1 dayno.2 dayno.3 dayno.4 dayno.5 dayno.6 dayno.7 AveGrams0P.1 AveGrams0P.2 AveGrams0P.3 AveGrams0P.4 AveGrams0P.5 AveGrams0P.6 AveGrams0P.7

AveEI_kcal0P.1 AveEI_kcal0P.2 AveEI_kcal0P.3 AveEI_kcal0P.4 AveEI_kcal0P.5 AveEI_kcal0P.6 AveEI_kcal0P.7 AveEI_kJ0P.1 AveEI_kJ0P.2 AveEI_kJ0P.3 AveEI_kJ0P.4 AveEI_kJ0P.5 AveEI_kJ0P.6 AveEI_kJ0P.7

SDGrams0P.1 SDGrams0P.2 SDGrams0P.3 SDGrams0P.4 SDGrams0P.5 SDGrams0P.6 SDGrams0P.7 SDEI_kcal0P.1 SDEI_kcal0P.2 SDEI_kcal0P.3 SDEI_kcal0P.4 SDEI_kcal0P.5 SDEI_kcal0P.6 SDEI_kcal0P.7

SDEI_kJ0P.1 SDEI_kJ0P.2 SDEI_kJ0P.3 SDEI_kJ0P.4 SDEI_kJ0P.5 SDEI_kJ0P.6 SDEI_kJ0P.7 MedGrams0P.1 MedGrams0P.2 MedGrams0P.3 MedGrams0P.4 MedGrams0P.5 MedGrams0P.6 MedGrams0P.7

MedEI_kcal0P.1 MedEI_kcal0P.2 MedEI_kcal0P.3 MedEI_kcal0P.4 MedEI_kcal0P.5 MedEI_kcal0P.6 MedEI_kcal0P.7 MedEI_kJ0P.1 MedEI_kJ0P.2 MedEI_kJ0P.3 MedEI_kJ0P.4 MedEI_kJ0P.5 MedEI_kJ0P.6 MedEI_kJ0P.7

MinGrams0P.1 MinGrams0P.2 MinGrams0P.3 MinGrams0P.4 MinGrams0P.5 MinGrams0P.6 MinGrams0P.7 MinEI_kcal0P.1 MinEI_kcal0P.2 MinEI_kcal0P.3 MinEI_kcal0P.4 MinEI_kcal0P.5 MinEI_kcal0P.6 MinEI_kcal0P.7

MinEI_kJ0P.1 MinEI_kJ0P.2 MinEI_kJ0P.3 MinEI_kJ0P.4 MinEI_kJ0P.5 MinEI_kJ0P.6 MinEI_kJ0P.7 MaxGrams0P.1 MaxGrams0P.2 MaxGrams0P.3 MaxGrams0P.4 MaxGrams0P.5 MaxGrams0P.6 MaxGrams0P.7

MaxEI_kcal0P.1 MaxEI_kcal0P.2 MaxEI_kcal0P.3 MaxEI_kcal0P.4 MaxEI_kcal0P.5 MaxEI_kcal0P.6 MaxEI_kcal0P.7 MaxEI_kJ0P.1 MaxEI_kJ0P.2 MaxEI_kJ0P.3 MaxEI_kJ0P.4 MaxEI_kJ0P.5 MaxEI_kJ0P.6 MaxEI_kJ0P.7

range_Grams0P.1 range_Grams0P.2 range_Grams0P.3 range_Grams0P.4 range_Grams0P.5 range_Grams0P.6 range_Grams0P.7 range_EI_kcal0P.1 range_EI_kcal0P.2 range_EI_kcal0P.3 range_EI_kcal0P.4 range_EI_kcal0P.5

range_EI_kcal0P.6 range_EI_kcal0P.7 range_EI_kJ0P.1 range_EI_kJ0P.2 range_EI_kJ0P.3 range_EI_kJ0P.4 range_EI_kJ0P.5 range_EI_kJ0P.6 range_EI_kJ0P.7

/COMPRESSED.

****Need to compute size variables but to account for days when people eat no meals, snacks or drink we need to first collapse to day level then transpose to person level then recode missing and then compute mean, median, etc.

***Computing average size of drink eating occasions per day***.

GET

FILE='N:\NDNS pre 2000\NDNS 19-64 yrs 2000-2001\period0.sav' .

***Select only eating occasions classified as drinks.

USE ALL.

SELECT IF (Occasion_Type = 3).

EXECUTE.

SORT CASES BY caseid .

***Aggregate size of drink eating occasions in the same day: EO level to day level***.

AGGREGATE

/OUTFILE='N:\NDNS pre 2000\NDNS 19-64 yrs 2000-2001\aggr.sav'

/PRESORTED

/BREAK=caseid dayno

/AveGrams0P 'Average amount eaten (g) per drink eating occasion'=MEAN(Grams)

/AveEI_kcal0P 'Energy intake (kcal) per drink eating occasion'=MEAN(Energy_kcal)

/AveEI_kJ0P 'Energy intake (kJ) per drink eating occasion'=MEAN(Energy_kJ)

/SDGrams0P 'SD of amount eaten (g) per drink eating occasion'=SD(Grams)

/SDEI_kcal0P 'SD of energy intake (kcal) per drink eating occasion'=SD(Energy_kcal)

/SDEI_kJ0P 'SD of energy intake (kJ) per drink eating occasion'=SD(Energy_kJ)

/MedGrams0P 'Median amount eaten (g) per drink eating occasion'=MEDIAN(Grams)

/MedEI_kcal0P 'Median Energy intake (kcal) per drink eating occasion'=MEDIAN(Energy_kcal)

/MedEI_kJ0P 'Median Energy intake (kJ) per drink eating occasion'=MEDIAN(Energy_kJ)

/MinGrams0P 'Minimum amount eaten (g) per drink eating occasion'=MIN(Grams)

/MinEI_kcal0P 'Minimum Energy intake (kcal) per drink eating occasion'=MIN(Energy_kcal)

/MinEI_kJ0P 'Minimum Energy intake (kJ) per drink eating occasion'=MIN(Energy_kJ)

/MaxGrams0P 'Maximum amount eaten (g) per drink eating occasion'=MAX(Grams)

/MaxEI_kcal0P 'Maximum Energy intake (kcal) per drink eating occasion'=MAX(Energy_kcal)

/MaxEI_kJ0P 'Maximum Energy intake (kJ) per drink eating occasion'=MAX(Energy_kJ).

GET

FILE='N:\NDNS pre 2000\NDNS 19-64 yrs 2000-2001\aggr.sav' .

COMPUTE range_Grams0P = MaxGrams0P - MinGrams0P .

COMPUTE range_EI_kcal0P = MaxEI_kcal0P - MinEI_kcal0P .

COMPUTE range_EI_kJ0P = MaxEI_kJ0P - MinEI_kJ0P .

VAR LAB range_Grams0P 'Range of grams per drink over 1 day'.

VAR LAB range_EI_kcal0P 'Range of kcal per drink over 1 day'.

VAR LAB range_EI_kJ0P 'Range of kJ per drink over 1 day'.

**Transposes the file from one line per day to one line per person with features of days as variables.

CASESTOVARS

/ID=caseid

/GROUPBY=VARIABLE

/COUNT=days "Number of days" .

***Replaces any missing values with 0 because these people did report eating on 7 days but simply had 0 meals/snacks/drinks on some days, this appears missing in the long file but should be 0.

RECODE AveGrams0P.1 AveGrams0P.2 AveGrams0P.3 AveGrams0P.4 AveGrams0P.5 AveGrams0P.6 AveGrams0P.7

AveEI_kcal0P.1 AveEI_kcal0P.2 AveEI_kcal0P.3 AveEI_kcal0P.4 AveEI_kcal0P.5 AveEI_kcal0P.6 AveEI_kcal0P.7

AveEI_kJ0P.1 AveEI_kJ0P.2 AveEI_kJ0P.3 AveEI_kJ0P.4 AveEI_kJ0P.5 AveEI_kJ0P.6 AveEI_kJ0P.7

SDGrams0P.1 SDGrams0P.2 SDGrams0P.3 SDGrams0P.4 SDGrams0P.5 SDGrams0P.6 SDGrams0P.7

SDEI_kcal0P.1 SDEI_kcal0P.2 SDEI_kcal0P.3 SDEI_kcal0P.4 SDEI_kcal0P.5 SDEI_kcal0P.6 SDEI_kcal0P.7

SDEI_kJ0P.1 SDEI_kJ0P.2 SDEI_kJ0P.3 SDEI_kJ0P.4 SDEI_kJ0P.5 SDEI_kJ0P.6 SDEI_kJ0P.7

MedGrams0P.1 MedGrams0P.2 MedGrams0P.3 MedGrams0P.4 MedGrams0P.5 MedGrams0P.6 MedGrams0P.7

MedEI_kcal0P.1 MedEI_kcal0P.2 MedEI_kcal0P.3 MedEI_kcal0P.4 MedEI_kcal0P.5 MedEI_kcal0P.6 MedEI_kcal0P.7

MedEI_kJ0P.1 MedEI_kJ0P.2 MedEI_kJ0P.3 MedEI_kJ0P.4 MedEI_kJ0P.5 MedEI_kJ0P.6 MedEI_kJ0P.7

MinGrams0P.1 MinGrams0P.2 MinGrams0P.3 MinGrams0P.4 MinGrams0P.5 MinGrams0P.6 MinGrams0P.7

MinEI_kcal0P.1 MinEI_kcal0P.2 MinEI_kcal0P.3 MinEI_kcal0P.4 MinEI_kcal0P.5 MinEI_kcal0P.6 MinEI_kcal0P.7

MinEI_kJ0P.1 MinEI_kJ0P.2 MinEI_kJ0P.3 MinEI_kJ0P.4 MinEI_kJ0P.5 MinEI_kJ0P.6 MinEI_kJ0P.7

MaxGrams0P.1 MaxGrams0P.2 MaxGrams0P.3 MaxGrams0P.4 MaxGrams0P.5 MaxGrams0P.6 MaxGrams0P.7

MaxEI_kcal0P.1 MaxEI_kcal0P.2 MaxEI_kcal0P.3 MaxEI_kcal0P.4 MaxEI_kcal0P.5 MaxEI_kcal0P.6 MaxEI_kcal0P.7

MaxEI_kJ0P.1 MaxEI_kJ0P.2 MaxEI_kJ0P.3 MaxEI_kJ0P.4 MaxEI_kJ0P.5 MaxEI_kJ0P.6 MaxEI_kJ0P.7

range_Grams0P.1 range_Grams0P.2 range_Grams0P.3 range_Grams0P.4 range_Grams0P.5 range_Grams0P.6 range_Grams0P.7

range_EI_kcal0P.1 range_EI_kcal0P.2 range_EI_kcal0P.3 range_EI_kcal0P.4 range_EI_kcal0P.5 range_EI_kcal0P.6 range_EI_kcal0P.7

range_EI_kJ0P.1 range_EI_kJ0P.2 range_EI_kJ0P.3 range_EI_kJ0P.4 range_EI_kJ0P.5 range_EI_kJ0P.6 range_EI_kJ0P.7

(SYSMIS=0).

COMPUTE MeanGrams0Pdrink=Mean(AveGrams0P.1, AveGrams0P.2, AveGrams0P.3, AveGrams0P.4, AveGrams0P.5, AveGrams0P.6, AveGrams0P.7) .

VAR LAB MeanGrams0Pdrink 'Mean amount eaten (g) per drink eating occasion in a day over 7 days'.

COMPUTE MeanEI_kcal0Pdrink=Mean(AveEI_kcal0P.1, AveEI_kcal0P.2, AveEI_kcal0P.3, AveEI_kcal0P.4, AveEI_kcal0P.5, AveEI_kcal0P.6, AveEI_kcal0P.7) .

VAR LAB MeanEI_kcal0Pdrink 'Mean amount eaten (kcal) per drink eating occasion in a day over 7 days'.

COMPUTE MeanEI_kJ0Pdrink=Mean(AveEI_kJ0P.1, AveEI_kJ0P.2, AveEI_kJ0P.3, AveEI_kJ0P.4, AveEI_kJ0P.5, AveEI_kJ0P.6, AveEI_kJ0P.7) .

VAR LAB MeanEI_kJ0Pdrink 'Mean amount eaten (kJ) per drink eating occasion in a day over 7 days'.

COMPUTE SDGrams0Pdrink=SD(AveGrams0P.1, AveGrams0P.2, AveGrams0P.3, AveGrams0P.4, AveGrams0P.5, AveGrams0P.6, AveGrams0P.7) .

VAR LAB SDGrams0Pdrink 'SD amount eaten (g) per drink eating occasion in a day over 7 days'.

COMPUTE SDEI_kcal0Pdrink=SD(AveEI_kcal0P.1, AveEI_kcal0P.2, AveEI_kcal0P.3, AveEI_kcal0P.4, AveEI_kcal0P.5, AveEI_kcal0P.6, AveEI_kcal0P.7) .

VAR LAB SDEI_kcal0Pdrink 'SD amount eaten (kcal) per drink eating occasion in a day over 7 days'.

COMPUTE SDEI_kJ0Pdrink=SD(AveEI_kJ0P.1, AveEI_kJ0P.2, AveEI_kJ0P.3, AveEI_kJ0P.4, AveEI_kJ0P.5, AveEI_kJ0P.6, AveEI_kJ0P.7) .

VAR LAB SDEI_kJ0Pdrink 'SD mean amount eaten (kJ) per drink eating occasion in a day over 7 days'.

COMPUTE medianGrams0Pdrink=median(AveGrams0P.1, AveGrams0P.2, AveGrams0P.3, AveGrams0P.4, AveGrams0P.5, AveGrams0P.6, AveGrams0P.7) .

VAR LAB medianGrams0Pdrink 'median mean amount eaten (g) per drink eating occasion in a day over 7 days'.

COMPUTE medianEI_kcal0Pdrink=median(AveEI_kcal0P.1, AveEI_kcal0P.2, AveEI_kcal0P.3, AveEI_kcal0P.4, AveEI_kcal0P.5, AveEI_kcal0P.6, AveEI_kcal0P.7) .

VAR LAB medianEI_kcal0Pdrink 'median mean amount eaten (kcal) per drink eating occasion in a day over 7 days'.

COMPUTE medianEI_kJ0Pdrink=median(AveEI_kJ0P.1, AveEI_kJ0P.2, AveEI_kJ0P.3, AveEI_kJ0P.4, AveEI_kJ0P.5, AveEI_kJ0P.6, AveEI_kJ0P.7) .

VAR LAB medianEI_kJ0Pdrink 'median mean amount eaten (kJ) per drink eating occasion in a day over 7 days'.

COMPUTE minGrams0Pdrink=min(AveGrams0P.1, AveGrams0P.2, AveGrams0P.3, AveGrams0P.4, AveGrams0P.5, AveGrams0P.6, AveGrams0P.7) .

VAR LAB minGrams0Pdrink 'min mean amount eaten (g) per drink eating occasion in a day over 7 days'.

COMPUTE minEI_kcal0Pdrink=min(AveEI_kcal0P.1, AveEI_kcal0P.2, AveEI_kcal0P.3, AveEI_kcal0P.4, AveEI_kcal0P.5, AveEI_kcal0P.6, AveEI_kcal0P.7) .

VAR LAB minEI_kcal0Pdrink 'min mean amount eaten (kcal) per drink eating occasion in a day over 7 days'.

COMPUTE minEI_kJ0Pdrink=min(AveEI_kJ0P.1, AveEI_kJ0P.2, AveEI_kJ0P.3, AveEI_kJ0P.4, AveEI_kJ0P.5, AveEI_kJ0P.6, AveEI_kJ0P.7) .

VAR LAB minEI_kJ0Pdrink 'min mean amount eaten (kJ) per drink eating occasion in a day over 7 days'.

COMPUTE maxGrams0Pdrink=max(AveGrams0P.1, AveGrams0P.2, AveGrams0P.3, AveGrams0P.4, AveGrams0P.5, AveGrams0P.6, AveGrams0P.7) .

VAR LAB maxGrams0Pdrink 'max mean amount eaten (g) per drink eating occasion in a day over 7 days'.

COMPUTE maxEI_kcal0Pdrink=max(AveEI_kcal0P.1, AveEI_kcal0P.2, AveEI_kcal0P.3, AveEI_kcal0P.4, AveEI_kcal0P.5, AveEI_kcal0P.6, AveEI_kcal0P.7) .

VAR LAB maxEI_kcal0Pdrink 'max mean amount eaten (kcal) per drink eating occasion in a day over 7 days'.

COMPUTE maxEI_kJ0Pdrink=max(AveEI_kJ0P.1, AveEI_kJ0P.2, AveEI_kJ0P.3, AveEI_kJ0P.4, AveEI_kJ0P.5, AveEI_kJ0P.6, AveEI_kJ0P.7) .

VAR LAB maxEI_kJ0Pdrink 'max mean amount eaten (kJ) per drink eating occasion in a day over 7 days'.

COMPUTE meanSDGrams0Pdrink=Mean(SDGrams0P.1, SDGrams0P.2, SDGrams0P.3, SDGrams0P.4, SDGrams0P.5, SDGrams0P.6, SDGrams0P.7) .

VAR LAB meanSDGrams0Pdrink 'Mean variation in amount eaten (g) per drink eating occasion in a day over 7 days'.

COMPUTE meanSDEI_kcal0Pdrink=Mean(SDEI_kcal0P.1, SDEI_kcal0P.2, SDEI_kcal0P.3, SDEI_kcal0P.4, SDEI_kcal0P.5, SDEI_kcal0P.6, SDEI_kcal0P.7) .

VAR LAB meanSDEI_kcal0Pdrink 'Mean variation in amount eaten (kcal) per drink eating occasion in a day over 7 days'.

COMPUTE meanSDEI_kJ0Pdrink=Mean(SDEI_kJ0P.1, SDEI_kJ0P.2, SDEI_kJ0P.3, SDEI_kJ0P.4, SDEI_kJ0P.5, SDEI_kJ0P.6, SDEI_kJ0P.7) .

VAR LAB meanSDEI_kJ0Pdrink 'Mean variation in amount eaten (kJ) per drink eating occasion in a day over 7 days'.

COMPUTE MedGrams0Pdrink=MEDIAN(MedGrams0P.1, MedGrams0P.2, MedGrams0P.3, MedGrams0P.4, MedGrams0P.5, MedGrams0P.6, MedGrams0P.7) .

VAR LAB MedGrams0Pdrink 'Median amount eaten (g) per drink eating occasion in a day over 7 days'.

COMPUTE MedEI_kcal0Pdrink=MEDIAN(MedEI_kcal0P.1, MedEI_kcal0P.2, MedEI_kcal0P.3, MedEI_kcal0P.4, MedEI_kcal0P.5, MedEI_kcal0P.6, MedEI_kcal0P.7) .

VAR LAB MedEI_kcal0Pdrink 'Median amount eaten (kcal) per drink eating occasion in a day over 7 days'.

COMPUTE MedEI_kJ0Pdrink=MEDIAN(MedEI_kJ0P.1, MedEI_kJ0P.2, MedEI_kJ0P.3, MedEI_kJ0P.4, MedEI_kJ0P.5, MedEI_kJ0P.6, MedEI_kJ0P.7) .

VAR LAB MedEI_kJ0Pdrink 'Median amount eaten (kJ) per drink eating occasion in a day over 7 days'.

COMPUTE MinGrams0Pdrink=MEDIAN(MinGrams0P.1, MinGrams0P.2, MinGrams0P.3, MinGrams0P.4, MinGrams0P.5, MinGrams0P.6, MinGrams0P.7) .

VAR LAB MinGrams0Pdrink 'Median minimum amount eaten (g) per drink eating occasion in a day over 7 days'.

COMPUTE MinEI_kcal0Pdrink=MEDIAN(MinEI_kcal0P.1, MinEI_kcal0P.2, MinEI_kcal0P.3, MinEI_kcal0P.4, MinEI_kcal0P.5, MinEI_kcal0P.6, MinEI_kcal0P.7) .

VAR LAB MinEI_kcal0Pdrink 'Median minimum amount eaten (kcal) per drink eating occasion in a day over 7 days'.

COMPUTE MinEI_kJ0Pdrink=MEDIAN(MinEI_kJ0P.1, MinEI_kJ0P.2, MinEI_kJ0P.3, MinEI_kJ0P.4, MinEI_kJ0P.5, MinEI_kJ0P.6, MinEI_kJ0P.7) .

VAR LAB MinEI_kJ0Pdrink 'Median minimum amount eaten (kJ) per drink eating occasion in a day over 7 days'.

COMPUTE MaxGrams0Pdrink=MEDIAN(MaxGrams0P.1, MaxGrams0P.2, MaxGrams0P.3, MaxGrams0P.4, MaxGrams0P.5, MaxGrams0P.6, MaxGrams0P.7) .

VAR LAB MaxGrams0Pdrink 'Median maximum amount eaten (g) per drink eating occasion in a day over 7 days'.

COMPUTE MaxEI_kcal0Pdrink=MEDIAN(MaxEI_kcal0P.1, MaxEI_kcal0P.2, MaxEI_kcal0P.3, MaxEI_kcal0P.4, MaxEI_kcal0P.5, MaxEI_kcal0P.6, MaxEI_kcal0P.7) .

VAR LAB MaxEI_kcal0Pdrink 'Median maximum amount eaten (kcal) per drink eating occasion in a day over 7 days'.

COMPUTE MaxEI_kJ0Pdrink=MEDIAN(MaxEI_kJ0P.1, MaxEI_kJ0P.2, MaxEI_kJ0P.3, MaxEI_kJ0P.4, MaxEI_kJ0P.5, MaxEI_kJ0P.6, MaxEI_kJ0P.7) .

VAR LAB MaxEI_kJ0Pdrink 'Median maximum amount eaten (kJ) per drink eating occasion in a day over 7 days'.

COMPUTE range_Grams0Pdrink=MEDIAN(range_Grams0P.1, range_Grams0P.2, range_Grams0P.3, range_Grams0P.4, range_Grams0P.5, range_Grams0P.6, range_Grams0P.7) .

VAR LAB range_Grams0Pdrink 'Median range amount eaten (g) per drink eating occasion in a day over 7 days'.

COMPUTE range_EI_kcal0Pdrink=MEDIAN(range_EI_kcal0P.1, range_EI_kcal0P.2, range_EI_kcal0P.3, range_EI_kcal0P.4, range_EI_kcal0P.5, range_EI_kcal0P.6, range_EI_kcal0P.7) .

VAR LAB range_EI_kcal0Pdrink 'Median range amount eaten (kcal) per drink eating occasion in a day over 7 days'.

COMPUTE range_EI_kJ0Pdrink=MEDIAN(range_EI_kJ0P.1, range_EI_kJ0P.2, range_EI_kJ0P.3, range_EI_kJ0P.4, range_EI_kJ0P.5, range_EI_kJ0P.6, range_EI_kJ0P.7) .

VAR LAB range_EI_kJ0Pdrink 'Median range amount eaten (kJ) per drink eating occasion in a day over 7 days'.

EXECUTE.

SORT CASES BY caseid(A).

***Merge new variables into eating architecture variables file.

MATCH FILES /FILE=*

/FILE='N:\NDNS pre 2000\NDNS 19-64 yrs 2000-2001\Eating Architecture.sav'

/BY caseid.

EXECUTE.

SORT CASES BY caseid(A).

SAVE OUTFILE='N:\NDNS pre 2000\NDNS 19-64 yrs 2000-2001\Eating Architecture.sav'

/DROP=dayno.1 dayno.2 dayno.3 dayno.4 dayno.5 dayno.6 dayno.7 AveGrams0P.1 AveGrams0P.2 AveGrams0P.3 AveGrams0P.4 AveGrams0P.5 AveGrams0P.6 AveGrams0P.7

AveEI_kcal0P.1 AveEI_kcal0P.2 AveEI_kcal0P.3 AveEI_kcal0P.4 AveEI_kcal0P.5 AveEI_kcal0P.6 AveEI_kcal0P.7 AveEI_kJ0P.1 AveEI_kJ0P.2 AveEI_kJ0P.3 AveEI_kJ0P.4 AveEI_kJ0P.5 AveEI_kJ0P.6 AveEI_kJ0P.7

SDGrams0P.1 SDGrams0P.2 SDGrams0P.3 SDGrams0P.4 SDGrams0P.5 SDGrams0P.6 SDGrams0P.7 SDEI_kcal0P.1 SDEI_kcal0P.2 SDEI_kcal0P.3 SDEI_kcal0P.4 SDEI_kcal0P.5 SDEI_kcal0P.6 SDEI_kcal0P.7

SDEI_kJ0P.1 SDEI_kJ0P.2 SDEI_kJ0P.3 SDEI_kJ0P.4 SDEI_kJ0P.5 SDEI_kJ0P.6 SDEI_kJ0P.7 MedGrams0P.1 MedGrams0P.2 MedGrams0P.3 MedGrams0P.4 MedGrams0P.5 MedGrams0P.6 MedGrams0P.7

MedEI_kcal0P.1 MedEI_kcal0P.2 MedEI_kcal0P.3 MedEI_kcal0P.4 MedEI_kcal0P.5 MedEI_kcal0P.6 MedEI_kcal0P.7 MedEI_kJ0P.1 MedEI_kJ0P.2 MedEI_kJ0P.3 MedEI_kJ0P.4 MedEI_kJ0P.5 MedEI_kJ0P.6 MedEI_kJ0P.7

MinGrams0P.1 MinGrams0P.2 MinGrams0P.3 MinGrams0P.4 MinGrams0P.5 MinGrams0P.6 MinGrams0P.7 MinEI_kcal0P.1 MinEI_kcal0P.2 MinEI_kcal0P.3 MinEI_kcal0P.4 MinEI_kcal0P.5 MinEI_kcal0P.6 MinEI_kcal0P.7

MinEI_kJ0P.1 MinEI_kJ0P.2 MinEI_kJ0P.3 MinEI_kJ0P.4 MinEI_kJ0P.5 MinEI_kJ0P.6 MinEI_kJ0P.7 MaxGrams0P.1 MaxGrams0P.2 MaxGrams0P.3 MaxGrams0P.4 MaxGrams0P.5 MaxGrams0P.6 MaxGrams0P.7

MaxEI_kcal0P.1 MaxEI_kcal0P.2 MaxEI_kcal0P.3 MaxEI_kcal0P.4 MaxEI_kcal0P.5 MaxEI_kcal0P.6 MaxEI_kcal0P.7 MaxEI_kJ0P.1 MaxEI_kJ0P.2 MaxEI_kJ0P.3 MaxEI_kJ0P.4 MaxEI_kJ0P.5 MaxEI_kJ0P.6 MaxEI_kJ0P.7

range_Grams0P.1 range_Grams0P.2 range_Grams0P.3 range_Grams0P.4 range_Grams0P.5 range_Grams0P.6 range_Grams0P.7 range_EI_kcal0P.1 range_EI_kcal0P.2 range_EI_kcal0P.3 range_EI_kcal0P.4 range_EI_kcal0P.5

range_EI_kcal0P.6 range_EI_kcal0P.7 range_EI_kJ0P.1 range_EI_kJ0P.2 range_EI_kJ0P.3 range_EI_kJ0P.4 range_EI_kJ0P.5 range_EI_kJ0P.6 range_EI_kJ0P.7

/COMPRESSED.

GET

FILE='\\rdsfcifs.acrc.bris.ac.uk\NDNS_analyses\NDNS pre 2000\NDNS 19-64 yrs 2000-2001\Eating Architecture.sav'.

SORT CASES BY caseid(A).

SAVE OUTFILE='\\rdsfcifs.acrc.bris.ac.uk\NDNS_analyses\NDNS pre 2000\NDNS 19-64 yrs 2000-2001\Eating Architecture_Manos.sav'

/KEEP=caseid n_occasions_mean n_Meal_mean n_snack_mean n_drink_mean AveGrams0P AveEI_kcal0P AveEI_kJ0P MeanGrams0Pmeal MeanEI_kcal0Pmeal MeanEI_kJ0Pmeal MeanGrams0Psnack

MeanEI_kcal0Psnack MeanEI_kJ0Psnack MeanGrams0Pdrink MeanEI_kcal0Pdrink MeanEI_kJ0Pdrink EatingPeriod_mean TimeFirst_mean TimeLast_mean mealEatingPeriod_mean TimeFirstmeal_mean

TimeLastmeal_mean snackEatingPeriod_mean TimeFirstsnack_mean TimeLastsnack_mean drinkEatingPeriod_mean TimeFirstdrink_mean TimeLastdrink_mean

/COMPRESSED.
